# Antiplasmodial Ealapasamines A-C,‘Mixed’ Naphthylisoquinoline Dimers from the Central African Liana *Ancistrocladus ealaensis*

**DOI:** 10.1038/s41598-017-05719-w

**Published:** 2017-07-18

**Authors:** Dieudonné Tshitenge Tshitenge, Doris Feineis, Virima Mudogo, Marcel Kaiser, Reto Brun, Gerhard Bringmann

**Affiliations:** 1 Institute of Organic Chemistry, University of Würzburg, Am Hubland, D-97074 Würzburg, Germany; 2 Faculty of Pharmaceutical Sciences, University of Kinshasa, B.P. 212 Kinshasa XI, Democratic Republic of the Congo; 3 Faculty of Sciences, University of Kinshasa, B.P. 202 Kinshasa XI, Democratic Republic of the Congo; 4 Swiss Tropical and Public Health Institute, Socinstrasse 57, CH-4002 Basel, Switzerland; 5 University of Basel, Petersplatz 1, CH-4003 Basel, Switzerland

## Abstract

Three unusual heterodimeric naphthylisoquinoline alkaloids, named ealapasamines A-C (1–3), were isolated from the leaves of the tropical plant *Ancistrocladus ealaensis* J. Léonard. These ‘mixed’, constitutionally unsymmetric dimers are the first stereochemically fully assigned cross-coupling products of a 5,8′- and a 7,8′-coupled naphthylisoquinoline linked *via* C-6′ in both naphthalene portions. So far, only two other West and Central *Ancistrocladus* species were known to produce dimers with a central 6,6″-axis, yet, in contrast to the ealapasamines, usually consisting of two 5,8′-coupled monomers, like *e.g*., in michellamine B. The new dimers 1–3 contain six elements of chirality, four stereogenic centers and the two outer axes, while the central biaryl axis is configurationally unstable. The elucidation of the complete stereostructures of the ealapasamines was achieved by the interplay of spectroscopic methods including HRESIMS, 1D and 2D NMR (in particular ROESY measurements), in combination with chemical (oxidative degradation) and chiroptical (electronic circular dichroism) investigations. The ealapasamines A-C display high antiplasmodial activities with excellent half-maximum inhibition concentration values in the low nanomolar range.

## Introduction

Naphthylisoquinoline alkaloids^1^ from tropical Ancistrocladaceae and Dioncophyllaceae lianas are the first known tetrahydroisoquinolines of polyketidic origin^2^. They consist of an isoquinoline and a naphthalene part, usually linked by a rotationally hindered *C,C*- or *N,C*-axis. More than 180 such alkaloids have meanwhile been isolated, showing a broad structural diversity^1, 3, 4^. Most remarkable is the ability of some *Ancistrocladus* species to produce dimers^1, 5–9^, thus giving rise to thrilling quateraryls with unique molecular architectures, possessing up to four stereocenters and three biaryl axes. Some of these compounds show significant anti-HIV effects^1, 7–9^, while others exhibit pronounced activities against pathogens causing different tropical diseases like *e.g*., malaria^5, 6, 10^.

**Figure 1:**
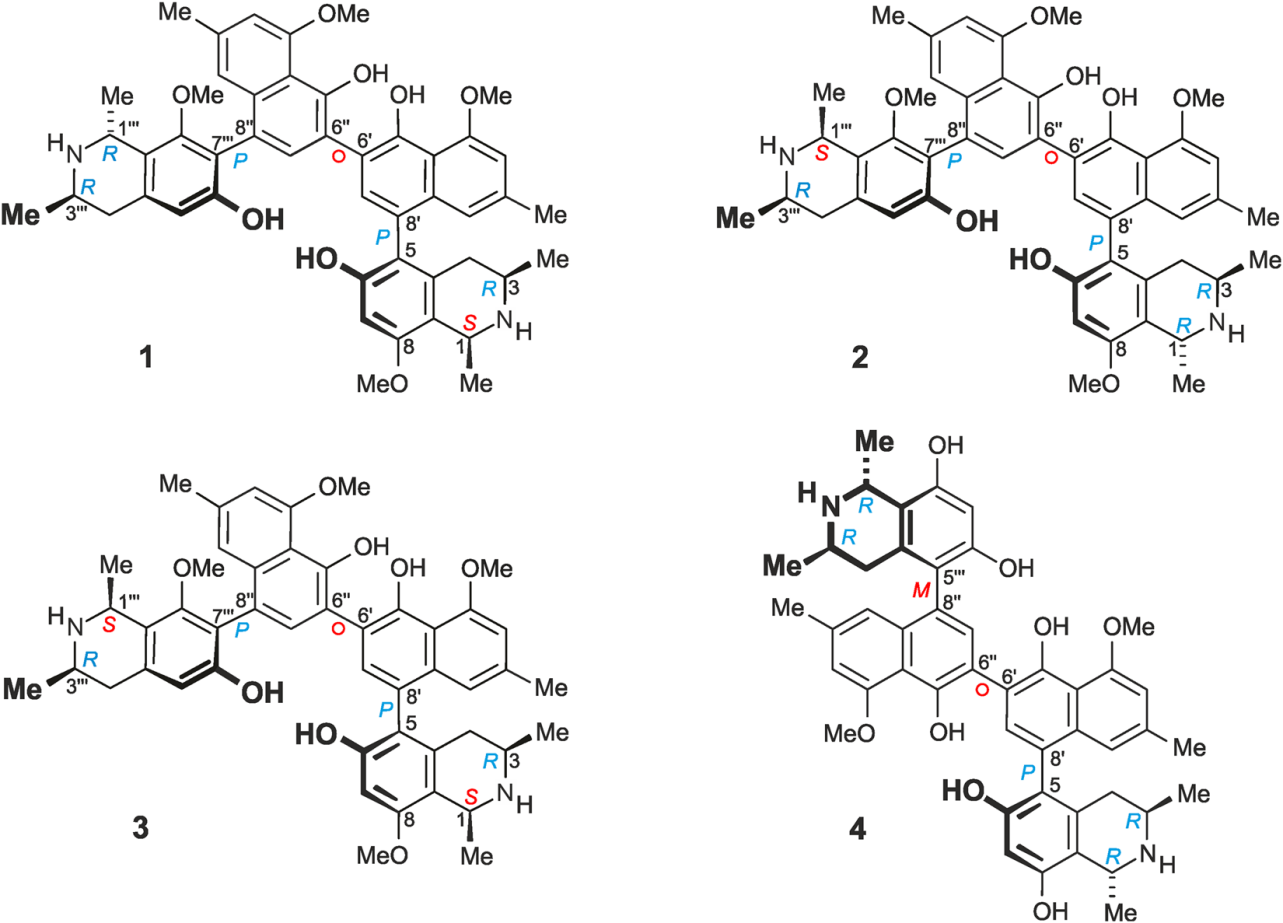
Ealapasamines (A–C) (**1**–**3**) from *A. ealaensis*, and michellamine (B) (**4**) from *A. korupensis*.

During the past years, the Cameroonian liana *A. korupensis* ^7, 8^ and the Congolese species *A. congolensis* ^9^ have attracted particular attention as a rich source of dimeric naphthylisoquinoline alkaloids with a 6′,6″-coupled central biaryl linkage. From these two species, eleven such dimers have so far been discovered, among them michellamine B (**4**) (Fig. 1), which consists of two 5,8′-coupled molecular halves.

We herein report on the isolation and structural elucidation of three new, structurally unique heterodimeric naphthylisoquinoline alkaloids, named ealapasamines A (**1**), B (**2**), and C (**3**) (Fig. 1). These secondary metabolites were isolated from the leaves of *Ancistrocladus ealaensis* Léonard ^1, 11^, a Central African liana mainly occurring in the Northwestern part of the Democratic Republic of the Congo. The chemical constituents of this plant have so far been studied only scarcely. Earlier work on *A. ealaensis* ^12^ has only led to the isolation of the two 5,8′-coupled naphthylisoquinolines ancistroealaines A and B, and of three biosynthetically related naphthoic acid derivatives.

The new compounds **1**–**3**, by contrast, are ‘mixed’, constitutionally unsymmetric dimers consisting of two monomeric parts that are – as for the michellamines – linked *via* C-6′ of both of the two naphthalene portions. In contrast to all those michellamine-type compounds, however, the ealapasamines A–C are highly unsymmetric, built up from a 5,8′-coupled naphthylisoquinoline and a 7,8′-coupled moiety. As a consequence, they are the first fully assigned heterodimers possessing three different biaryl linkages, and thus show a molecular framework that is substantially different from those of michellamine-type dimers.

The new ealapasamines A–C (**1**–**3**) were tested against the pathogens causing malaria tropica, leishmaniasis, Chagas’s disease, and African sleeping sickness, showing remarkably high activities against *Plasmodium falciparum*.

## Results and Discussion

### Isolation

LC-MS guided analysis of a crude leaf extract of *A. ealaensis* revealed the presence of further constituents, with MS profiles typical of dimeric naphthylisoquinoline alkaloids. For the isolation of these compounds, ground leaves were macerated with MeOH, and further partitioned between water and dichloromethane to extract the metabolites. Fractionation of the organic layer by reversed-phase HPLC provided three new dimers.

### Structural elucidation of compounds 1–3

#### Ealapasamine A (1)

**Figure 2:**
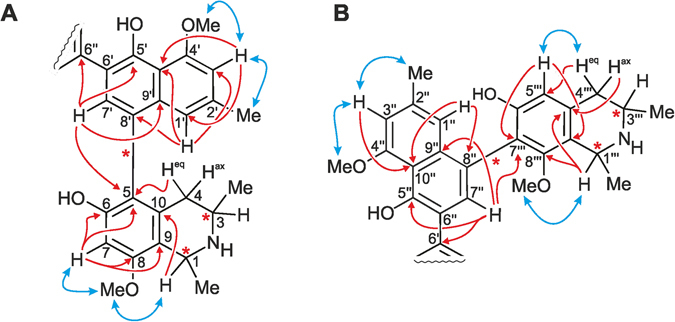
HMBC (red arrows) and ROESY (blue arrows) interactions indicative of the constitutions of the southeastern (**A**) and the northwestern (**B**) moieties of 1.

The first dimer, with a determined molecular formula of C_48_H_52_N_2_O_8_ by HRESIMS, was obtained as a colorless solid. The ^1^H NMR spectrum showed a full set of signals, indicative of an unsymmetric dimer. DEPT-135, HSQC, HMBC, and COSY data (see Supplementary information, SI, Tables 1–4) hinted at the presence of 24 protonated carbon atoms, among them eight aromatic methine groups belonging to six spin systems, two methylene functions, and four aromatic *O*-methyl groups (Fig. 2). The unsymmetric structure, as also confirmed by the occurrence of 48 signals in the ^13^C NMR spectrum, excluded that the alkaloid was the known symmetric dimer ancistrogriffithine A^13^, which has the same molecular formula. Moreover, the presence of the only other unsymmetric dimers known from nature possessing the same molecular formula, mbandakamines A and B^6^, was easily ruled out since the naphthylisoquinoline alkaloid now discovered in *A. ealaensis* showed substantially different 1D and 2D NMR spectra, thus evidencing that the isolated dimer was new.

The ‘southeastern’ half of the new dimer displayed a total of four aromatic protons in the ^1^H NMR spectrum (see Table 1). This portion furthermore showed the presence of one aromatic singlet, H-7′ (*δ*_H_ 7.28), two aromatic protons with a *meta*-coupling pattern, H-1′ (*δ*_H_ 6.80, d, *J* = 1.18 Hz) and H-3′ (*δ*_H_ 6.87, d, *J* = 1.22 Hz), one aromatic methyl group, 2′-Me (*δ*_H,C_ 2.36, 22.2), one methoxy function, 4′-OMe (*δ*_H,C_ 4.11, 57.0), and one isolated proton, H-7′ (*δ*_H_ 6.80, d, *J* = 1.18 Hz). HMBC correlations were monitored from H-1′ and H-3′ to 2′-Me (Fig. 2A), from H-3′ and 4′-OMe to C-4′ (*δ*_C_ 158.2), and from H-7′ to C-5′ (C-OH, *δ*_C_ 152.6), to C-9′ (*δ*_C_ 136.9), to C-5 (*δ*_C_ 119.9), and to C-6″ (*δ*_C_ 120.3). ROESY interactions of H-3′ with both, 2′-Me and 4′-OMe, confirmed the presence of a 6′,8′-substituted naphthalene subunit, which was in agreement with the HMBC, HSQC, COSY, and DEPT-135 data. Moreover, a substituted tetrahydroisoquinoline subportion was assigned according to a shielded aromatic proton at *δ*_H_ 6.62 (H-7), one aromatic methoxy group at *δ*_H_ 3.90 (8-OMe), two diastereotopic protons, 4-H_eq_ (*δ*_H_ 2.64, dd, *J* = 3.35, 17.78 Hz) and 4-H_ax_ (*δ*_H_ 2.27, dd, *J* = 12.14, 17.37 Hz), two methyl groups, 1-Me (*δ*_H_ 1.76, d, *J* = 6.70 Hz) and 3-Me (*δ*_H_ 1.24, d, *J* = 6.52 Hz), one quartet, H-1 (*δ*_H_ 4.65, q, *J* = 6.63 Hz), and one multiplet, H-3 (*δ*_H_ 3.28, m). The position of H-7 was confirmed by its HMBC cross peaks with C-6 (C-OH, *δ*_C_ 157.2), C-8 (C-OMe, *δ*_C_ 158.0), and C-1 (*δ*_C_ 52.0). HMBC correlations from H-7, H-7′, and 4-H_eq_ to C-5 (*δ*_C_ 119.9) proved that the two subunits of this monomeric half were 5,8′-coupled and, thus, linked *via* a rotationally hindered biaryl axis (see Fig. 2A).

**Table 1:** ^1^H (600 MHz) and ^13^C (151 MHz) data of ealapasamines A–C (**1**–**3**) in Methanol-d4 (J in Hz, *δ* in ppm).

Position	1		2		3	
	*δ*_H_ (*J* in Hz)	*δ*_C_, type	*δ*_H_ (*J* in Hz)	*δ*_C_, type	*δ*_H_ (*J* in Hz)	*δ*_C_, type
1	4.65, q (6.6)	52.0, CH	4.78, q (6.8)	49.4, CH	4.65, q (6.6)	52.1, CH
3	3.28, m	50.7, CH	3.70, m	45.1, CH	3.25, m	50.9, CH
4	2.64, dd (17.8, 3.6)	33.1, CH^eq^	2.83, dd (18.1, 4.6)	33.2, CH^eq^	2.62, dd (17.2, 3.4)	33.2, CH^eq^
	2.27, dd (17.4, 12.1)	33.1, CH^ax^	2.15, dd (18.1, 11.2)	33.2, CH^ax^	2.28, dd (17.8, 12.0)	33.2, CH^ax^
5		119.9, C		120.0, C		120.0, C
6		157.1, C		157.5, C		157.2, C
7	6.62, s	99.3, CH	6.59, s	98.8, CH	6.62, s	99.5, CH
8		158.5, C		157.8, C		158.6, C
9		114.1, C		114.2, C		114.3, C
10		135.3, C		133.5, C		135.5, C
1′	6.80, d (1.2)	119.1, CH	6.70, br s	119.1, CH	6.80, s	119.3, CH
2′		137.6, C		137.8, C		137.4, C
3′	6.87, d (1.2)	108.0, CH	6.86, br s	108.2, CH	6.87, d (1.3)	108.2, CH
4′		158.2, C		158.0, C		158.3, C
5′		152.6, C		154.8, C		152.7, C
6′		120.2, C		120.5, C		120.3, C
7′	7.28, s	134.7, CH	7.30, s	134.8, CH	7.27, s	134.9, CH
8′		123.7, C		123.8, C		123.8, C
9′		136.9, C		136.7, C		137.1, C
10′		115.3, C		115.4, C		115.4, C
Me-1	1.76, d (6.7)	20.2, Me	1.61, d (6.7)	18.7, Me	1.76, d (6.6)	20.4, Me
Me-3	1.24, d (6.5)	18.7, Me	1.23, d (6.5)	19.4, Me	1.24, d (6.5)	18.8, Me
Me-2′	2.36, s	22.2, Me	2.34, s	22.3, Me	2.36, s	22.3, Me
8-OMe	3.90, s	55.9, Me	3.92, s	56.2, Me	3.90, s	56.1, Me
4′-OMe	4.11, s	57.0, Me	4.10, s	57.1, Me	4.11, s	57.2, Me
1‴	4.74, q (6.8)	49.9, CH	4.69, q (6.7)	52.6, CH	4.68, q (6.6)	52.5, CH
3‴	3.86, m	45.1, CH	3.47, m	51.3, CH	3.47, m	51.3, CH
4‴	3.15, dd (17.6, 4.8)	34.5, CHeq	2.97, m	35.3, CHeq	2.97, m	35.3, CHeq
	2.88, dd (17.8, 11.7)	34.5, CHax	2.97, m	35.3, CHax	2.97, m	35.3, CHax
5‴	6.57, s	111.2, CH	6.60, s	111.9, CH	6.60, s	112.0, CH
6‴		157.6, C		157.6, C		157.5, C
7‴		121.0, C		121.9, C		121.9, C
8‴		157.5, C		158.7, C		158.7, C
9‴		118.5, C		118.5, C		118.5, C
10‴		132.9, C		134.9, C		134.9, C
1″	6.88, d (1.2)	119.8, CH	6.88, d (1.2)	120.1, CH	6.88, d (1.2)	120.1, CH
2″		137.3, C		137.3, C		137.8, C
3″	6.86, d (1.2)	107.9, CH	6.86, br s	108.0, CH	6.86, br s	108.1, CH
4″		157.9, C		158.4, C		158.0, C
5″		152.5, C		152.6, C		152.5, C
6″		120.3, C		120.3, C		120.2, C
7″	7.39, s	135.2, CH	7.39, s	135.1, CH	7.39, s	135.1, CH
8″		122.0, C		122.3, C		122.3 C
9″		136.4, C		136.6, C		136.6, C
10″		114.9, C		115.1, C		115.1, C
Me-1‴	1.63, d (6.9)	19.5, Me	1.74, d (6.9)	20.7, Me	1.73, d (6.9)	20.7, Me
Me-3‴	1.51, d (6.9)	19.3, Me	1.51, d (6.5)	19.0, Me	1.51, d (6.5)	18.9, Me
Me-2″	2.35, s	22.1, Me	2.37, s	22.6, Me	2.37, s	22.4, Me
8‴-OMe	3.19, s	60.9, Me	3.27, s	61.0, Me	3.27, s	61.0, Me
4″-OMe	4.11, s	57.0, Me	4.11, s	57.1, Me	4.11, s	57.2, Me

**Figure 3:**
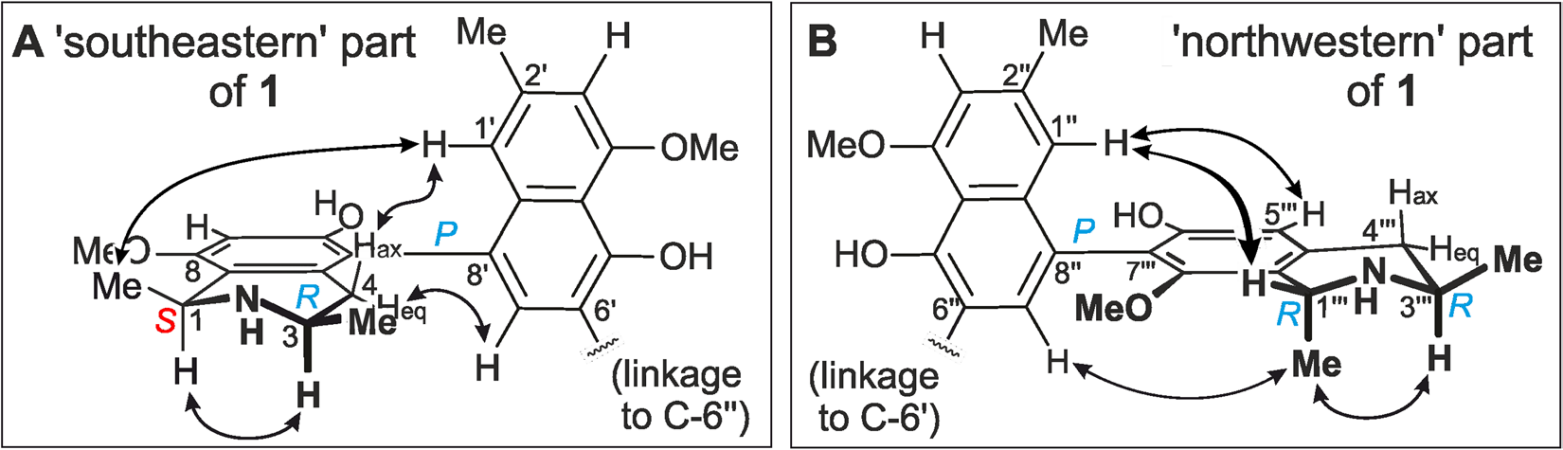
ROESY interactions defining the relative configurations at the stereogenic centers and axes within the monomeric halves of 1: (**A**) for the 5,8′-coupled part, and (**B**) for the 7,8′-linked portion.

The position of the methoxy substituent at C-8 was corroborated by its ROE correlations with H-7 and H-1 (Fig. 2A). The ROESY interactions between H-1 and H-3 supported the relative *cis*-configuration at the two stereogenic centers in the isoquinoline moiety. The ROESY correlations between 8-OMe and H-1, 1-Me, and H-7, and between 4′-OMe and H-3′, were in agreement with the proposed constitution for this southeastern half (Fig. 3A). The absolute configurations at the chiral centers C-3 and C-3‴ were determined to be *R* by ruthenium-mediated oxidative degradation^14^, providing (*R*)-3-aminobutyric acid. Due to the above-established relative *cis*-configuration at C-1 *versus* C-3, the absolute configuration at C-1 was deduced to be *S*. Based on the ROESY interactions from H-1′ to 4-H_ax_ and 1-Me, and from 4-H_eq_ to H-7′, the axial configuration was assigned to be *P* in this southeastern half (Fig. 3A).

The NMR data corresponding to the ‘northwestern’ half of the first dimer displayed one isolated singlet at *δ*_H_ 7.39 (s, H-7″, *δ*_C_ 135.2), two doublets with a *meta*-coupling pattern at *δ*_H_ 6.88 (d, *J* = 1.18 Hz, H-1″, *δ*_C_ 119.8) and 6.86 (d, *J* = 1.16 Hz, H-3″, *δ*_C_ 107.9), one methoxy group at *δ*_H_ 4.11 (s, 4″-OMe, *δ*_C_ 57.0), a methyl group at *δ*_H_ 2.35 (s, 2″-Me, *δ*_C_ 22.1), reminiscent of the naphthalene subunit in the other portion. These assignments were in agreement with the HSQC and HMBC data (Fig. 2B, see also Supplementary Table S2). 1D and 2D NMR data revealed further aromatic and heterocyclic spin systems, namely an aromatic singlet at *δ*_H_ 6.57 (s, H-5‴, *δ*_C_ 111.2), a quartet at 4.74 (q, *J* = 6.77 Hz, H-1‴, *δ*_C_ 49.9), a multiplet at *δ*_H_ 3.86 (m, H-3‴, *δ*_C_ 45.1), two doublets of doublets for the diastereotopic protons at C-4‴ at *δ*_H_ 3.15 (dd, *J* = 4.81, 17.58 Hz, 4‴-H_eq_, *δ*_C_ 34.5) and 2.88 (dd, *J* = 11.72, 17.78 Hz, 4‴-H_ax_, *δ*_C_ 34.5), one high-field shifted methoxy group at *δ*_H_ 3.19 (s, 8‴-OMe, *δ*_C_ 61.0), two methyl groups at *δ*_H_ 1.63 (d, *J* = 6.90 Hz, 1‴-Me, *δ*_C_ 19.5) and 1.51 (d, *J* = 6.87 Hz, 3‴-Me, *δ*_C_ 19.3). The HMBC interactions from H-5‴ to C-4‴, to C-7‴ (*δ*_C_ 121.0), and to C-6‴ (C-OH, *δ*_C_ 157.6), and from H-1‴ to C-8‴ (*δ*_C_ 157.5) suggested the presence of a tetrahydroisoquinoline subunit with no substituent at C-5‴ (Table 1). Moreover, the HMBC interactions from H-5‴ and H-7″ to C-7‴, and from H-7″ to C-9″ (*δ*_C_ 136.4) and C-5″ (*δ*_C_ 152.5) revealed the naphthalene and isoquinoline subunits of this second molecular half to be connected *via* a 7‴,8″-biaryl axis (Fig. 3B). This assignment was further proven by the ROESY correlations of 8‴-OMe with H-1‴ and 1‴-Me. In the ROESY spectrum, the cross peaks between H-3‴ and the protons of 1‴-Me established the relative configuration of the stereocenters at C-1”‘ and C-3‴ to be *trans* (Fig. 3B), in contrast to the observed *cis*-configured subunit in the southeastern molecular half. The oxidative degradation procedure again delivered only (*R*)-3-aminobutyric acid, thus the absolute configuration at C-3‴ was attributed to be *R*. 7,8′-linked naphthylisoquinolines are most challenging to be structurally assigned by NOEs and/or ECD, in particular when being part of a dimer^13^. A meticulous analysis of the ROEs showed the stereogenic center at C-1‴, with its spin systems H (above the isoquinoline plane) and Me (below), was spatially quite close to the axis, which permitted long-range ROE interactions across the axis over to the naphthalene part. Thus, ROESY correlations between H-7″ and the axial 1‴-Me (both below), and H-1″ with the equatorial H-1‴ (both above) unambiguously established the axis in the 7‴,8″-coupled northwestern half of the dimer to be *P*-configured.

Since the molecular moieties of this new ‘mixed’, unsymmetric quateraryl were coupled *via* C-6’ of both naphthalene portions, *i.e*., in the least-hindered positions, the central biaryl axis was not an additional element of chirality, but can freely rotate. The new dimer thus had the full absolute stereostructure **1**, as depicted in Fig. 1. In view of its occurrence in *A. ealaensis* and according to the Lingala word *pasa* (=twins), the new dimer **1** was named ealapasamine A.

**Figure 4:**
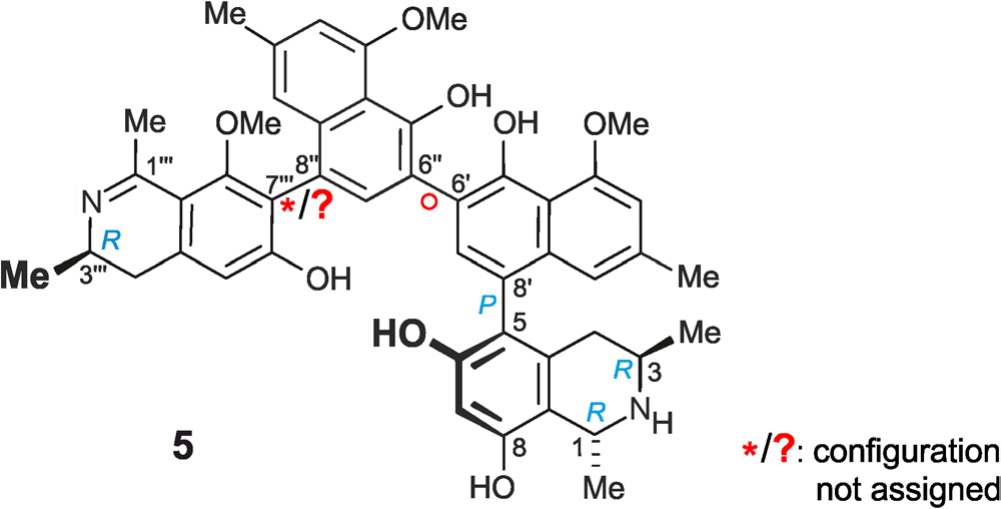
Korundamine A (**5**) previously isolated from *A. korupensis* ^15^.

Prior to this work, only one single alkaloid with a related molecular scaffold had been known, korundamine A (**5**)^15^ from the Cameroonian species *A. korupensis*. This dimer likewise consists of a 5,8′- and a 7,8′-coupled monomer, but its structural elucidation had remained incomplete. The assignment of the relative (and, thus, absolute) axial configuration of the 7,8′-linked molecular half had failed, because no decisive ROE relationships were monitored in its dihydroisoquinoline part (Fig. 4)^15^. Ealapasamine A (**1**) is, thus, the very first fully stereochemically elucidated ‘mixed’ heterodimer with two differently coupled naphthylisoquinoline portions that has been fully stereochemically assigned. This assignment was facilitated by the presence of an additional stereocenter at C-1‴, as compared to **5**, with its ‘flat’ imino function at C-1‴ and its occurrence in trace amounts only.

#### Ealapasamine B (2)

**Figure 5:**
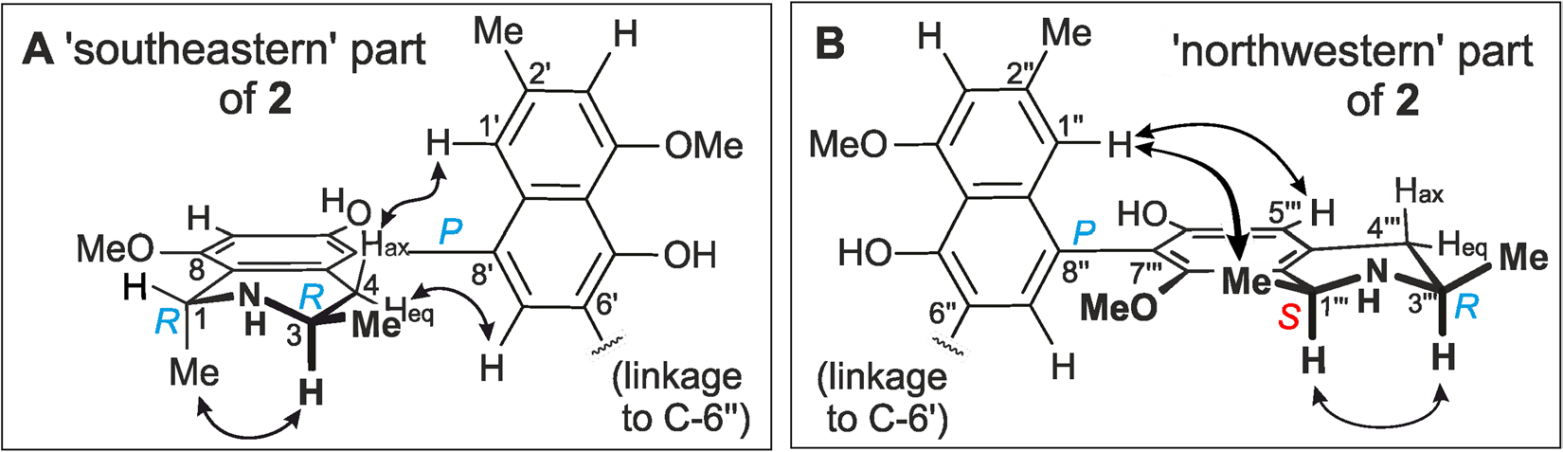
ROESY interactions indicative of the relative configurations at the stereogenic centers and axes within the northwestern (**A**) and southeastern (**B**) molecular halves of ealapasamine B (**2**).

From another dimer-enriched fraction of the leaves of *A. ealaensis*, a closely related second compound was isolated, albeit in very small quantities only. According to HRESIMS and NMR, this dimer had the same molecular formula as the above- described ealapasamine A (**1**), and the same constitution, and was, thus a new compound, too (Table 1, see also Supplementary Table S3 and Fig. S5). The relative configuration at C-1‴ *versus* C-3‴ in the ‘northwestern’ half (Fig. 5A) was deduced to be *cis* from a ROESY correlation between H-1‴ (*δ*_H_ 4.69) and H-3‴ (*δ*_H_ 3.47), while a ROESY interaction between 1-Me (*δ*_H_ 1.61) and H-3 (*δ*_H_ 3.70) revealed a relative *trans*-configuration at C-1 and C-3 in the isoquinoline portion of the ‘southeastern’ half of the molecule (Fig. 5B). In contrast to compound **1**, dimer **2** showed a slightly deshielded H-1 (*δ*_H_ 4.78) and a shielded C-3 (*δ*_C_ 45.1), and the chemical shifts of H-1‴ and C-3‴ (*δ*_C_ 51.3), thus likewise corroborating the assignment of the relative configurations in the two tetrahydroisoquinoline portions. The oxidative degradation^14^ delivered aminobutyric acid as its *R*-enantiomer only, which implied the absolute configurations of the molecular halves of **2** to be 1 *R*,3 *R* in the 5,8′-coupled monomer, and 1 *S*,3 *R* in the 7,8′-linked portion. Similar to **1**, the ROESY correlations between H-1′ and 4-H_ax_ in the southeastern half (Fig. 5B), and between 1‴-Me and H-1″ for the northwestern half (Fig. 5A) attributed a *P*-configuration to the two outer biaryl axes. Hence, this new dimer had the full absolute structure **2**. It was named ealapasamine B.

#### Ealapasamine C (3)

A third compound was isolated, along with ealapasamine B, again with a molecular formula identical to those of **1** and **2**. Despite some different NMR shifts, its constitution was the same as that of **1** and **2**, displaying (see Table 1) slightly shielded quartets at H-1 (*δ*_H_ 4.65) and H-1‴ (*δ*_H_ 4.68), and deshielded signals of C-3 (*δ*_C_ 50.9) and C-3‴ (*δ*_C_ 51.3). This hinted at relative 1,3-*cis*-configurations in both tetrahydroisoquinoline moieties, which was further confirmed by ROESY measurements (see Supplementary Fig. S6). Oxidative degradation^14^ determined the absolute configuration at both, C-3 and C-3‴ to be *R*, which, in combination with the relative *cis*-configurations of the two isoquinoline portions, established the stereocenters at C-1 and C-1‴ to be *S*-configured. Long-range ROESY cross-peaks from H-7′ with 4-H_eq_, and from H-1″ with 1‴-Me (see Fig. S5), like in the *cis*-configured moieties of **1** and **2**, assigned the two outer biaryl axes to be again *P*-configured. The new alkaloid had, thus, the structure **3** and was henceforth named ealapasamine C.

**Figure 6:**
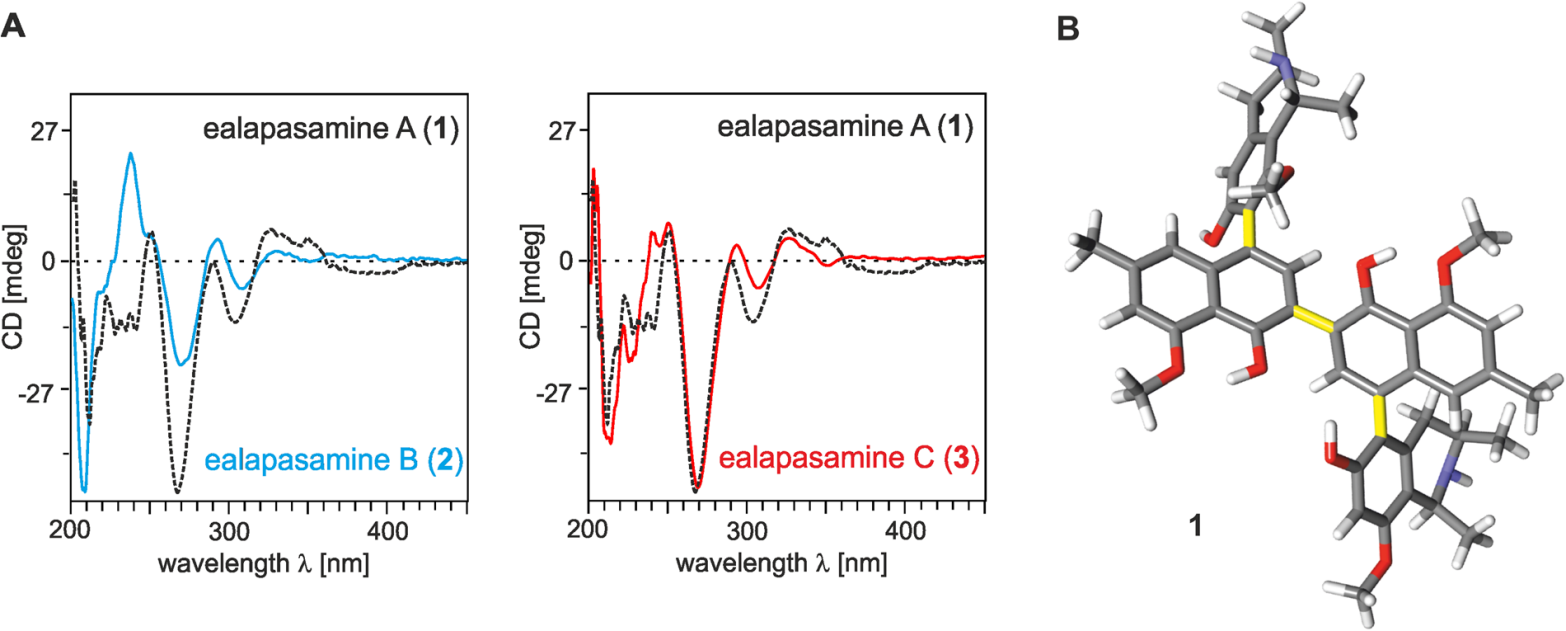
(**A**) ECD spectra of ealapasamines (A–C) (**1**–**3**) recorded in MeOH. (**B**) One DFT-optimized conformer of **1**.

Given the freely rotating central biaryl axes, the ECD spectra of **1**–**3** were all dominated by the chiroptical contributions of the outer axes^13^. Since these were identically configured for all the three new dimers, their ECD spectra were most similar to each other (Fig. 6A). DFT-based geometric optimizations were also performed for **1**-**3**, leading to the identification of most populated conformers (Fig. 6B, see also Supplementary information).

### Biological evaluations

The ealapasamines A–C (**1**–**3**) exhibited excellent *in vitro* antimalarial activities against chloroquine-sensitive (NF54) and chloroquine-resistant (K1) strains of the malaria parasite *Plasmodium falciparum* (Table 2), with IC_50_ values of 418 (NF54) and 452 nM (K1) for **1**, 210 (NF54) and 138 nM (K1) for **2**, and 34 (NF_54_) and 6.3 nM (K1) for **3**. Compound **3** is, thus, the as yet most active naphthylisoquinoline against the resistant strain K1. Its cytotoxicity was comparatively low (6.0 μM), giving a high selectivity index of nearly 1000. Against *Trypanosoma b. rhodesiense* and *Leishmania donovani*, by contrast, virtually no activities or very low ones were determined, which demonstrates the high specificity of the antiplasmodial activities of **1**–**3**, making advanced biological evaluations on the most active dimer, **3**, a rewarding task.

**Table 2:** Biological evaluations of 1–3 against *Plasmodium falciparum* (strains: NF54 and K1), *Trypanosoma brucei rhodesiense*, *T. cruzi*, and *Leishmania donovani*.

Compounds	*T. b. rhod*.	*T. cruzi*	*L*. *donovani* ax. am.	*P. falciparum* NF54/K1	L6 cells (cyto-toxicity)	Selectivity index to NF54/K1
Standard	0.007^[1]^	3.56^[2]^	0.43^[3]^	0.008^[4]^: NF54	0.041^[5]^	n.d.
				0.364^[4]^: K1		
**1**	16.33	74.04	>100	0.418	61.29	147
				0.452		136
**2**	5.45	—	>10	0.210	>12.73	>61
				0.138		>93
**3**	1.87	—	>10	0.034 (NF54)	5.98	174
				**0.006** (K1)		**997**
Jozimine A_2_^16^	—	—	—	0.001	15.9	11400
				0.016*		—

Also the cytotoxicities against rat skeletal myoblasts (L6 cells) were determined (IC_50_ in µM). The profile of the dimeric naphthylisoquinoline alkaloid jozimine A_2_^16^ is used for comparison of the antiplasmodial activities. ^[1]^Melarsoprol. ^[2]^Benznidazole. ^[3]^Miltefosine. ^[4]^Chloroquine. [5] Podophyllotoxin. All values in µM. n.d: not determined. Selectivity index determined for *P. falciparum*. *Yet unpublished value.

The ealapasamines are structurally unique in many respects: Among the small subfamily of dimeric naphthylisoquinoline alkaloids (presently ca. 20 compounds)^1, 5–9^, they are the only fully elucidated ‘mixed’ heterodimers with totally different coupling types at the three biaryl axes (5,8′-, 6′,6″-, and 7‴,8″-coupling). Besides the unprecedented occurrence of such unsymmetric dimers in *A. ealaensis* (and previously, but not fully assigned, in *A. korupensis* ^15^), these alkaloids display exciting antiplasmodial activities.

## Experimental Section

### General experimental procedures

A Jasco^®^ LC-2000Plus Series System (Gross-Umstadt, Germany) was used for the HPLC-DAD analyses. LC-MS measurements were performed on an Agilent 1100 Series System, equipped with a binary high-pressure mixing pump, with a degasser module, an autosampler, an 1100 series photodiode array (PDA) detector (Agilent Technology, Germany), and an Esquire 3000 Plus ion-trap mass spectrometer with an electrospray ionization interface (Bruker Daltonics, Bremen, Germany). A Bruker Daltonics microTOF spectrometer focus was used for the high-resolution electrospray mass spectrometry. NMR analyses were acquired on AMX 400 and DMX 600 Bruker spectrometers. The offline ECD and ORD spectra were obtained on a Jasco J-715 spectropolarimeter. The data were evaluated using SpecDis_164^17^. A Shimadzu UV-1800 spectrophotometer was used to perform in triplicate offline UV measurements. The Jasco P-1020-polarimeter operating with a sodium light source (λ = 589 nm) was used for the measurement of the optical rotation. The mechanical shaker operating at the frequency of 160 RPM (rotation per minutes) was from Bottmingen (Switzerland).

### Plant material

Leaf material of *Ancistrocladus ealaensis* ^11, 18^ was collected in the Botanical Garden of Eala (Mbandaka, Democratic Republic of the Congo), in August 2008 by one of us (V.M.) and in August 2015 by Mr. B.K. Lombe (GPS coordinates 00°03.605 N, 018°18.886E). The material of 2015 was authenticated additionally by LC-DAD-MS to contain the same metabolites as the one of 2008. Voucher specimens are available at the Herbarium Bringmann at the Institute of Organic Chemistry, University of Würzburg (no. 43 and 57).

### Extraction and isolation

The air-dried powder of the leaves (600 g) was macerated in MeOH, under mechanical shaking (160 RPM) for 24 h, after filtration the marc was further macerated until exhaustion. The 24h-macerates were mixed after filtration, and evaporated to a viscous solution. The methanolic extract was dissolved in water to permit the precipitation of chlorophyll. The aqueous layer was partitioned first with *n*-hexane, until the upper phase was cleared of residual chlorophyll, and then exhaustively extracted with CH_2_Cl_2_. The organic layer was evaporated to dryness to obtain the metabolites-rich fraction A. Fraction A was then subjected to preparative liquid chromatography using C_18_-reversed phase silica gel. The mobile system consisted of acetonitrile (MeCN) and ultrapure water containing 0.05% TFA (trifluoroacetic acid). The elution was performed from 0 to 50 per cent H_2_O in MeCN to afford 100 fractions. Further fractionation of fractions A_77_ to A_88_ on five C18-SPE cartridges in series (Sep-Pak C_18_ Plus Light Cartridge, 130 mg, 55–105 µm) using the same eluting conditions led to several sub-fractions enriched with dimeric-alkaloids which were submitted to semi-preparative HPLC to isolate the dimers.

### Semi-preparative HPLC conditions

The isolation from alkaloid-enriched fractions was performed on a SymmetryPrep-C_18_ column (Waters, 300 × 19 mm, 7 μm) with the mobile phase consisting of A (H_2_O, 0.05% TFA), B (MeCN, 0.05% TFA), at a flow rate of 10 mL/min. Further purification on a Chromolith SemiPrep RP-18e column (100 × 10 mm) afforded the pure dimeric alkaloids, using a gradient system similar to the one described above on the SymmetryPrep column, but with MeCN replaced by MeOH (C), at the same flow rate.

The fractionation on reversed phase silica gel was guided by LC-MS searching for masses hinting at the presence of dimeric alkaloids. The compounds of interest were found to be distributed unequally between the fractions A_77_ to A_88_. For the isolation of **1**–**3**, the fractions of interest were submitted to semi-preparative HPLC on a SymmetryPrep-C18 column using a linear gradient at a flow rate of 10 mL/min: 0–13 min: 10–20% of B, 37 min: 45% of B, 39 min: 50% of B, 42 min: 100% of B, 45 min: 100% of B. Some of the collected peaks (still impure, but containing **1–3**) required additional purification steps, which were performed by HPLC on a Chromolith SemiPrep RP-18e column (100 × 10 mm) using a gradient solvent system consisting of A (H_2_O, 0.05% TFA) and C (MeOH, 0.05% TFA) at a flow rate of 10 mL/min: 0–2 min: 10% of C, 8 min: 30% of C, 11 min: 30% of C, 11.2 min: 35% of C, 16 min: 35% of C, 20 min: 40% of C, 25 min: 45% of C, 27 min: 100% of C, 30 min: 100%, to yield 5 mg of ealapasamine A (**1**), 1.5 mg of ealapasamine B (**2**), and 3.5 mg of ealapasamine C (**3**).

#### Ealapasamine A (**1**)

White amorphous powder; ${[{\rm{\alpha }}]}_{{D}}^{23}-21$ (*c* = 0.09, MeOH); UV (MeOH) λ_max_ (log ε) = 205 (1.32), 217 (0.91), 230 (1.09), 257 (0.54), 262 (0.54), 297 (0.24), 315 (0.28), 322 (0.28), 329 (0.29), 338 (0.28), 344 (0.29) nm; ECD (MeOH, *c* 0.02) λ_max_ (log ε in cm^2^ mol^−1^) 195 (+10.1), 200 (+2.83), 210 (‒7.81), 221 (‒1.71), 227 (‒3.51), 234 (−3.34), 240 (−3.32), 250 (+1.36), 267 (−11.01), 290 (−0.09), 303 (−2.96), 351 (+1.1), 395 (−0.6) nm; ORD (MeOH, *c* 0.02) λ_max_ (log ε in cm^2^ mol^−1^) 200 (+5.3), 204 (+9.0), 207 (+7.7), 219 (−0.4), 226 (+1.8), 238 (+0.6), 244 (−1.4), 259 (+6.7), 279 (−6.6), 298 (−1.4), 317 (−4.7), 348 (−1.2), 363.8 (0.0), 380 (−0.5), 404 (−1.0) nm; ^1^H NMR and ^13^C NMR data: see Table 1; HRESIMS *m/z* 785.37804 [M + H]^+^ (calcd for C_48_H_53_N_2_O_8_, 785.37964).

#### Ealapasamine B (**2**)

White amorphous powder; ${[{\rm{\alpha }}]}_{D}^{23}-10$ (*c* 0.04, MeOH); UV (MeOH) λ_max_ (log ε) 205 (1.2), 219 (0.81), 228 (0.85), 257 (0.42), 267 (0.45), 305 (0.16), 312 (0.17), 323 (0.16), 330 (0.16), 338 (0.16), 344 (0.16) nm; ECD (MeOH, *c* 0.005) λ_max_ (log ε in cm^2^ mol^−1^) 195 (+1.3), 200 (−1.26), 207 (−7.68), 236 (+3.57), 240 (+2.43), 267 (−3.5), 290 (+0.7), 304 (−0.94), 334 (−0.23), 386 (+0.17) nm; ORD (MeOH, *c* 0.005) λ_max_ (log ε in cm^2^ mol^−1^) 200 (+4.1), 202 (+5.0), 212 (−4.4), 221 (−2.8), 228 (−3.3), 242 (+1.8), 246 (+1.4), 258 (+2.6), 280 (−2.6), 297 (+0.1), 315 (−1.3), 355 (−0.6), 365 (−0.7), 400 (−0.3) nm; ^1^H NMR and ^13^C NMR data: see Table 1; HRESIMS *m/z* 785.38042 [M + H]^+^ (calcd for C_48_H_53_N_2_O_8_, 785.37964).

#### Ealapasamine C (**3**)

White amorphous powder; ${[{\rm{\alpha }}]}_{D}^{23}-26$ (*c* 0.1, MeOH); UV (MeOH) λ_max_ (log ε) 217 (0.83), 229 (0.93), 257 (0.37), 263 (0.4), 300 (0.14), 315 (0.16), 322 (0.15), 330 (0.16), 338 (0.16), 344 (0.17) nm; ECD (MeOH, *c* 0.01) λ_max_ (log ε in cm^2^ mol^−1^) 195 (+7.34), 200 (−1.66), 202 (+5.2), 212 (−11.1), 224 (−6.23), 249 (+1.95), 267 (−13.66), 292 (+0.65), 305 (−1.89), 325 (+1.1), 349 (−0.56), 385 (−0.17) nm; ORD (MeOH, *c* 0.01) λ_max_ (log ε in cm^2^ mol^−1^) 200 (−0.9), 206 (+13.2), 218 (−2.6), 222 (+0.1), 231 (−4.4), 242 (+0.0), 259 (+8.4), 280 (−8.5), 299 (−2.3), 315 (−4.2), 343 (−1.0), 354 (−1.7), 400 (−1.1) nm; ^1^H NMR and ^13^C NMR data: see Table 1; HRESIMS *m/z* 785.37932 [M + H]^+^ (calcd for C_48_H_53_N_2_O_8_, 785.37964).

### Oxidative degradation

The ruthenium (VIII)-mediated periodate degradation of the ealapasamines A–C (**1**–**3**), the Mosher-type derivatization of the resulting amino acids using MeOH/HCl and *R*-α-methoxy-α-trifluoromethylacetyl chloride (*R*-MTPA-Cl, prepared from *S*-MTPA), and the subsequent GC-MSD analysis were carried out as described earlier^14^.

### Computational analysis

The DFT structural geometry optimizations of ealapasamines A-C were calculated using the B3LYP-D3/def2-TZVP method, with ORCA^19–21^.

### Biological evaluation

Antiprotozoal *in vitro* tests were performed on the NF54 (chloroquine-sensitive) and K1 (chloroquine- and pyrimethamine-resistant) strains of *Plasmodium falciparum*, STIB 900 strain of *Trypanosoma brucei rhodesiense* (trypomastigotes), Tulahuen C4 strain of *Trypanosoma cruzi* (amastigotes), and MHOM-ET-67/L82 strain *Leishmania donovani* (amastigotes). The cytotoxicity on mammalian host cells (rat skeletal myoblast L6 cells) were determined according to established protocols^22^.

## Electronic supplementary material

Supplementary Information
