# Supplementary material for: Antiplasmodial Ealapasamines A-C,‘Mixed’ Naphthylisoquinoline Dimers from the Central African Liana Ancistrocladus ealaensis
Source: Sci Rep. 2017 Jul 18;7:5767. doi: 10.1038/s41598-017-05719-w (PMC5515985; doi:10.1038/s41598-017-05719-w)
Supplement: Supplementary file 1 — Supplementary Information [file 41598_2017_5719_MOESM1_ESM.pdf]

Supplementary Information (SI)

Antiplasmodial Ealapasamines A-C, 'Mixed' Naphthylisoquinoline Dimers from the Central African Liana *Ancistrocladus ealaensis*

Dieudonné Tshitenge Tshitenge[1,2], Doris Feineis[1], Virima Mudogo[3], Marcel Kaiser[4,5], Reto Brun[4,5], and Gerhard Bringmann[1,*]

[1]Institute of Organic Chemistry, University of Würzburg, Am Hubland, D-97074 Würzburg, Germany

[2]Faculty of Pharmaceutical Sciences, University of Kinshasa, B.P. 212 Kinshasa XI, Democratic Republic of the Congo

[3]Faculty of Sciences, University of Kinshasa, B.P. 202 Kinshasa XI, Democratic Republic of the Congo

[4]Swiss Tropical and Public Health Institute, Socinstrasse 57, CH-4002 Basel, Switzerland

[5]University of Basel, Petersplatz 1, CH-4003 Basel, Switzerland

*To whom correspondence and requests for materials should be addressed (GB): Fax: +49-931-3184755; Tel: +49-931-3185323. Email: bringman@chemie.uni-wuerzburg.de

Table of Contents

Tables

Table S1. ^{1}H (600 MHz) and ^{13}C (150 MHz) data of ealapasamine A-C (**1-3**) 4
Table S2. ^{1}H, ^{13}C, HMBC, and ROESY NMR spectral data of ealapasamine A (**1**) 6
Table S3. ^{1}H, ^{13}C, HMBC, and ROESY NMR spectral data of ealapasamine B (**2**) 8
Table S4. ^{1}H, ^{13}C, HMBC, and ROESY NMR spectral data of ealapasamine C (**3**) 10

Figures

Figure S5. NMR Assignments of ealapasamine A-C (**1-3**) 12
Figure S6. ROESY interactions and axial configuration in **3** 13
Figures S7a-c. ^{1}H NMR spectra of ealapasamine A (**1**) in methanol-d_4..................................14
Figures S8a-e. ^{13}C NMR spectra of compound **1** in methanol-d_4..16
Figures S9a-c. DEPT NMR spectra of compound **1** in methanol-d_4 19
Figures S10a-c. HSQC spectra of compound **1** in methanol-d_4 20
Figure S11. HMBC spectra of compound **1** in methanol-d_4 21
Figure S12. COSY spectra of compound **1** in methanol-d_4 22
Figures S13a-c. ROESY spectra of compound **1** in methanol-d_4 23
Figure S14. HRESIMS spectrum of compound **1** in methanol 26
Figure S15. ECD spectrum of compound **1** in methanol 27
Figure S16. ORD-E spectrum of compound **1** in methanol 27
Figure S17. Offline UV spectrum of compound **1** in methanol 27
Figure S18. Oxidative degradation results of compound **1** 28
Figure S19. DFT-structural optimization for compound **1** 29
Figures S20a-d. ^{1}H NMR spectra of ealapasamine B (**2**) in methanol-d_4 30
Figures S21a-c. ^{13}C NMR spectra of compound **2** in methanol-d_4 32
Figure S22. DEPT NMR spectra of compound **2** in methanol-d_4 34
Figures S23a,b. HSQC spectra of compound **2** in methanol-d_4 35
Figure S24. HMBC spectra of compound **2** in methanol-d_4 37
Figure S25. COSY spectra of compound **2** in methanol-d_4 38
Figures S26a-d. ROESY spectra of compound **2** in methanol-d_4 39
Figure S27. HRESIMS spectrum of compound **2** in methanol 41
Figure S28. ECD spectrum of compound **2** in methanol 42
Figure S29. ORD-E spectrum of compound **2** in methanol 42

Figure S30. Offline UV spectrum of compound **2** in methanol 42
Figure S31. Oxidative degradation results of compound **2** 43
Figure S32. DFT-structural optimization for compound **2** 44
Figures S33a-d. ^{1}H NMR spectra of ealapasamine C **(3)** in methanol-d_4 45
Figures S34a,e. ^{13}C NMR spectra of compound **3** in methanol-d_4 47
Figures S35a-c. DEPT NMR spectra of compound **3** in methanol-d_4 50
Figures S36a,b. HSQC spectra of compound **3** in methanol-d_4 51
Figure S37. HMBC spectra of compound **3** in methanol-d_4 53
Figure S38. COSY spectra of compound **3** in methanol-d_4 54
Figures S39a-d. ROESY spectra of compound **3** in methanol-d_4 55
Figure S40. HRESIMS spectrum of compound **3** in methanol 57
Figure S41. ECD spectrum of compound **3** in methanol 58
Figure S42. ORD-E spectrum of compound **3** in methanol 58
Figure S43. Offline UV spectrum of compound **3** in methanol 58
Figure S44. Oxidative degradation products of compound **3** 59
Figure S45. DFT-structural optimization for compound **3** 60

Table S1. ^{1}H (600 MHz) and ^{13}C (151 MHz) Data of ealapasamines **A-C** in methanol-d_4 (δ in ppm, J in Hz).

	ealapasamine A (**1**)		ealapasamine B (**2**)		ealapasamine C (**3**)	
Position	δ_H (J in Hz)	δ_C, type	δ_H (J in Hz)	δ_C, type	δ_H (J in Hz)	δ_C, type
1	4.65, q (6.6)	52.0, CH	4.78, q (6.8)	49.4, CH	4.65, q (6.6)	52.1, CH
3	3.28, m	50.7, CH	3.70, m	45.1, CH	3.25, m	50.9, CH
4	2.64, dd (17.8, 3.6)	33.1, CHeq	2.83, dd (18.1, 4.6)	33.2, CHeq	2.62, dd (17.2, 3.4)	33.2, CHeq
	2.27, dd (17.4, 12.1)	33.1, CHax	2.15, dd (18.1, 11.2)	33.2, CHax	2.28, dd (17.8, 12.0)	33.2, CHax
5		119.9, C		120.0, C		120.0, C
6		157.1, C		157.5, C		157.2, C
7	6.62, s	99.3, CH	6.59, s	98.8, CH	6.62, s	99.5, CH
8		158.5, C		157.8, C		158.6, C
9		114.1, C		114.2, C		114.3, C
10		135.3, C		133.5, C		135.5, C
1'	6.80, d (1.2)	119.1, CH	6.70, br s	119.1, CH	6.80, s	119.3, CH
2'		137.6, C		137.8, C		137.4, C
3'	6.87, d (1.2)	108.0, CH	6.86, br s	108.2, CH	6.87, d (1.3)	108.2, CH
4'		158.2, C		158.0, C		158.3, C
5'		152.6, C		154.8, C		152.7, C
6'		120.2, C		120.5, C		120.3, C
7'	7.28, s	134.7, CH	7.30, s	134.8, CH	7.27, s	134.9, CH
8'		123.7, C		123.8, C		123.8, C
9'		136.9, C		136.7, C		137.1, C
10'		115.3, C		115.4, C		115.4, C
CH_3-1	1.76, d (6.7)	20.2, CH_3	1.61, d (6.7)	18.7, CH_3	1.76, d (6.6)	20.4, CH_3
CH_3-3	1.24, d (6.5)	18.7, CH_3	1.23, d (6.5)	19.4, CH_3	1.24, d (6.5)	18.8, CH_3
CH_3-2'	2.36, s	22.2, CH_3	2.34, s	22.3, CH_3	2.36, s	22.3, CH_3
8-OCH_3	3.90, s	55.9, CH_3	3.92, s	56.2, CH_3	3.90, s	56.1, CH_3
4'-OCH_3	4.11, s	57.0, CH_3	4.10, s	57.1, CH_3	4.11, s	57.2, CH_3

Continuation of Table S1

1'''	4.74, q (6.8)	49.9, CH	4.69, q (6.7)	52.6, CH	4.68, q (6.6)	52.5, CH
3'''	3.86, m	45.1, CH	3.47, m	51.3, CH	3.47, m	51.3, CH
4'''	3.15, dd (17.6, 4.8)	34.5, CHeq	2.97, m	35.3, CHeq	2.97, m	35.3, CHeq
	2.88, dd (17.8, 11.7)	34.5, CHax	2.97, m	35.3, CHax	2.97, m	35.3, CHax
5'''	6.57, s	111.2, CH	6.60, s	111.9, CH	6.60, s	112.0, CH
6'''		157.6, C		157.6, C		157.5, C
7'''		121.0, C		121.9, C		121.9, C
8'''		157.5, C		158.7, C		158.7, C
9'''		118.5, C		118.5, C		118.5, C
10'''		132.9, C		134.9, C		134.9, C
1''	6.88, d (1.2)	119.8, CH	6.88, d (1.2)	120.1, CH	6.88, d (1.2)	120.1, CH
2''		137.3, C		137.3, C		137.8, C
3''	6.86, d (1.2)	107.9, CH	6.86, br s	108.0, CH	6.86, br s	108.1, CH
4''		157.9, C		158.4, C		158.0, C
5''		152.5, C		152.6, C		152.5, C
6''		120.3, C		120.3, C		120.2, C
7''	7.39, s	135.2, CH	7.39, s	135.1, CH	7.39, s	135.1, CH
8''		122.0, C		122.3, C		122.3 C
9''		136.4, C		136.6, C		136.6, C
10''		114.9, C		115.1, C		115.1, C
CH_3-1'''	1.63, d (6.9)	19.5, CH_3	1.74, d (6.9)	20.7, CH_3	1.73, d (6.9)	20.7, CH_3
CH_3-3'''	1.51, d (6.9)	19.3, CH_3	1.51, d (6.5)	19.0, CH_3	1.51, d (6.5)	18.9, CH_3
CH_3-2''	2.35, s	22.1, CH_3	2.37, s	22.6, CH_3	2.37, s	22.4, CH_3
8'''-OCH_3	3.19, s	60.9, CH_3	3.27, s	61.0, CH_3	3.27, s	61.0, CH_3
4''-OCH_3	4.11, s	57.0, CH_3	4.11, s	57.1, CH_3	4.11, s	57.2, CH_3

Table S2. Detailed NMR data of ealapasamine A (**1**) in methanol-d_4 (δ in ppm, J in Hz).

ealapasamine A (**1**)					
Position.	δ_H (J in Hz)	HSQC, DEPT	HMBC	COSY	ROESY
1	4.65, q (6.6)	52.0, CH	8, 9, 10, 1-CH_3	1-CH_3	3, 8-OCH_3
3	3.28, m	50.7, CH	4, 3-CH_3	4eq, 3-CH_3	1
4	2.64, dd (17.8, 3.6)	33.1, CHeq	5, 9, 10	3, 4ax	7', 3-CH_3
	2.27, dd (17.4, 12.1)	33.1, CHax	3, 9, 10, 3-CH_3	3, 4eq	1', 3-CH_3
5		119.9, C			
6		157.1, C			
7	6.62, s	99.3, CH	1, 5, 6, 8, 9, 8'		8-OCH_3
8		158.5, C			
9		114.1, C			
10		135.3, C			
1'	6.80, d (1.2)	119.1, CH	2', 3', 10', 8', 9'		4ax, 1-CH_3, 2'-CH_3
2'		137.6, C			
3'	6.87, d (1.2)	108.0, CH	1', 2', 4', 10'		2'-CH_3, 4'-OCH_3
4'		158.2, C			
5'		152.6, C			
6'		120.2, C			
7'	7.28, s	134.7, CH	5, 5', 9', 10', 6''		4eq, 7, 7''
8'		123.7, C			
9'		136.9, C			
10'		115.3, C			
1-CH_3	1.76, d (6.7)	20.2, CH_3	1, 9	1	8-OCH_3
3-CH_3	1.24, d (6.5)	18.7, CH_3	3, 4	3	4eq, 4ax
2'-CH_3	2.36, s	22.2, CH_3	1', 2', 3'		1', 3'
8-OCH_3	3.90, s	55.9, CH_3	8		7, 1-CH_3
4'-OCH_3	4.11, s	57.0, CH_3	4'		3'

Continuation of Table S2

1'''	4.74, q (6.8)	49.9, CH	3''', 8''', 9''', 10''', 1'''-CH_3	1'''-CH_3	1'', 8'''-OCH_3
3'''	3.86, m	45.1, CH	4''', 3'''-CH_3	4'''eq, 3'''-CH_3	1'''-CH_3
4'''	3.15, dd (17.6, 4.8)	34.5, CHeq	5''', 9''', 10'''	4ax, 3'''	5''', 3'''-CH_3
	2.88, dd (17.8, 11.7)	34.5, CHax	3, 9, 10, 3'''-CH_3	4eq, 3'''	3'''-CH_3
5'''	6.57, s	111.2, CH	4''', 6''', 7''', 9'''		4eq, 1''
6'''		157.6, C			
7'''		121.0, C			
8'''		157.5, C			
9'''		118.5, C			
10'''		132.9, C			
1''	6.88, d (1.2)	119.8, CH	2'', 3'', 10'', 8'', 9''		2''-CH_3, 1''', 5''', 8'''-OCH_3
2''		137.3, C			
3''	6.86, d (1.2)	107.9, CH	1'', 2'', 4'', 10''		2''-CH_3, 4''-OCH_3
4''		157.9, C			
5''		152.5, C			
6''		120.3, C			
7''	7.39, s	135.2, CH	6', 5'', 9'', 10'', 7'''		7', 1'''-CH_3, 8'''-OCH_3
8''		122.0, C			
9''		136.4, C			
10''		114.9, C			
CH_3-1'''	1.63, d (6.9)	19.5, CH_3	1''', 9'''	1'''	7'', 3''', 8'''-OCH_3
CH_3-3'''	1.51, d (6.9)	19.3, CH_3	3''', 4'''	3'''	4'''eq, 4'''ax
CH_3-2''	2.35, s	22.1, CH_3	1'', 2'', 3''		1'', 3''
8'''-OCH_3	3.19, s	60.9, CH_3	8'''		1'', 7'', 1'''
4''-OCH_3	4.11, s	57.0, CH_3	4''		3''

Table S3. Detailed NMR data of ealapasamine B (**2**) in methanol-d_4 (δ in ppm, J in Hz).

ealapasamine B (**2**)					
Position.	δ_H (J in Hz)	HSQC, DEPT	HMBC	COSY	ROESY
1	4.78, q (6.8)	49.4, CH	8, 9, 10, 1-CH_3	1-CH_3	8-OCH_3
3	3.70, m	45.1, CH	4, 3-CH_3	4eq, 3-CH_3	1-CH_3
4	2.83, dd (18.1, 4.6)	33.2, CHeq	5, 9, 10	3, 4ax	7', 3-CH_3
	2.15, dd (18.1, 11.2)	33.2, CHax	3, 9, 10, 3-CH_3	3, 4eq	1', 3-CH_3
5		120.0, C			
6		157.5, C			
7	6.59, s	98.8, CH	1, 5, 6, 8, 9, 8'		8-OCH_3
8		157.8, C			
9		114.2, C			
10		133.5, C			
1'	6.70, br s	119.1, CH	2', 3', 10', 8', 9'		4ax, 2'-CH_3
2'		137.8, C			
3'	6.86, br s	108.2, CH	1', 2', 4', 10'		2'-CH_3, 4'-OCH_3
4'		158.0, C			
5'		154.8, C			
6'		120.5, C			
7'	7.30, s	134.8, CH	5, 5', 9', 10', 6''		4eq, 7, 7''
8'		123.8, C			
9'		136.7, C			
10'		115.4, C			
1-CH_3	1.61, d (6.7)	18.7, CH_3	1, 9	1	8-OCH_3
3-CH_3	1.23, d (6.5)	19.4, CH_3	3, 4	3	4eq, 4ax
2'-CH_3	2.34, s	22.3, CH_3	1', 2', 3'		1', 3'
8-OCH_3	3.92, s	56.2, CH_3	8		7, 1-CH_3
4'-OCH_3	4.10, s	57.1, CH_3	4'		3'

Continuation of Table S3

1'''	4.69, q (6.7)	52.6, CH	3''', 8''', 9''', 10''', 1'''-CH_3	1'''-CH_3	3''', 8'''-OCH_3
3'''	3.47, m	51.3, CH	4''', 3'''-CH_3	4'''eq, 3'''-CH_3	1'''
4'''	2.97, m	35.3, CHeq	5''', 9''', 10'''	4ax, 3'''	5''', 3'''-CH_3
	2.97, m	35.3, CHax	3, 9, 10, 3'''-CH_3	4eq, 3'''	5''', 3'''-CH_3
5'''	6.60, s	111.9, CH	4''', 6''', 7''', 9'''		4eq, 1"
6'''		157.6, C			
7'''		121.9, C			
8'''		158.7, C			
9'''		118.5, C			
10'''		134.9, C			
1"	6.88, d (1.2)	120.1, CH	2", 3", 10", 8", 9"		2"-CH_3, 5''', 1'''-CH_3, 8'''-OCH_3
2"		137.3, C			
3"	6.86, br s	108.0, CH	1", 2", 4", 10"		2"-CH_3, 4"-OCH_3
4"		158.4, C			
5"		152.6, C			
6"		120.3, C			
7"	7.39, s	135.1, CH	6', 5", 9", 10", 7'''		7', 8'''-OCH_3
8"		122.3, C			
9"		136.6, C			
10"		115.1, C			
CH_3-1'''	1.74, d (6.9)	20.7, CH_3	1''', 9'''	1'''	1", 8'''-OCH_3
CH_3-3'''	1.51, d (6.5)	19.0, CH_3	3''', 4'''	3'''	4'''eq, 4'''ax
CH_3-2"	2.37, s	22.6, CH_3	1", 2", 3"		1", 3"
8'''-OCH_3	3.27, s	61.0, CH_3	8'''		1", 7", 1'''
4"-OCH_3	4.11, s	57.1, CH_3	4"		3"

Table S4. Detailed NMR data of ealapasamine C (**3**) in methanol-d_4 (δ in ppm, J in Hz).

ealapasamine C (**3**)					
Position.	δ_H (J in Hz)	HSQC, DEPT	HMBC	COSY	ROESY
1	4.65, q (6.6)	52.1, CH	8, 9, 10, 1-CH_3	1-CH_3	3, 8-OCH_3
3	3.25, m	50.9, CH	4, 3-CH_3	4eq, 3-CH_3	1
4	2.62, dd (17.2, 3.4)	33.2, CHeq	5, 9, 10	3, 4ax	7', 3-CH_3
	2.28, dd (17.8, 12.0)	33.2, CHax	3, 9, 10, 3-CH_3	3, 4eq	1', 3-CH_3
5		120.0, C			
6		157.2, C			
7	6.62, s	99.5, CH	1, 5, 6, 8, 9, 8'		8-OCH_3
8		158.6, C			
9		114.3, C			
10		135.5, C			
1'	6.80, s	119.3, CH	2', 3', 10', 8', 9'		4ax, 1-CH_3, 2'-CH_3
2'		137.4, C			
3'	6.87, d (1.3)	108.2, CH	1', 2', 4', 10'		2'-CH_3, 4'-OCH_3
4'		158.3, C			
5'		152.7, C			
6'		120.3, C			
7'	7.27, s	134.9, CH	5, 5', 9', 10', 6''		4eq, 7, 7''
8'		123.8, C			
9'		137.1, C			
10'		115.4, C			
1-CH_3	1.76, d (6.6)	20.4, CH_3	1, 9	1	8-OCH_3
3-CH_3	1.24, d (6.5)	18.8, CH_3	3, 4	3	4eq, 4ax
2'-CH_3	2.36, s	22.3, CH_3	1', 2', 3'		1', 3'
8-OCH_3	3.90, s	56.1, CH_3	8		7, 1-CH_3
4'-OCH_3	4.11, s	57.2, CH_3	4'		3'

Continuation of Table 4

1'''	4.68, q (6.6)	52.5, CH	3''', 8''', 9''', 10''', 1'''-CH_3	1'''-CH_3	3''', 8'''-OCH_3
3'''	3.47, m	51.3, CH	4''', 3'''-CH_3	4'''eq, 3'''-CH_3	1'''
4'''	2.97, m	35.3, CHeq	5''', 9''', 10'''	4ax, 3'''	5''', 3'''-CH_3
	2.97, m	35.3, CHax	3, 9, 10, 3'''-CH_3	4eq, 3'''	5''', 3'''-CH_3
5'''	6.60, s	112.0, CH	4''', 6''', 7''', 9'''		4eq, 1''
6'''		157.5, C			
7'''		121.9, C			
8'''		158.7, C			
9'''		118.5, C			
10'''		134.9, C			
1''	6.88, d (1.2)	120.1, CH	2'', 3'', 10'', 8'', 9''		2''-CH_3, 5''', 1'''-CH_3, 8'''-OCH_3
2''		137.8, C			
3''	6.86, br s	108.1, CH	1'', 2'', 4'', 10''		2''-CH_3, 4''-OCH_3
4''		158.0, C			
5''		152.5, C			
6''		120.2, C			
7''	7.39, s	135.1, CH	6', 5'', 9'', 10'', 7'''		7', 8'''-OCH_3
8''		122.3 C			
9''		136.6, C			
10''		115.1, C			
CH_3-1'''	1.73, d (6.9)	20.7, CH_3	1''', 9'''	1'''	1'', 8'''-OCH_3
CH_3-3'''	1.51, d (6.5)	18.9, CH_3	3''', 4'''	3'''	4'''eq, 4'''ax
CH_3-2''	2.37, s	22.4, CH_3	1'', 2'', 3''		1'', 3''
8'''-OCH_3	3.27, s	61.0, CH_3	8'''		1'', 7'', 1'''
4''-OCH_3	4.11, s	57.2, CH_3	4''		3''

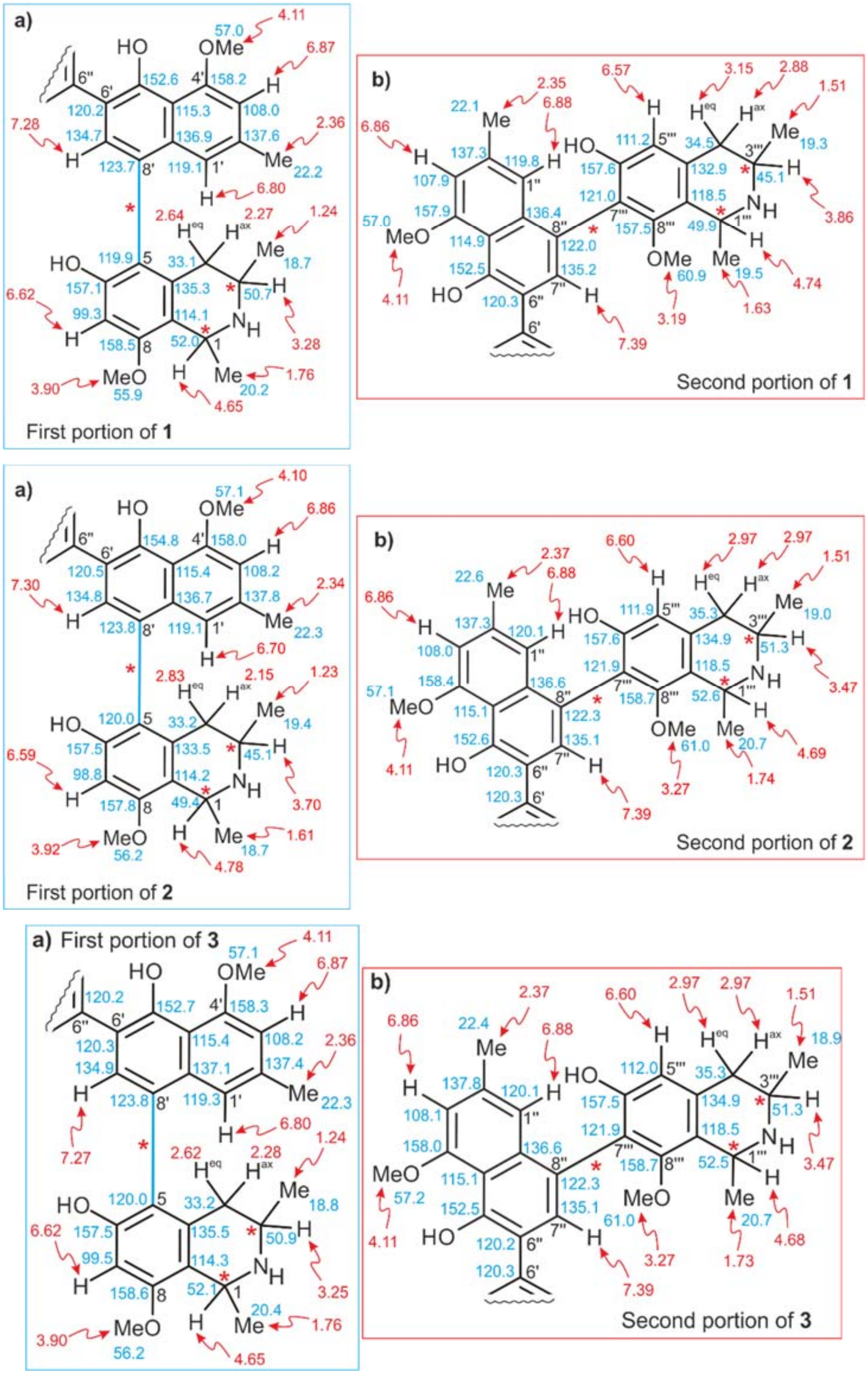

Figure S5. NMR Assignments of ealapasamine A-C (**1-3**).

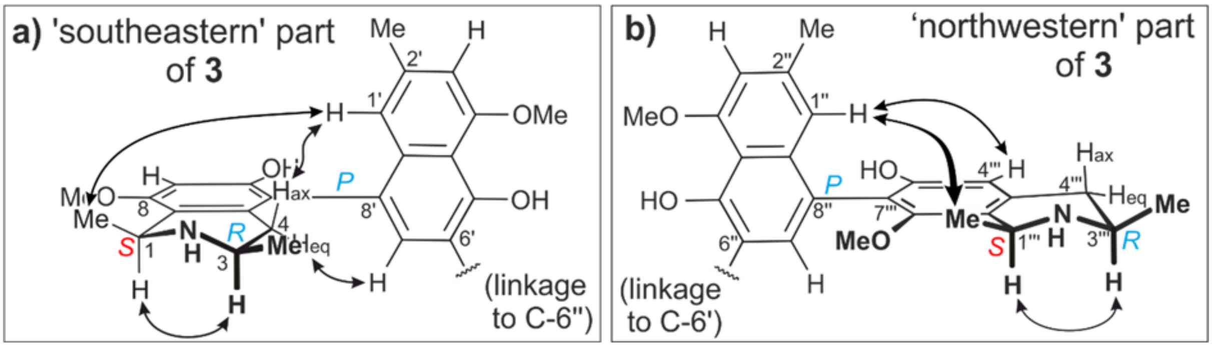

Figure S6. ROESY interactions and axial configuration in **3**: correlations defining the relative configurations at the stereogenic centers and axes within the monomeric halves of **3**: (a) for the 5,8'-coupled part, and (b) for the 7,8'-linked portion.

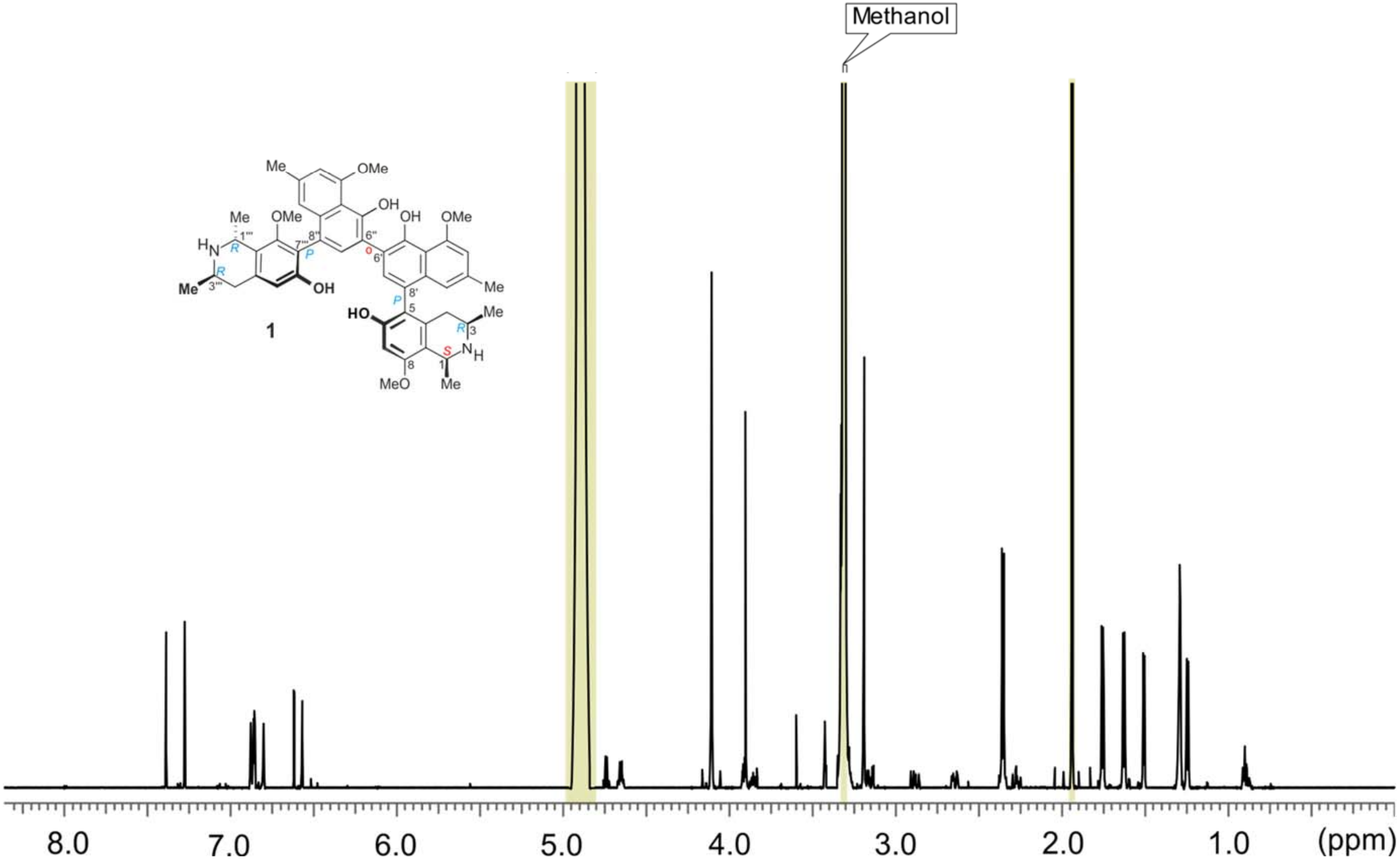

Figure S7a. Overall ^{1}H NMR spectrum of ealapasamine A (**1**) in methanol-*d*4.

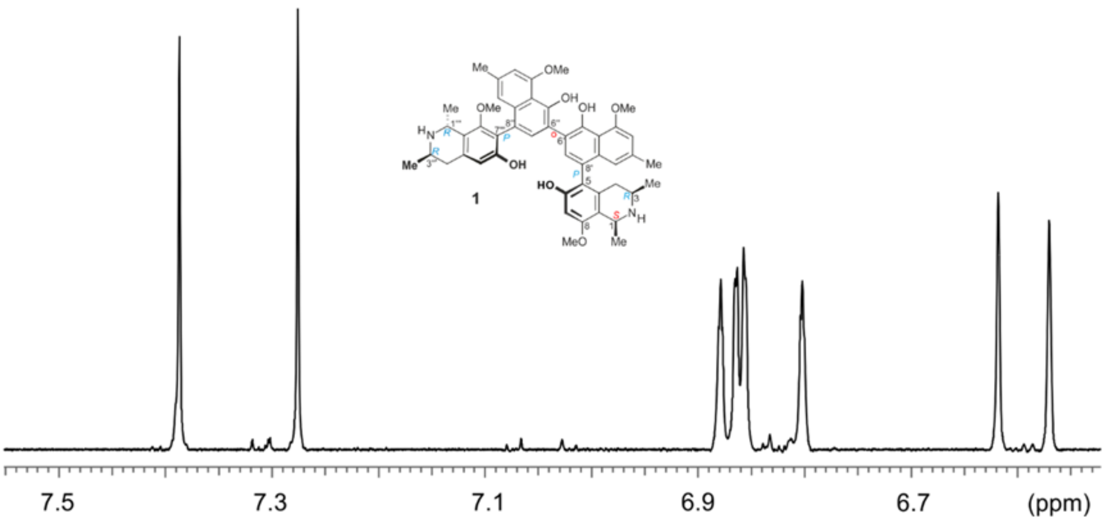

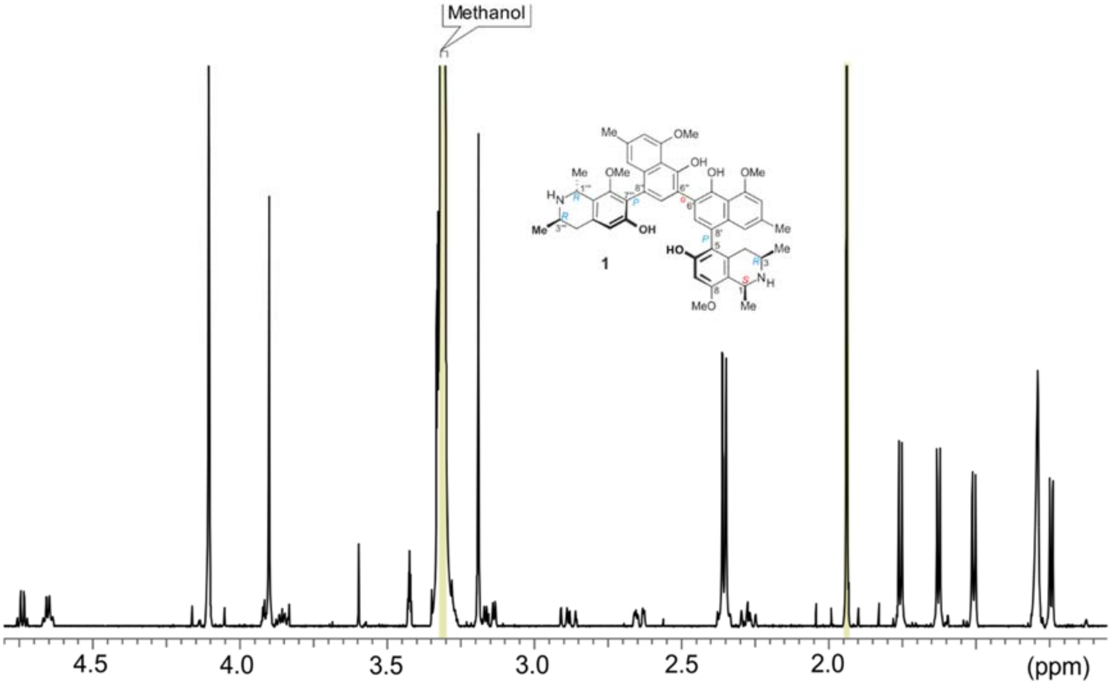

Figures S7b,c. Parts of the ^{1}H NMR spectrum of ealapasamine A (**1**) in methanol-d_4.

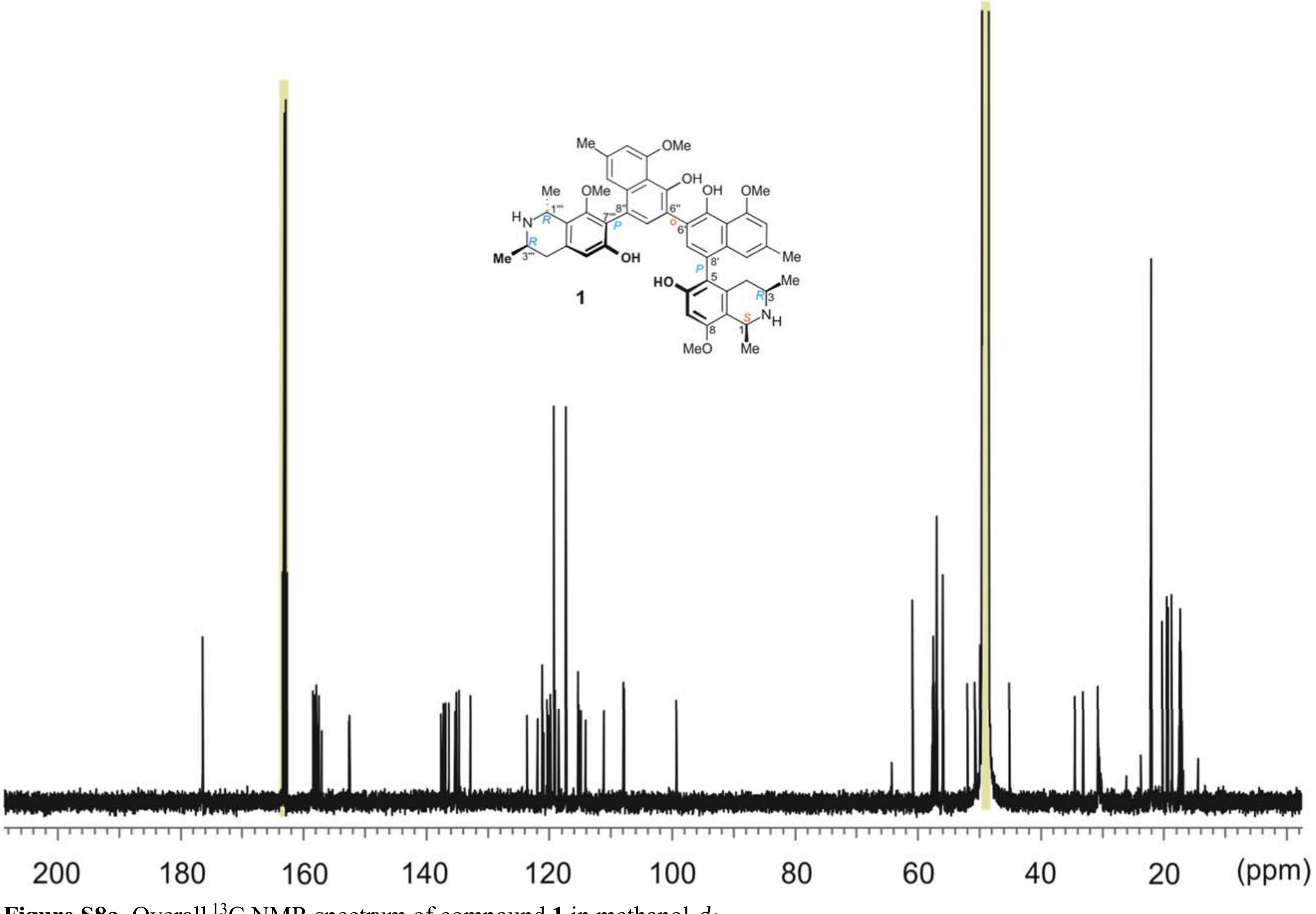

Figure S8a. Overall ^{13}C NMR spectrum of compound **1** in methanol-d_4.

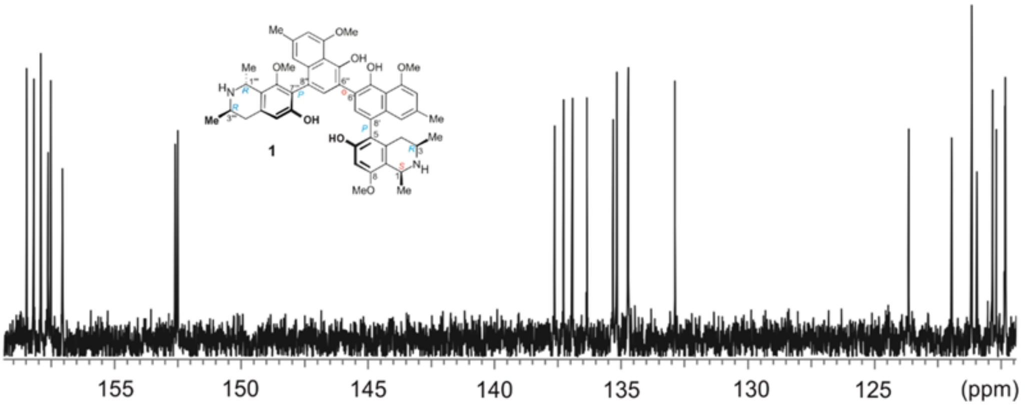

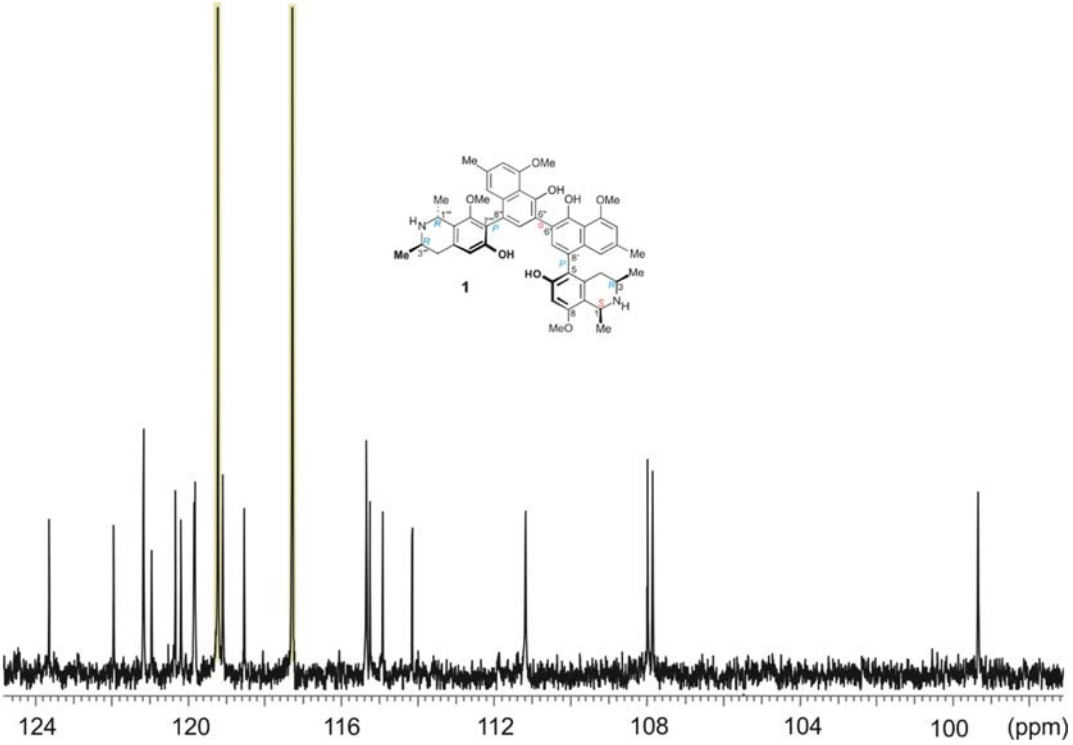

Figures S8b,c. Parts of the ^{13}C NMR spectrum of compound **1** in methanol-d_4.

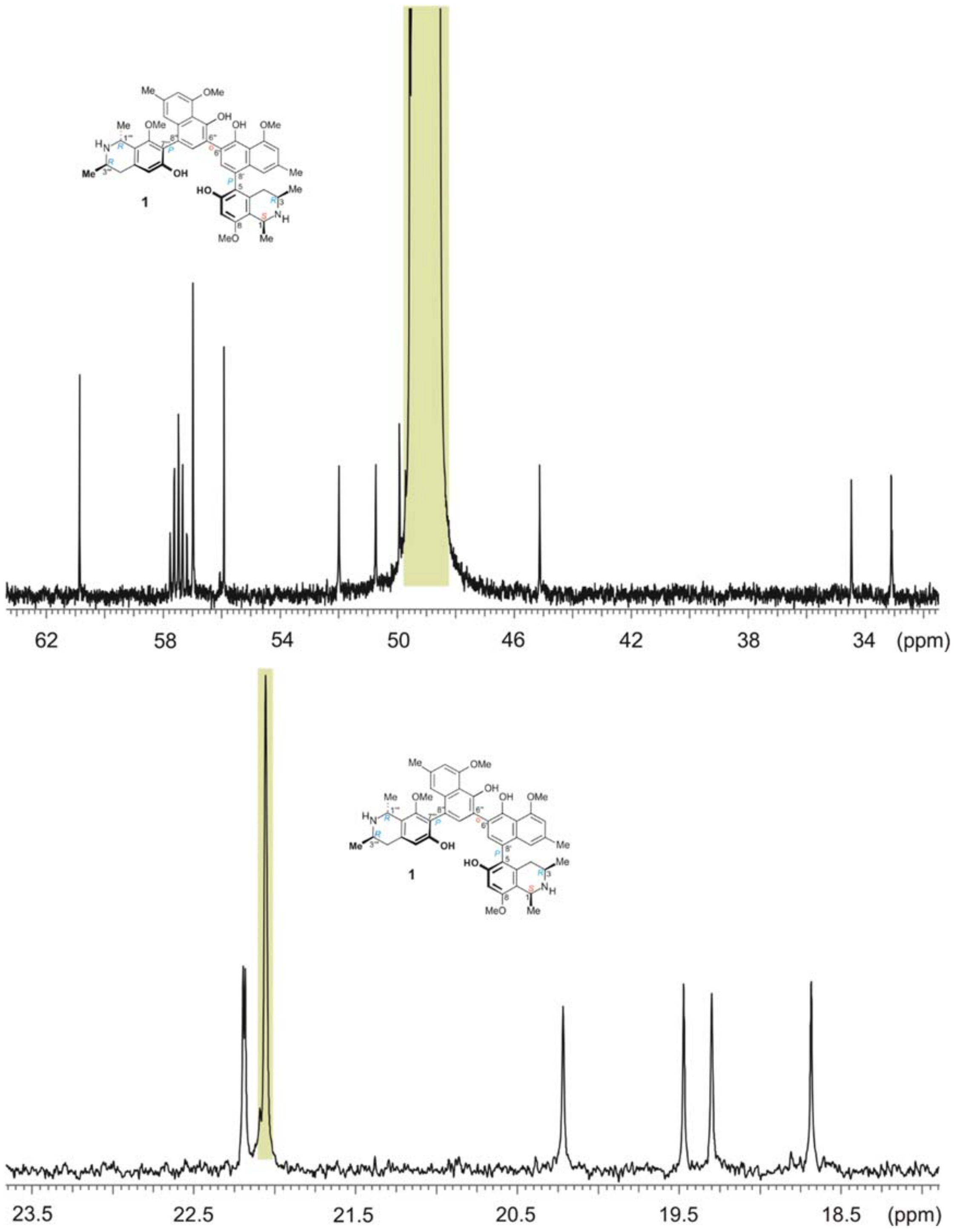

Figures S8d,e. Parts of the ^{13}C NMR spectrum of compound **1** in methanol-*d*4.

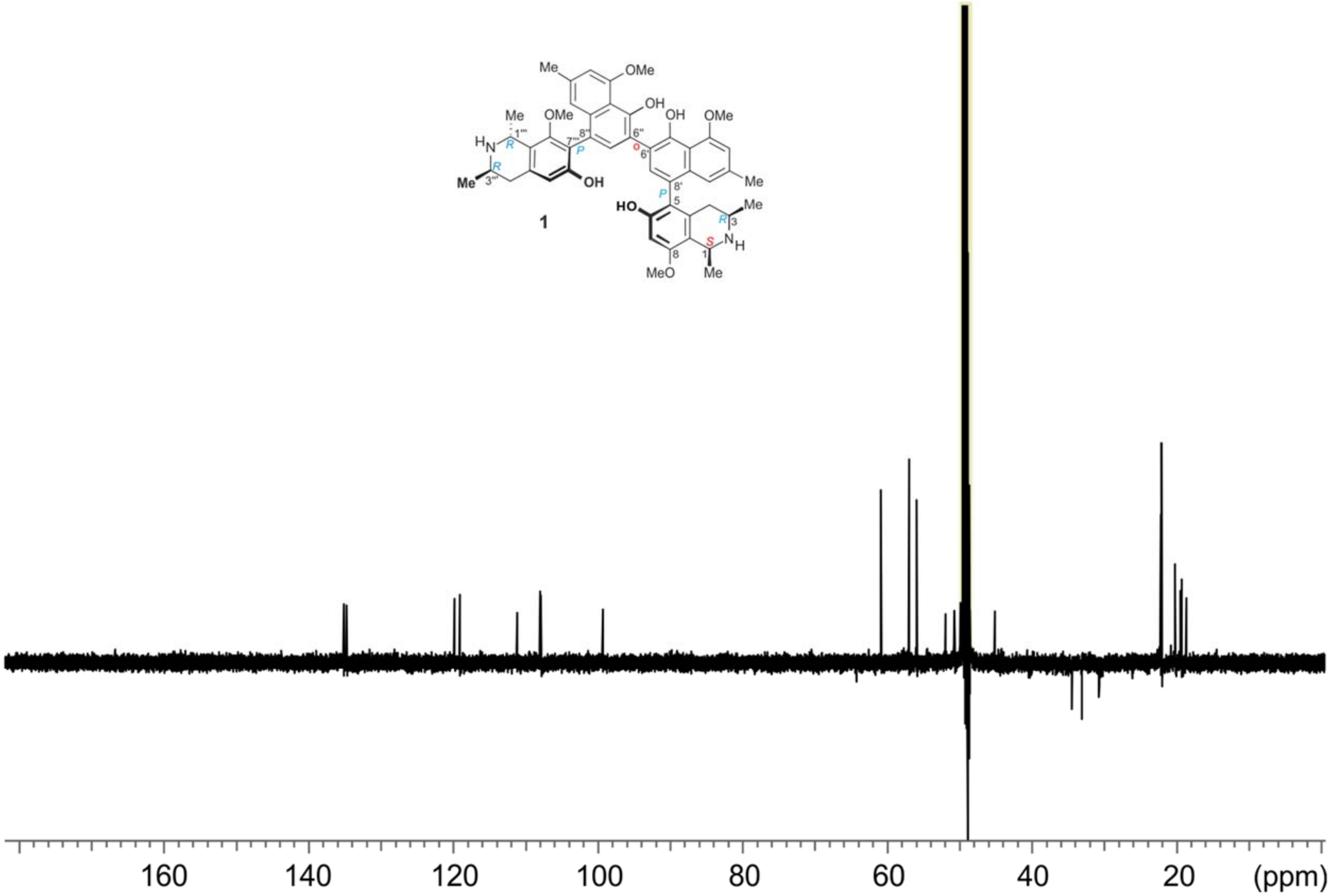

Figure S9. DEPT-135 NMR spectrum of compound **1** in methanol-d_4.

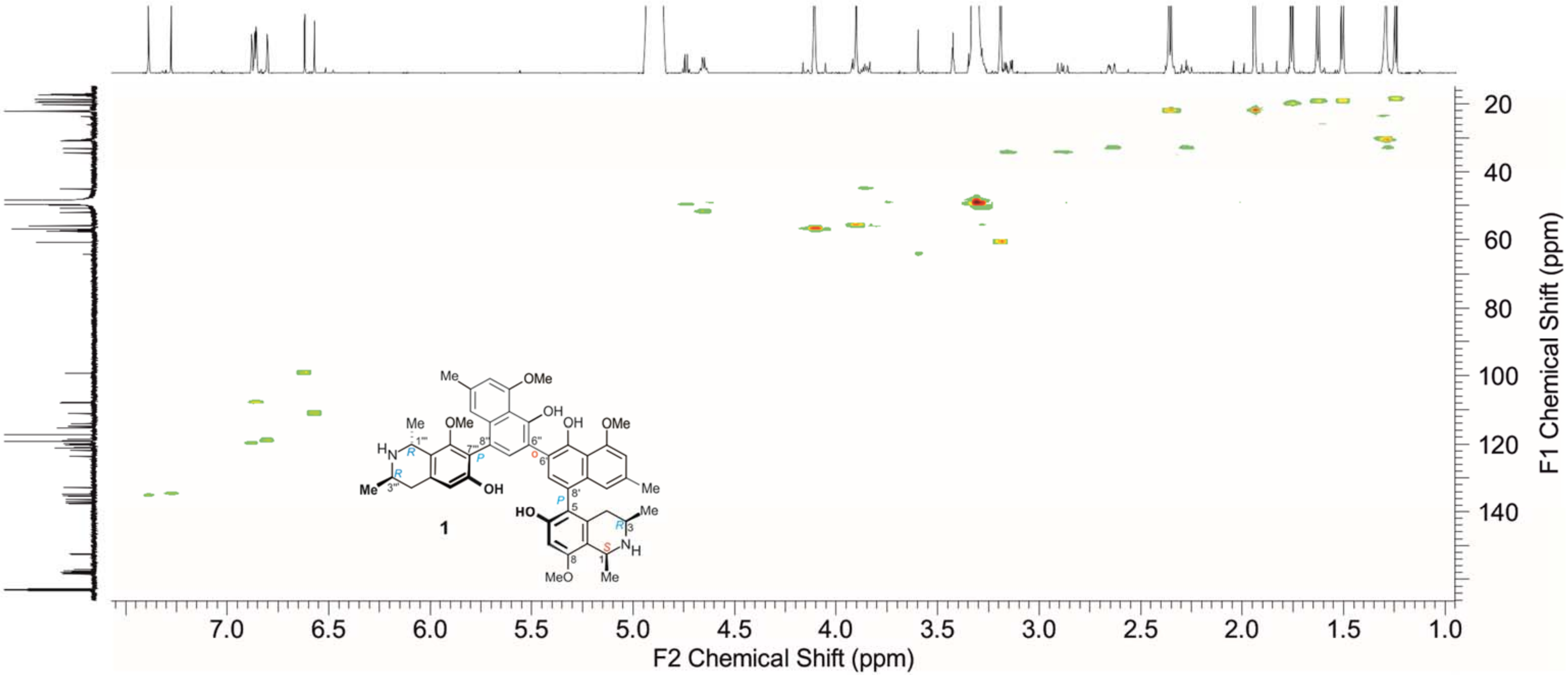

Figure S10. HSQC spectrum of compound **1** in methanol-d_4.

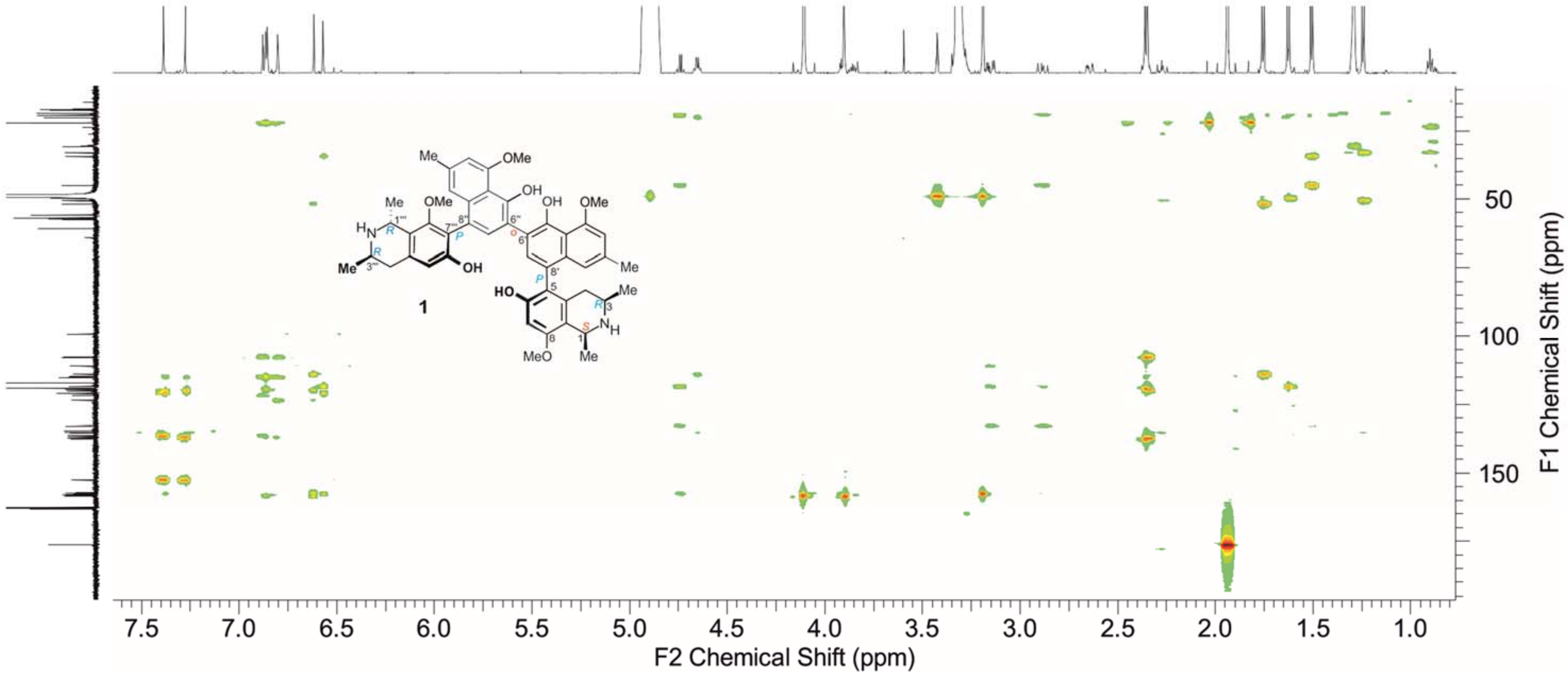

Figure S11. HMBC spectra of compound **1** in methanol-*d*4.

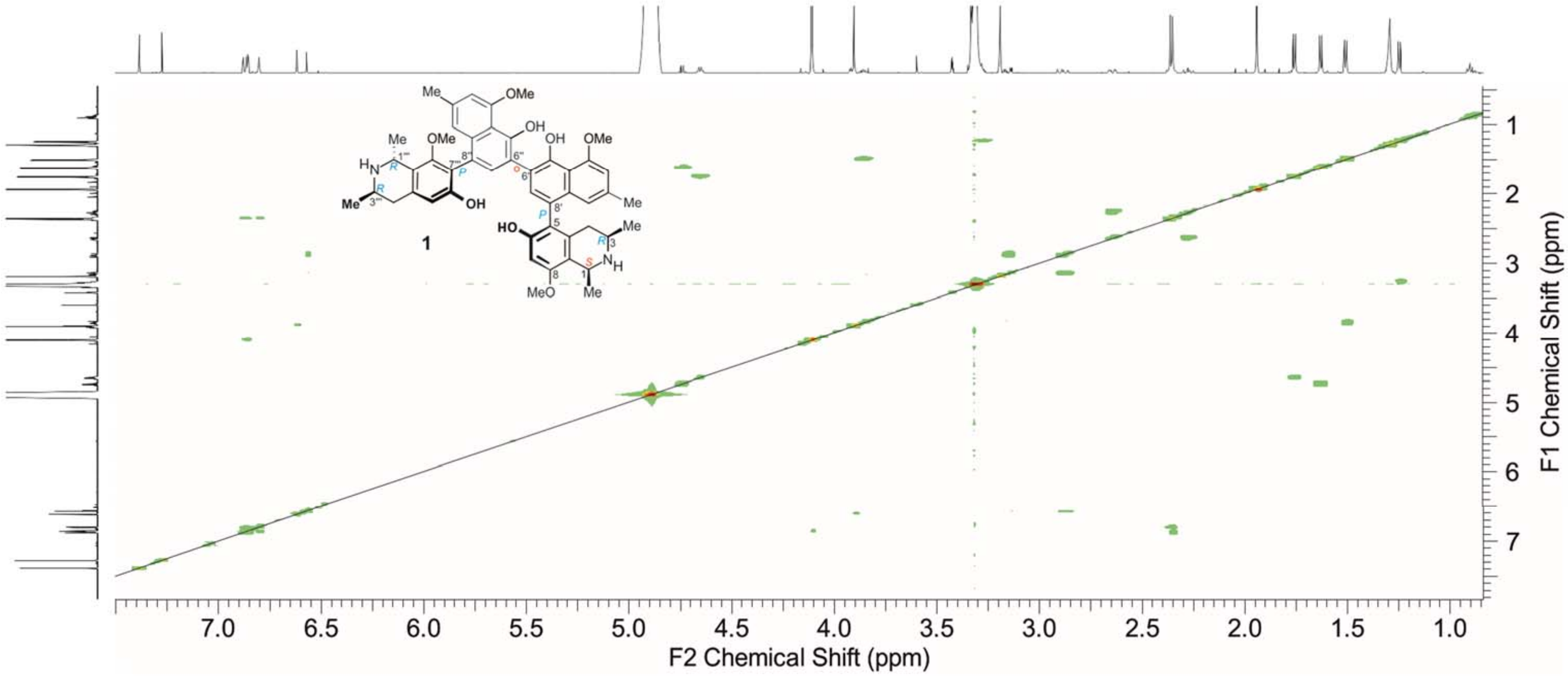

Figure S12. COSY spectra of compound **1** in methanol-d_4.

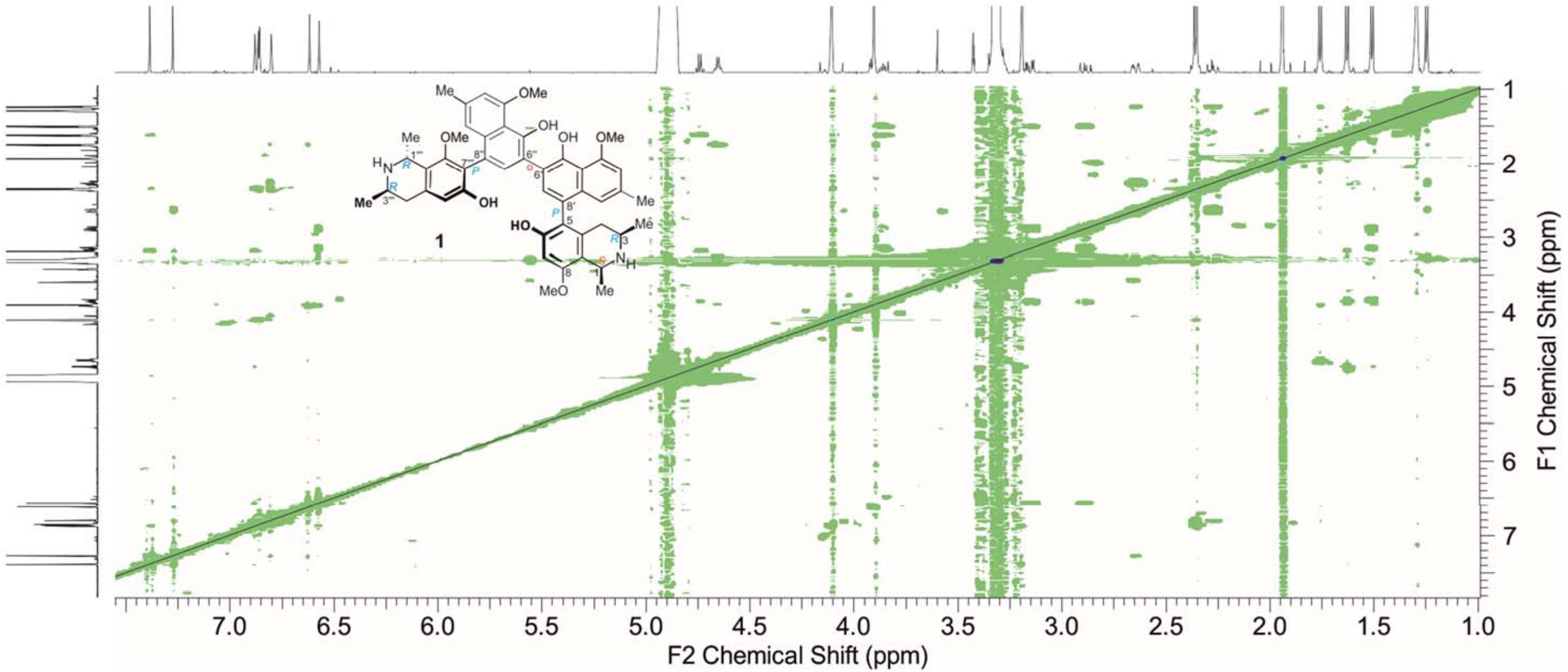

Figure S13a. Overall ROESY spectrum of compound **1** in methanol-d_4.

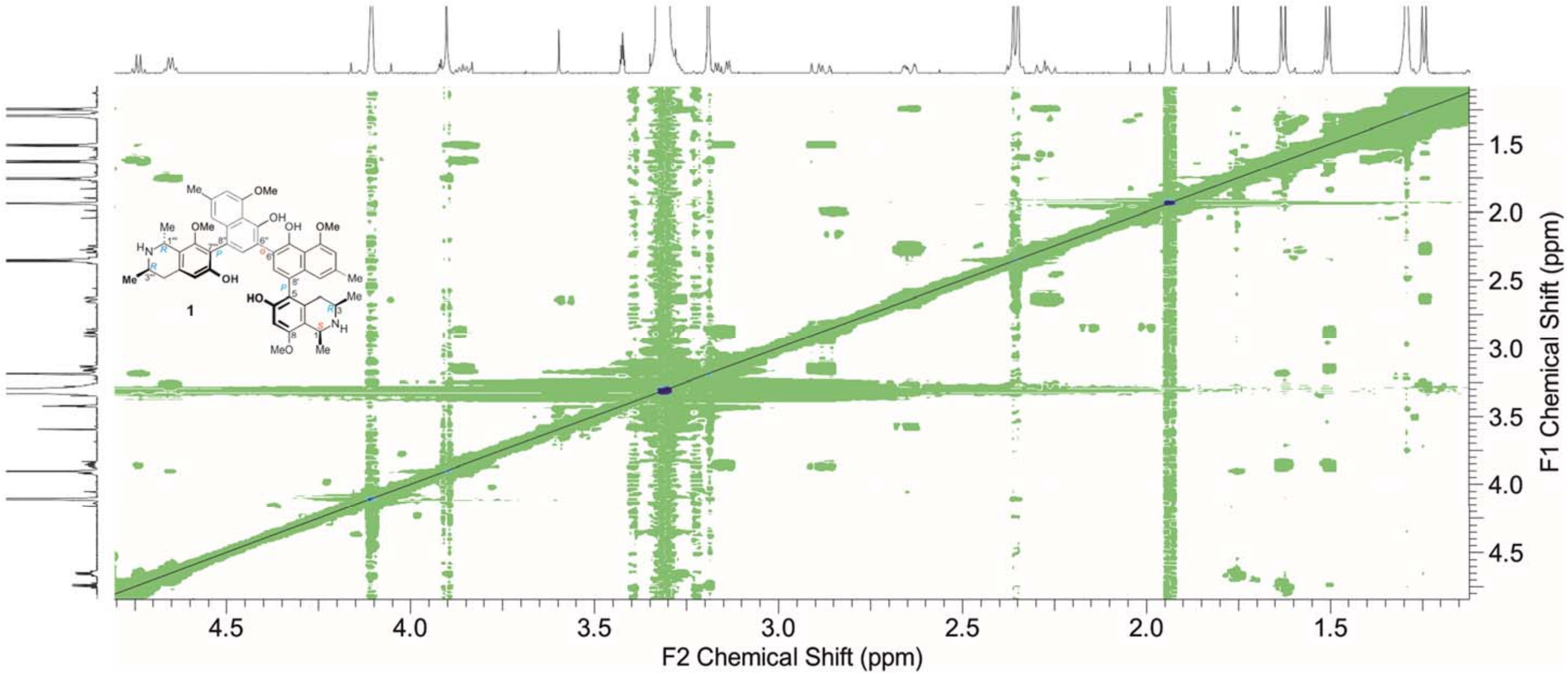

Figure S13b. Part of the ROESY spectrum of compound **1** in methanol-d_4.

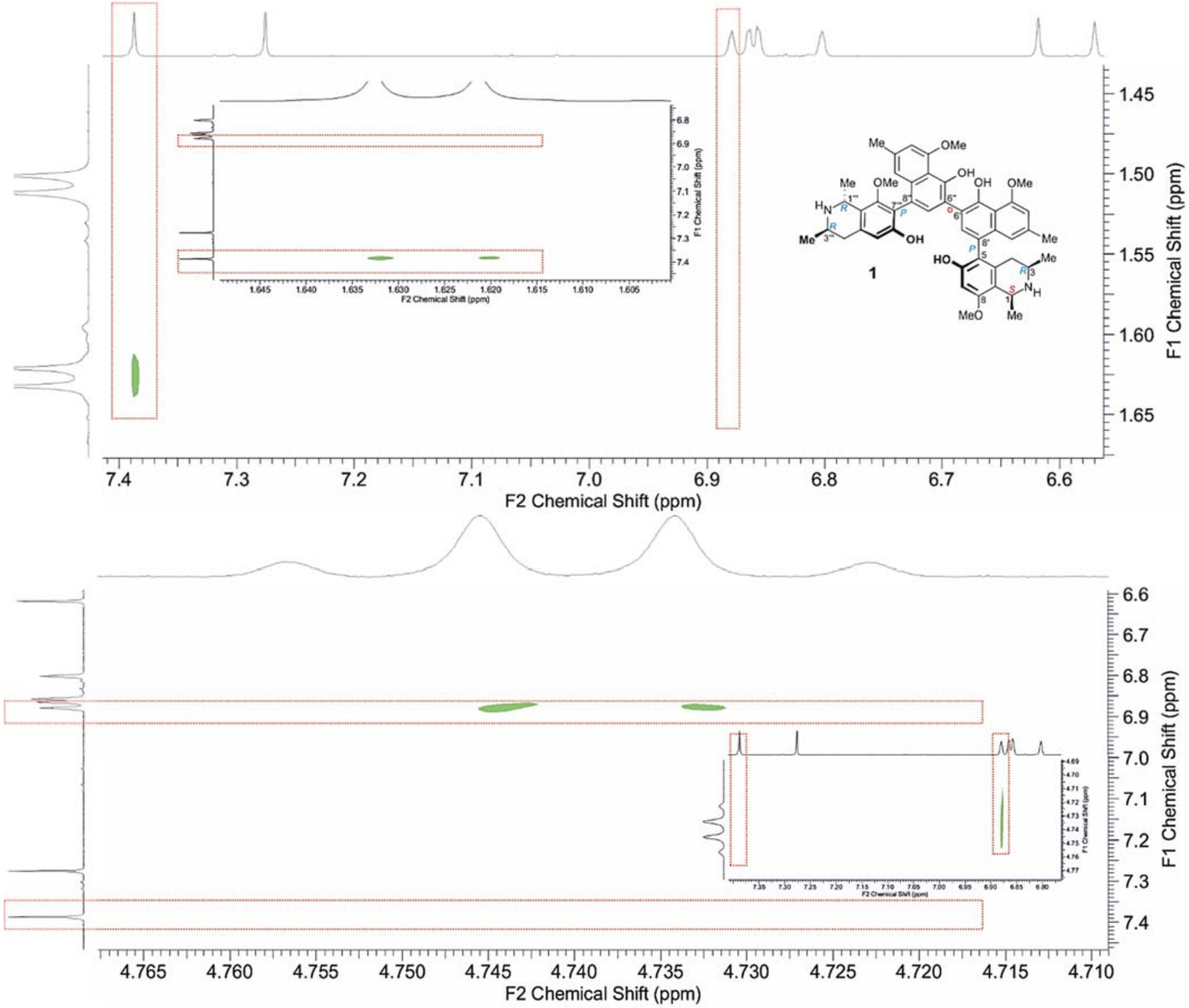

Figure S13c. Key interactions in red boxes on the ROESY spectrum for the axial configuration of compound **1**.

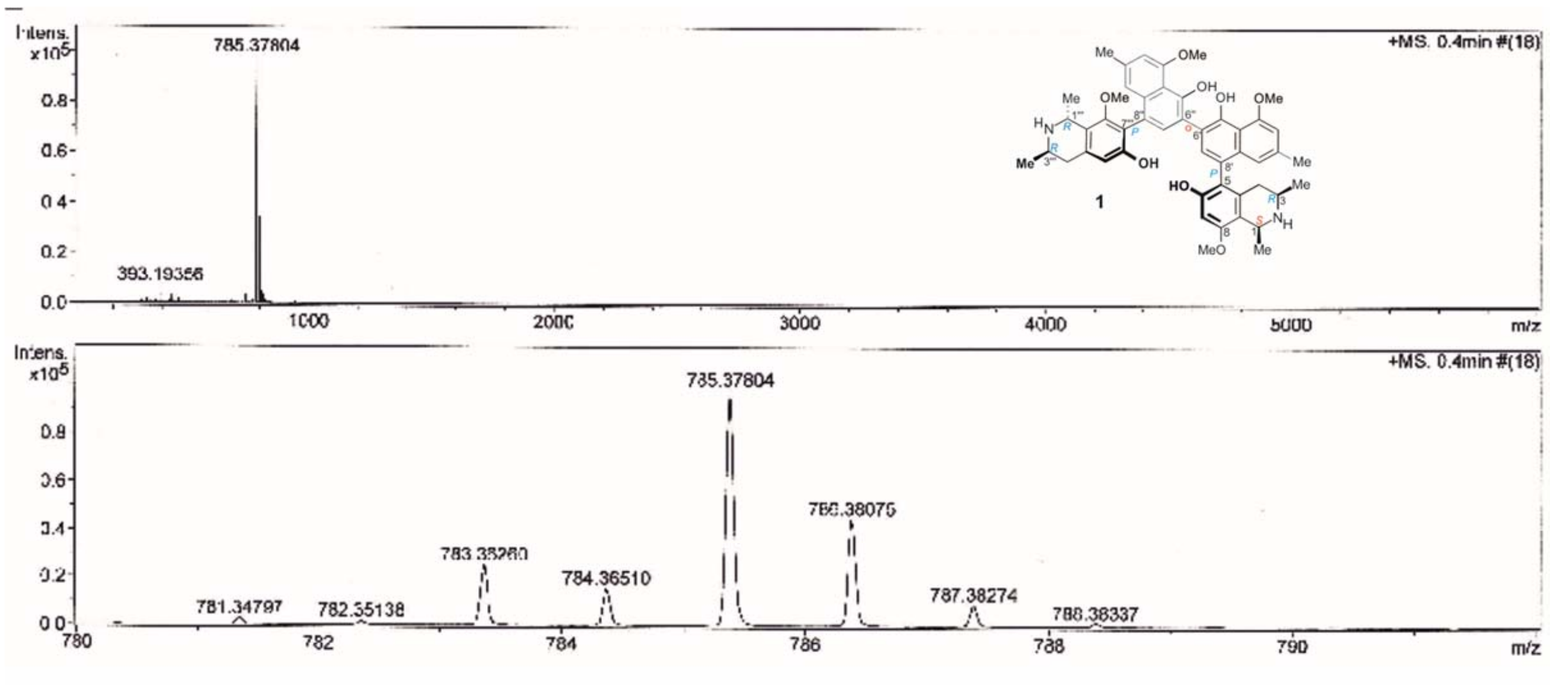

Comment	Dieudonne Tshitenge<br>AELV-13-7-P0-P8<br>undiluted	Instrument	micrOTOF

Sum Formula	Sigma	m/z	Err [ppm]	Mean Err [ppm]	rdb	N Rule	e⁻
C 48 H 53 N 2 O 8	0.05	785.37964	2.04	3.06	23.50	ok	even

Figure S14. HRESIMS spectrum of compound **1** in methanol.

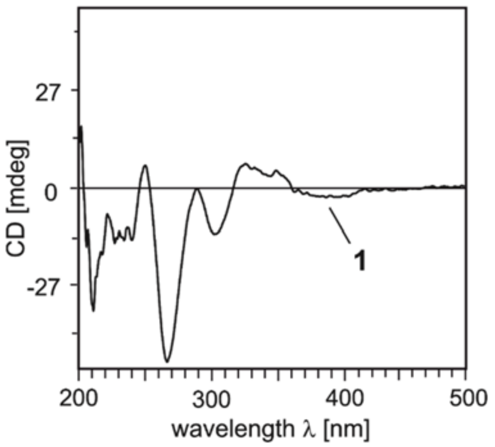

Figure S15. ECD spectrum of compound **1** in methanol.

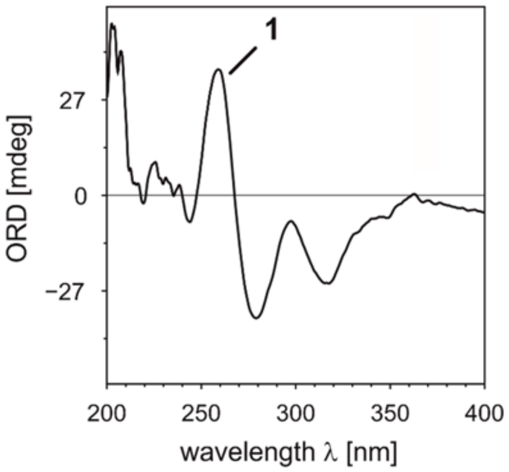

Figure S16. ORD-E spectrum of compound **1** in methanol.

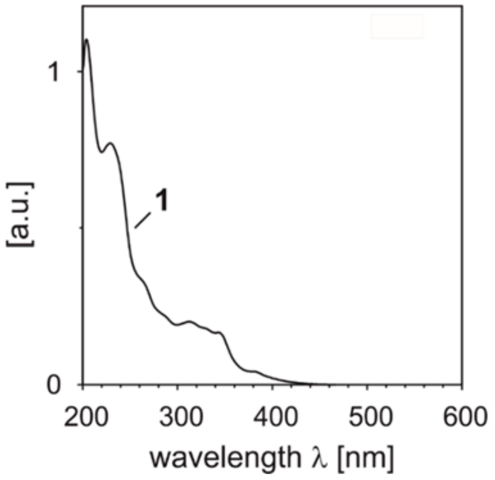

Figure S17. Offline UV spectrum of compound **1** in methanol.

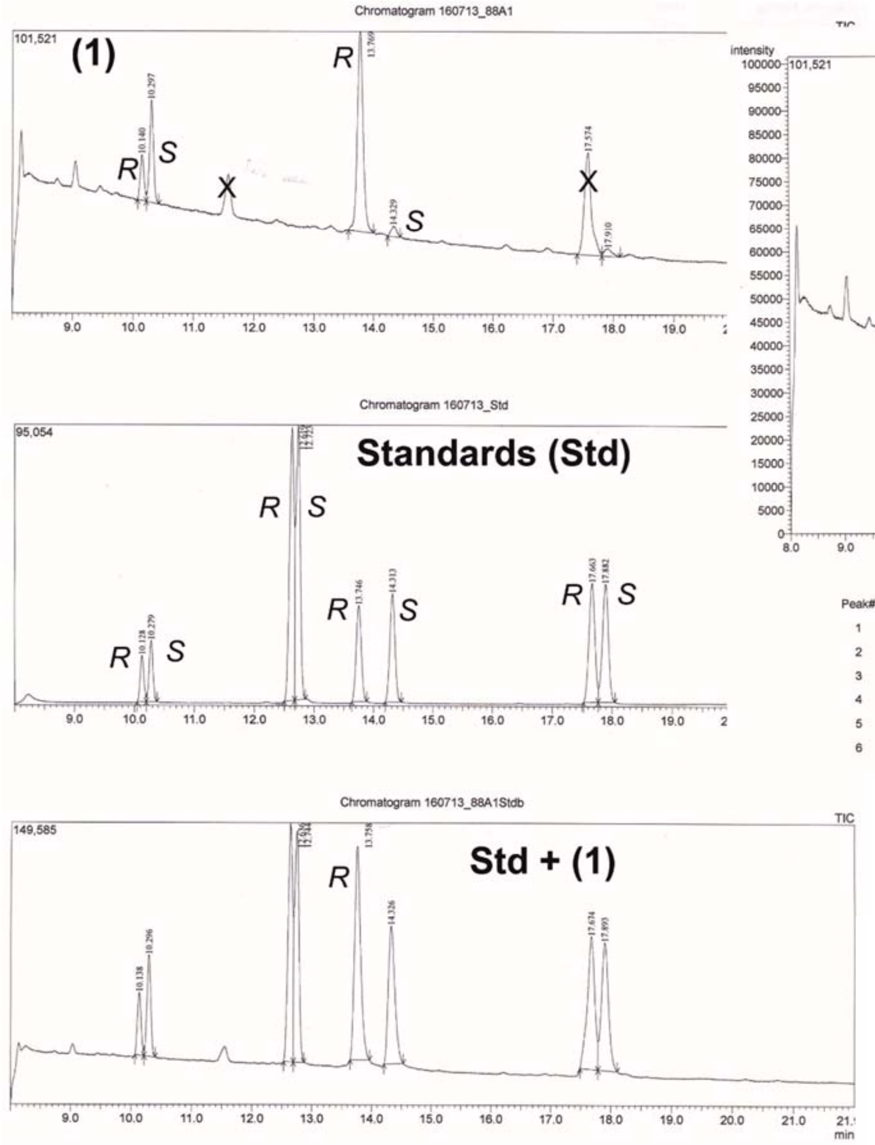

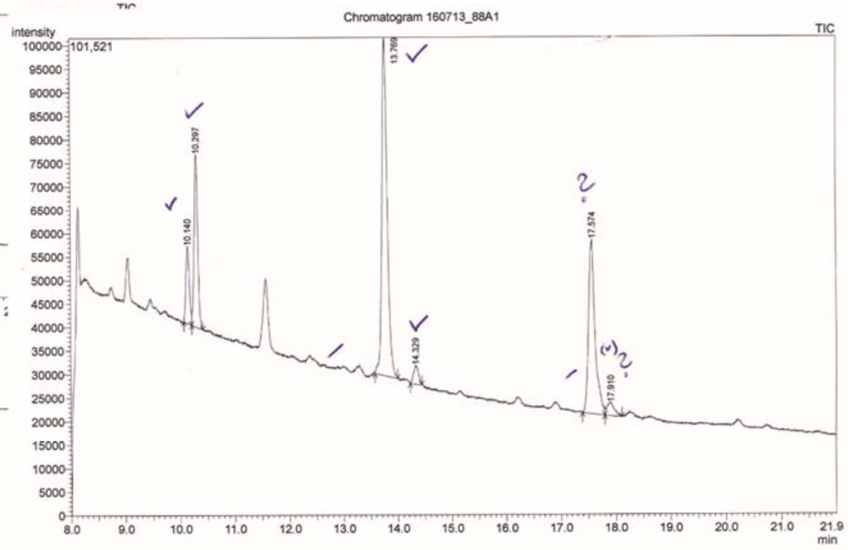

Peak#	Name	R.Time	I.Time	F.Time	Area	Area%	Height	Height%
1		10.140	10.070	10.215	70904	6.55	16429	9.71
2		10.297	10.215	10.420	165543	15.28	36827	21.77
3		13.769	13.575	13.995	491423	45.37	71908	42.51
4		14.329	14.230	14.440	25389	2.34	4088	2.42
5		17.574	17.395	17.805	303784	28.04	37083	21.92
6		17.910	17.805	18.115	26206	2.42	2826	1.67
					1083249	100.00	169161	100.00

N-H-ABA R:S 19:1

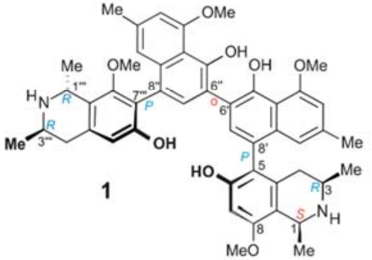

1

Figure S18. Oxidative degradation results of compound **1.**

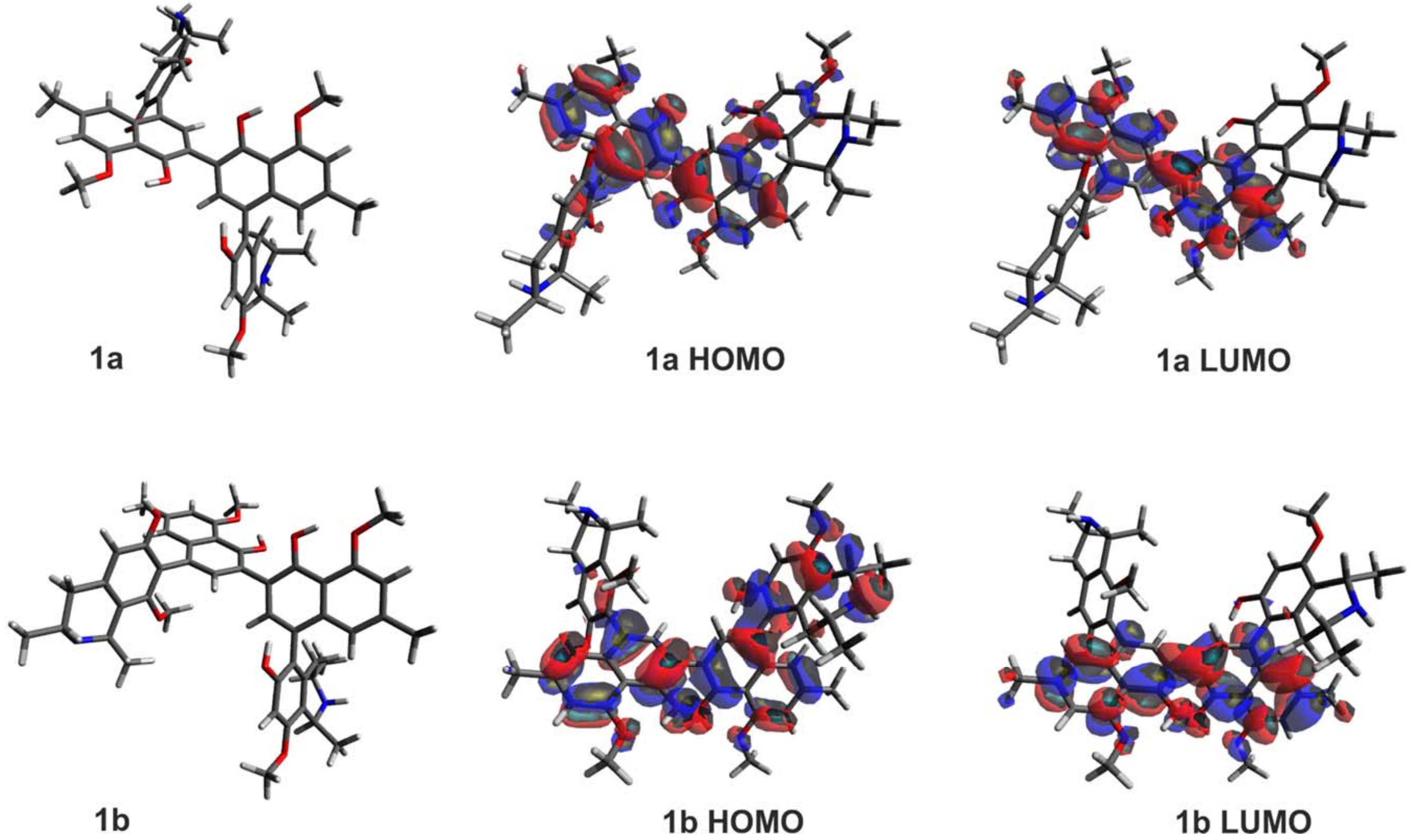

Figure S19. DFT-structural optimization of two conformers of compound **1** (**1a** and **1b**), and their HOMO and LUMO molecular orbitals. The most favorable conformer **1a** was found to have the highest HOMO-LUMO energy gap and the lowest total single point energy by DFT-calculations with B3LYP-D3/def2-TZVP.

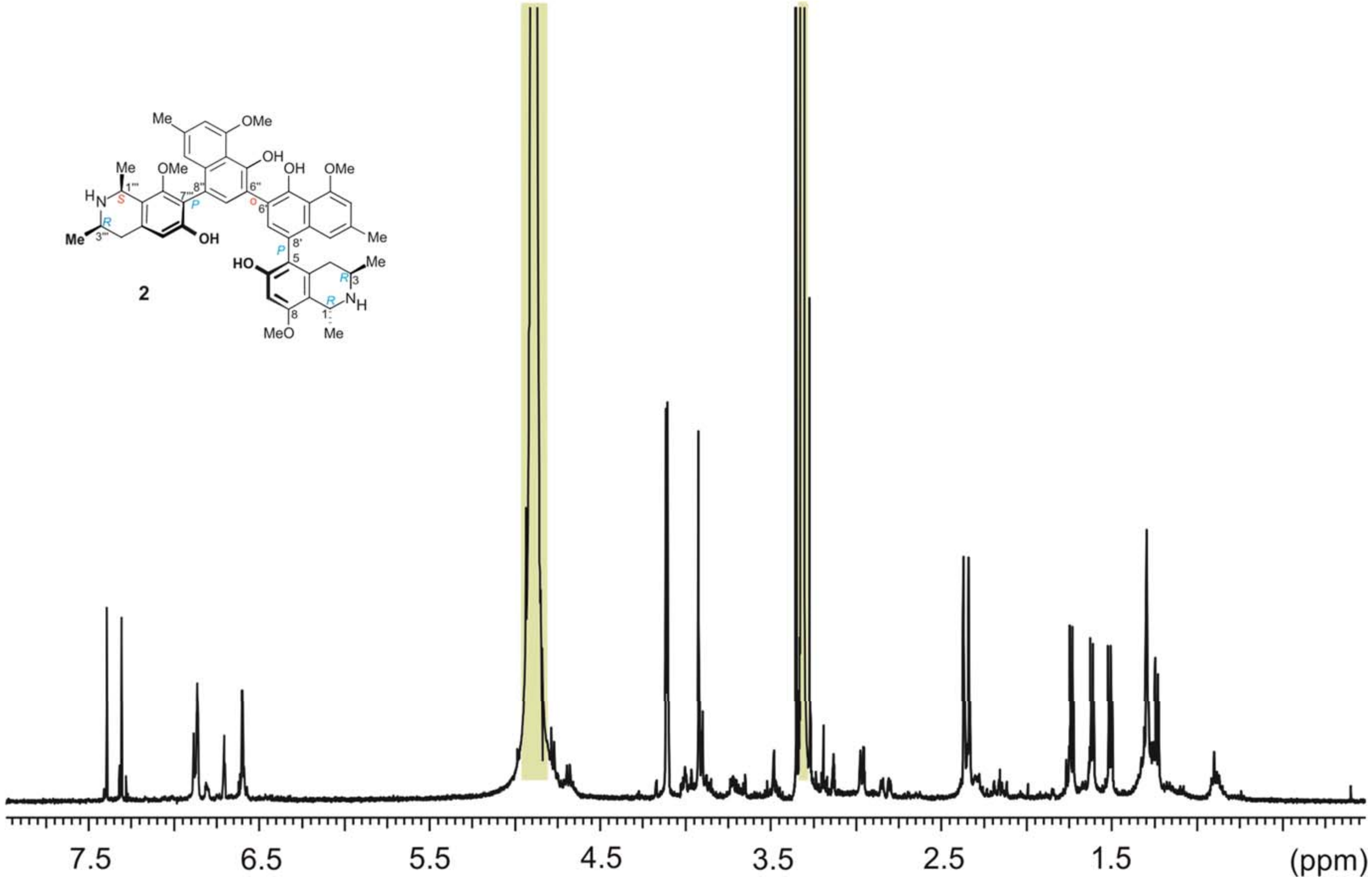

Figure S20a. Overall ^{1}H NMR spectrum of ealapasamine B **(2)** in methanol-d_4.

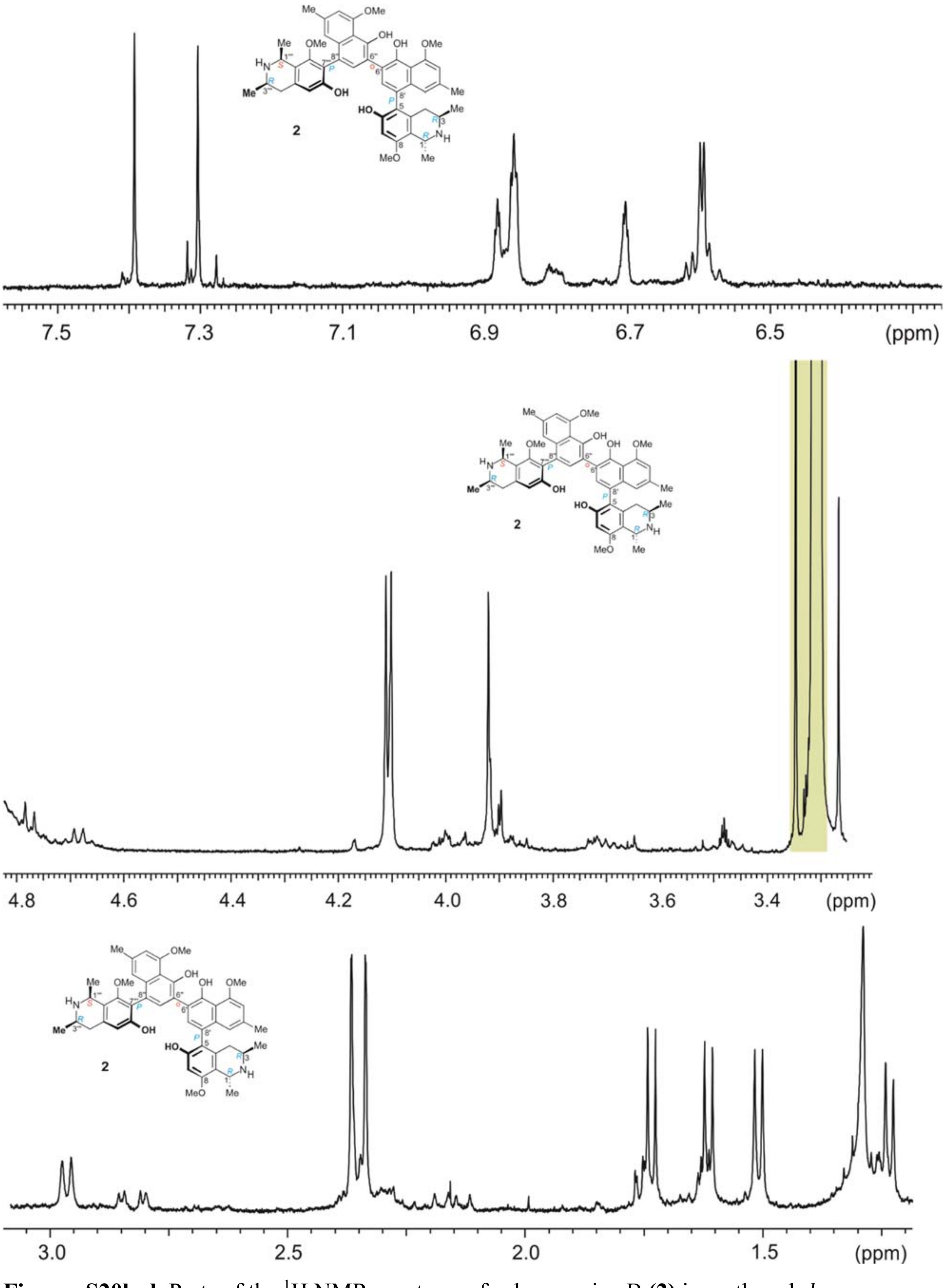

Figures S20b-d. Parts of the ^{1}H NMR spectrum of ealapasamine B **(2)** in methanol-d_4.

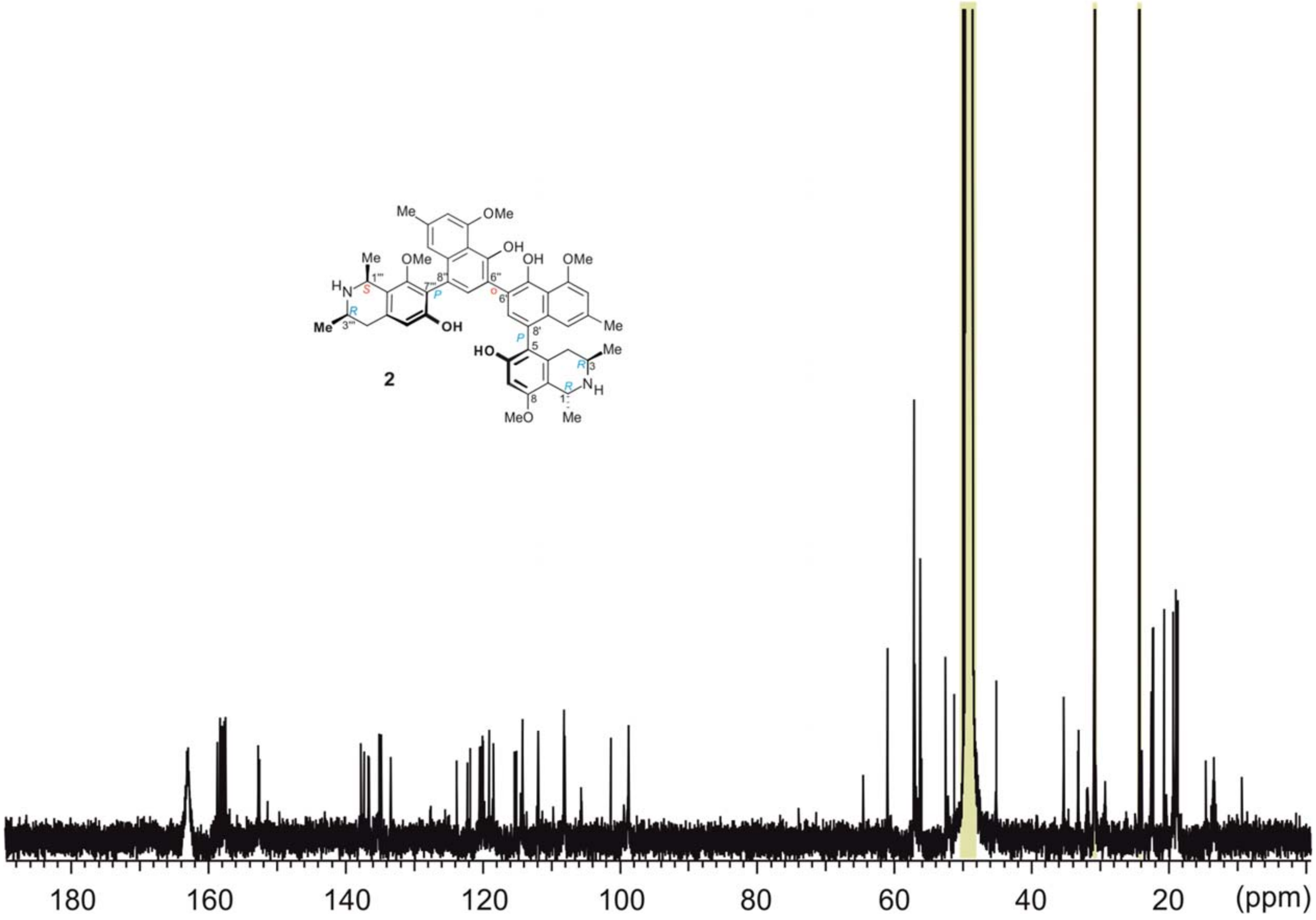

Figure S21a. Overall ^{13}C NMR spectrum of compound **2** in methanol-d_4.

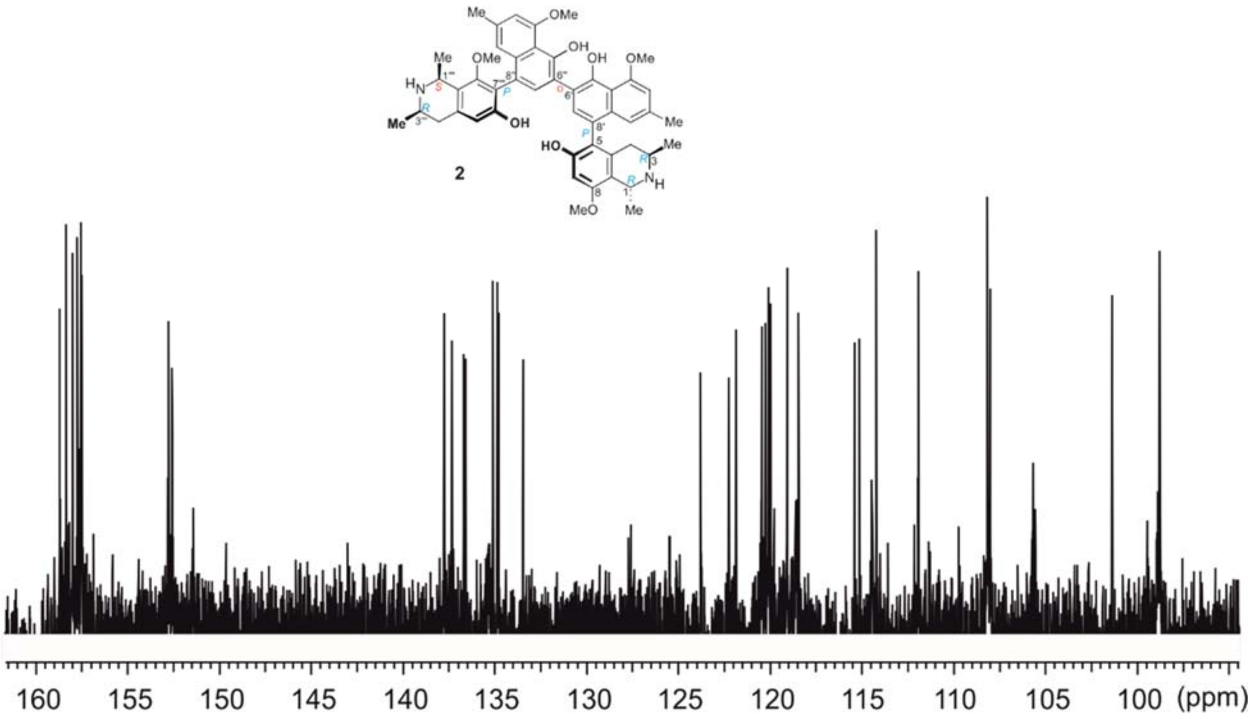

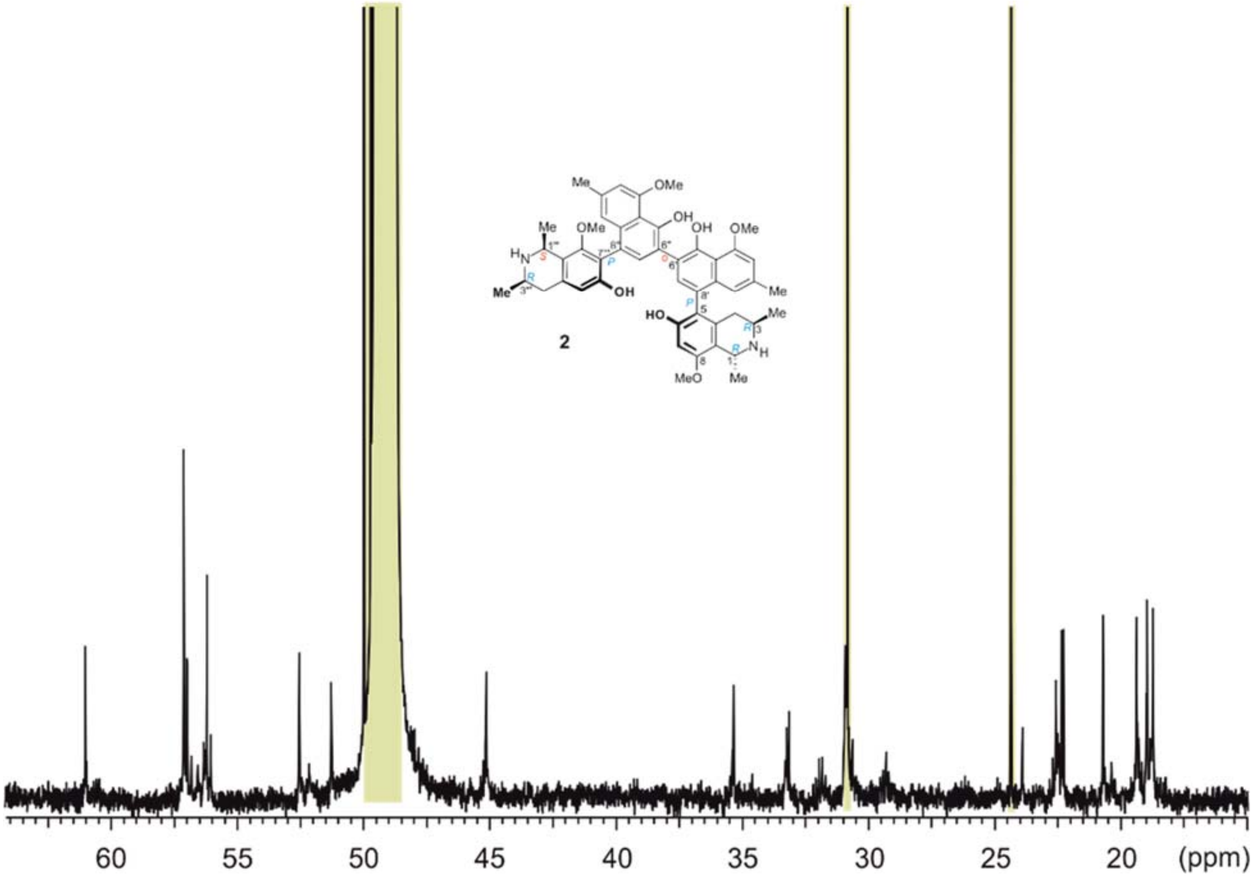

Figures S21b,c. Parts of the ^{13}C NMR spectrum of compound **2** in methanol-d_4.

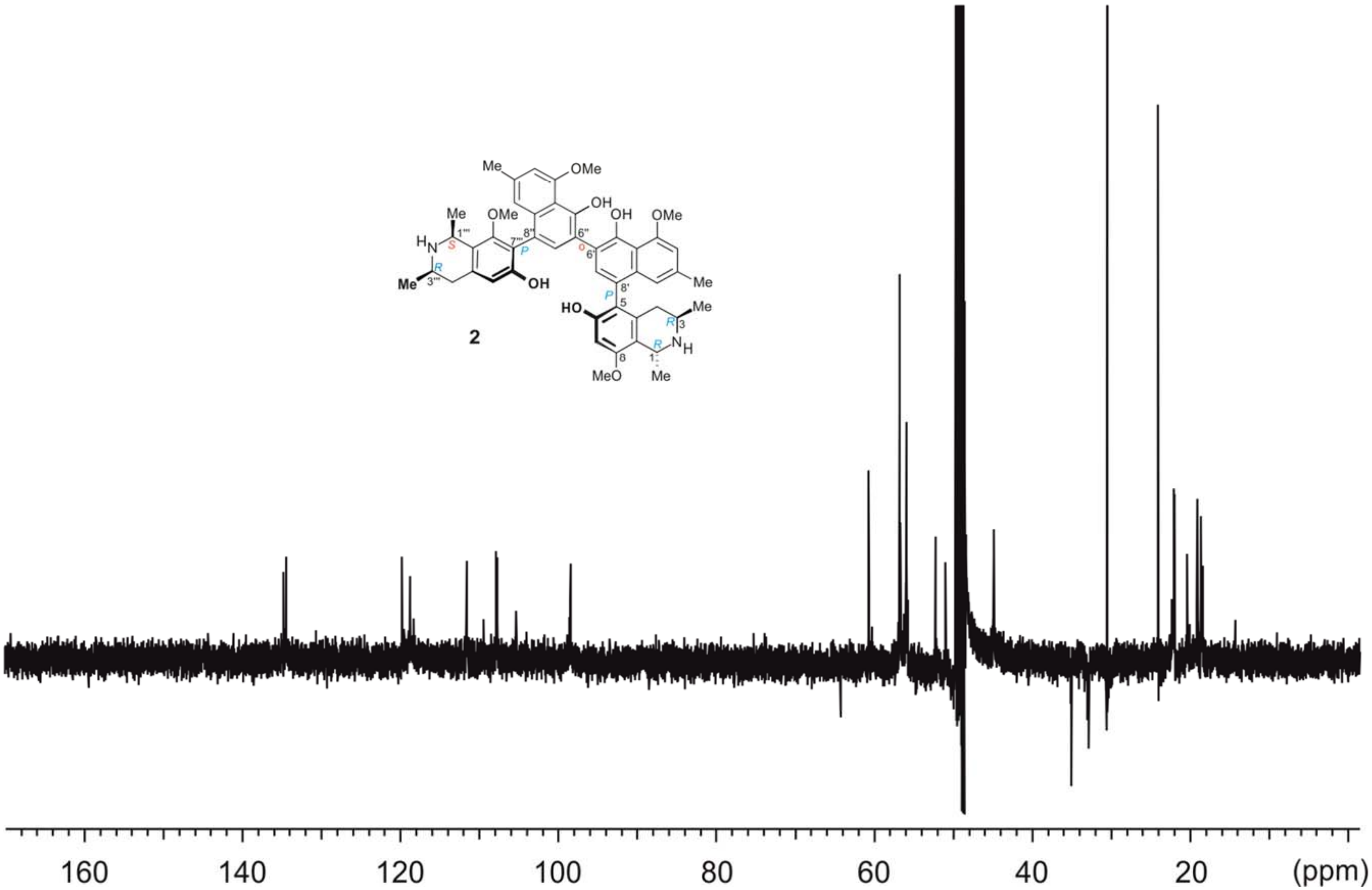

Figure S22. DEPT NMR spectrum of compound **2** in methanol-d_4.

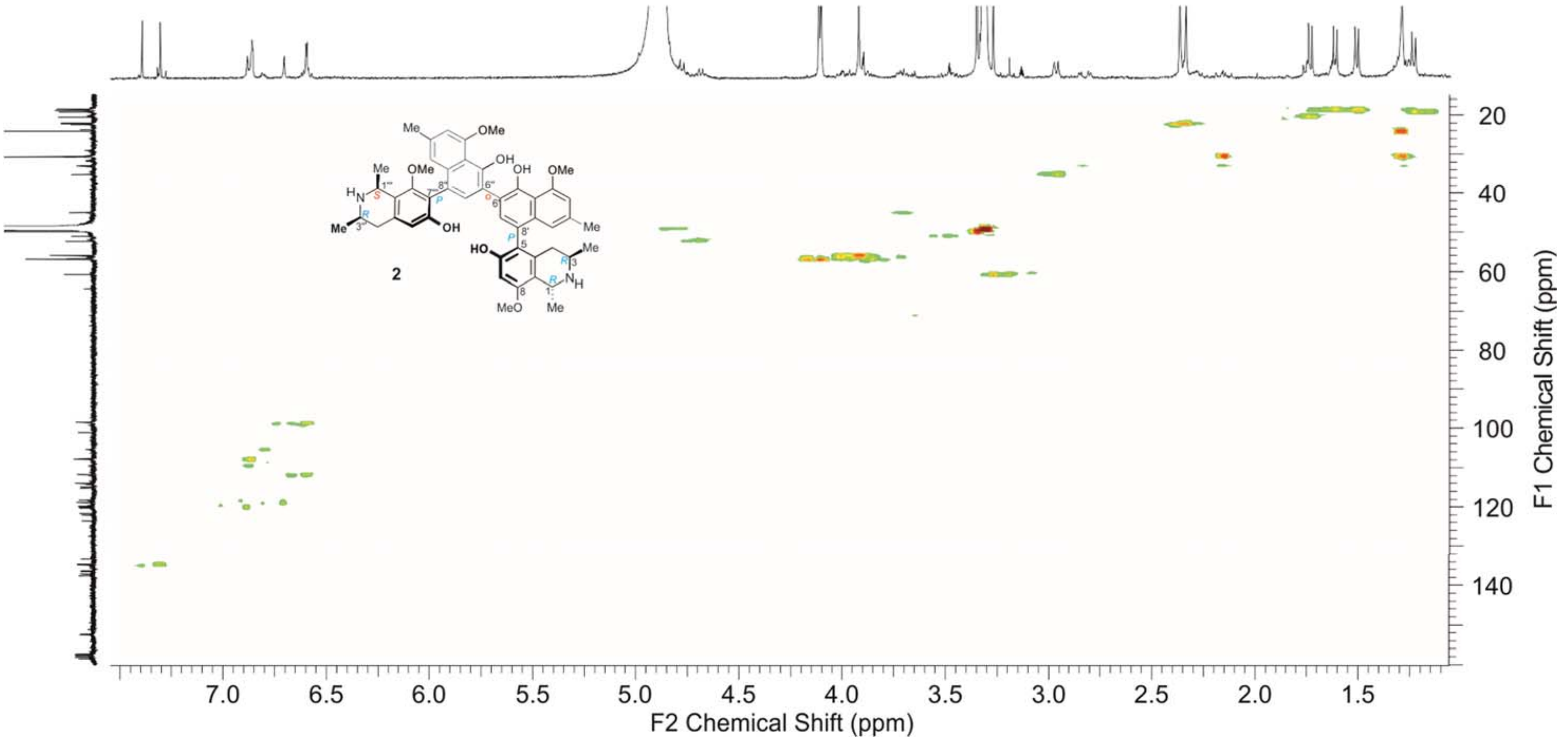

Figure S23a. Overall HSQC spectrum of compound **2** in methanol-d_4.

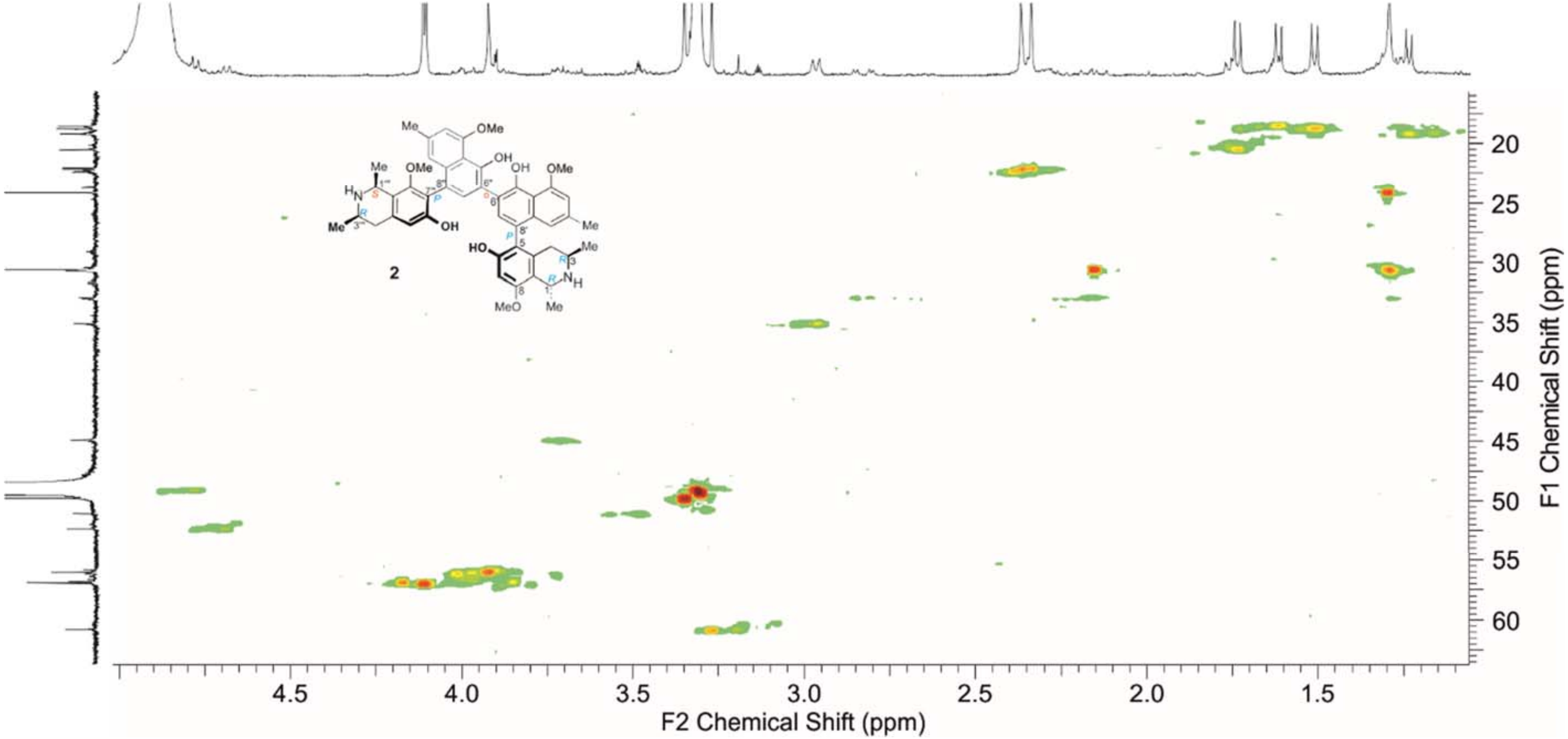

Figure S23b. Part of the HSQC spectrum of compound **2** in methanol-d_4.

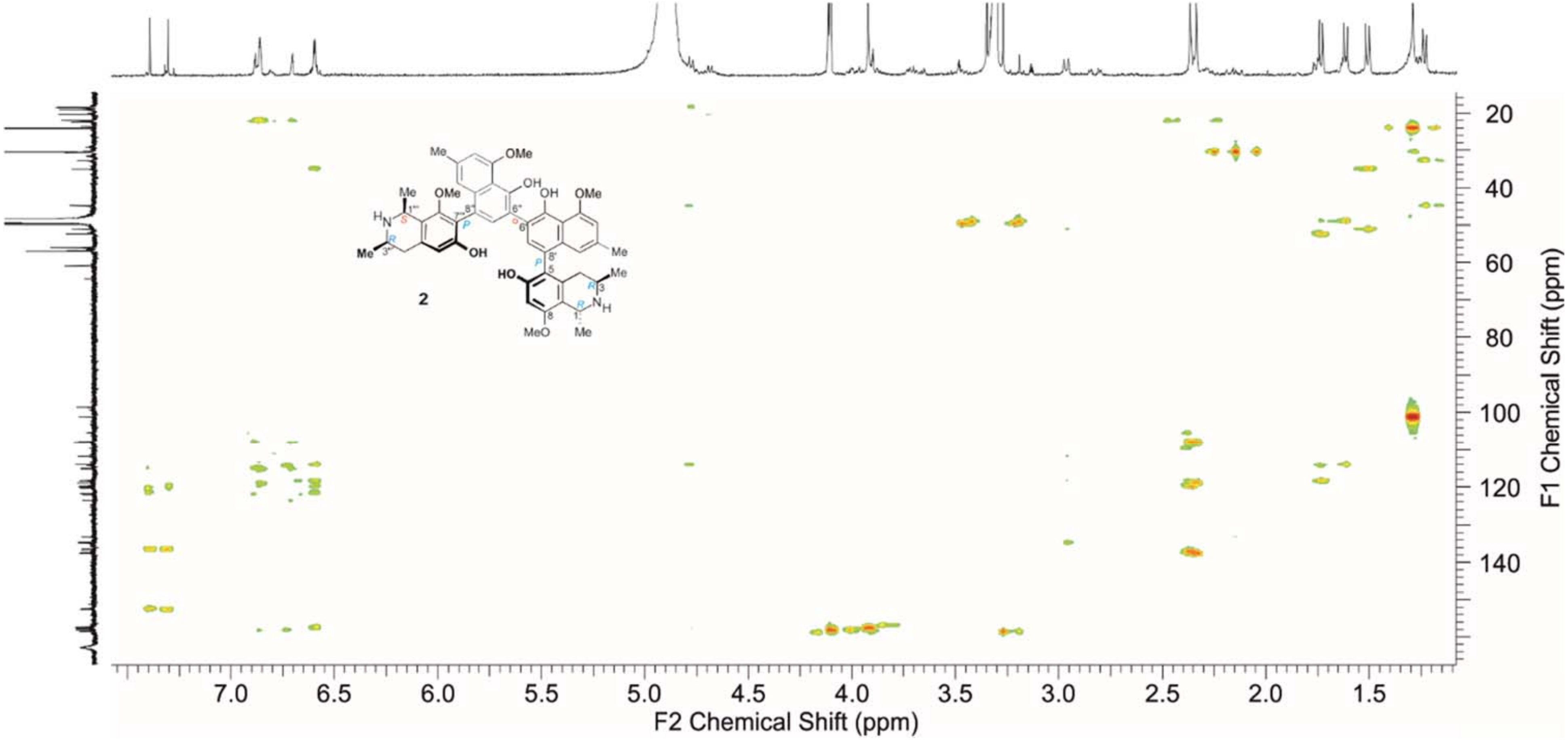

Figure S24. HMBC spectrum of compound **2** in methanol-d_4.

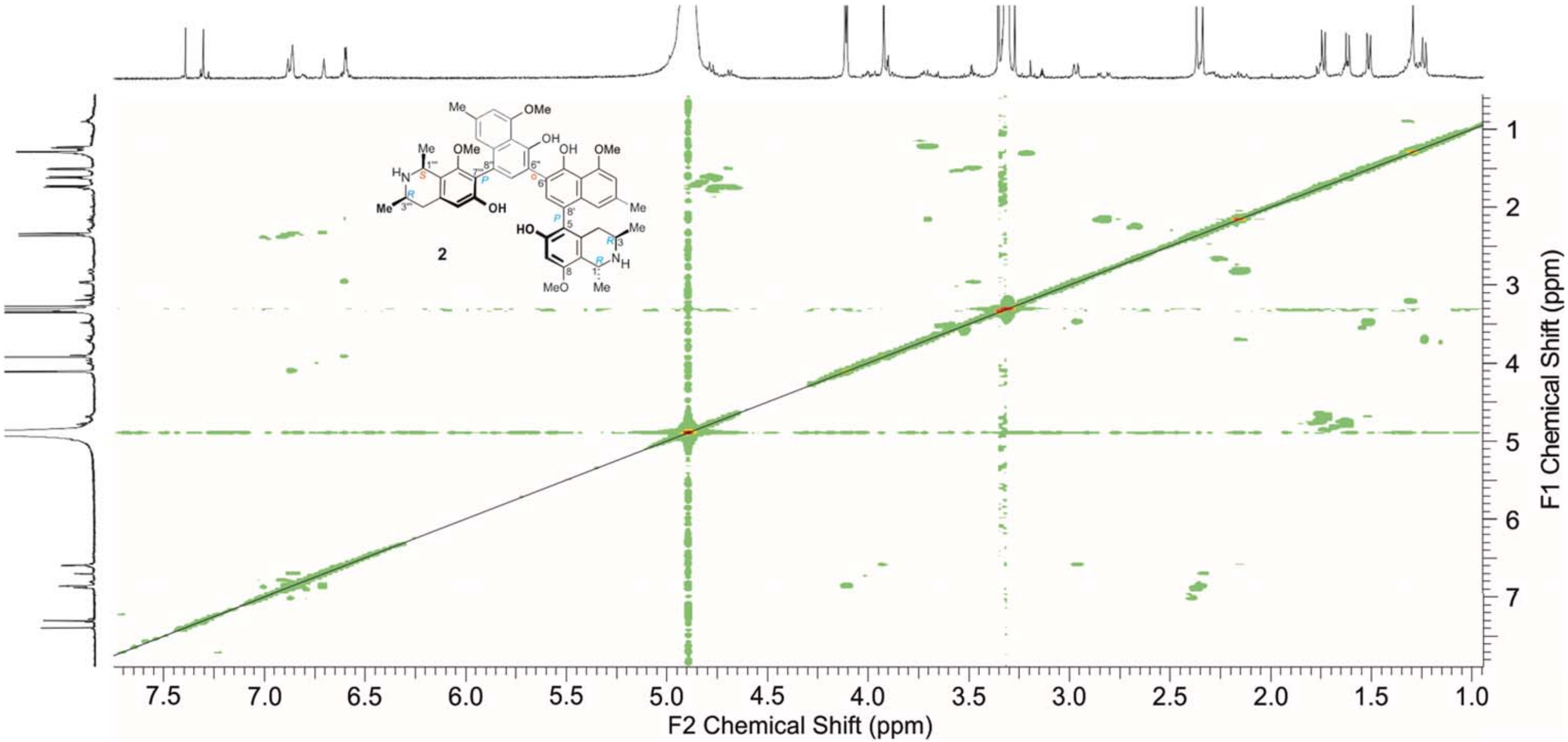

Figure S25. COSY spectrum of compound **2** in methanol-d_4.

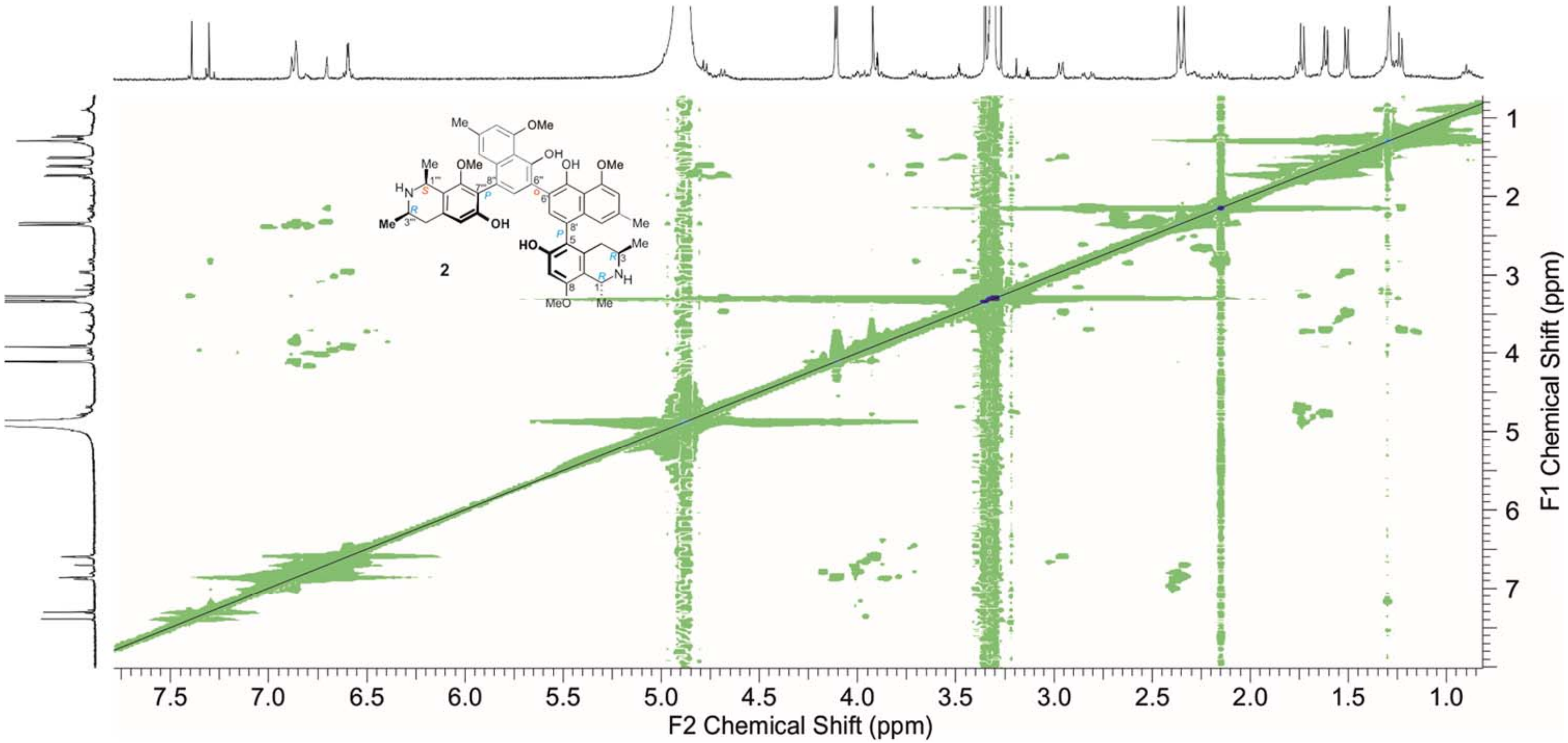

Figure S26a. Overall ROESY spectrum of compound **2** in methanol-d_4.

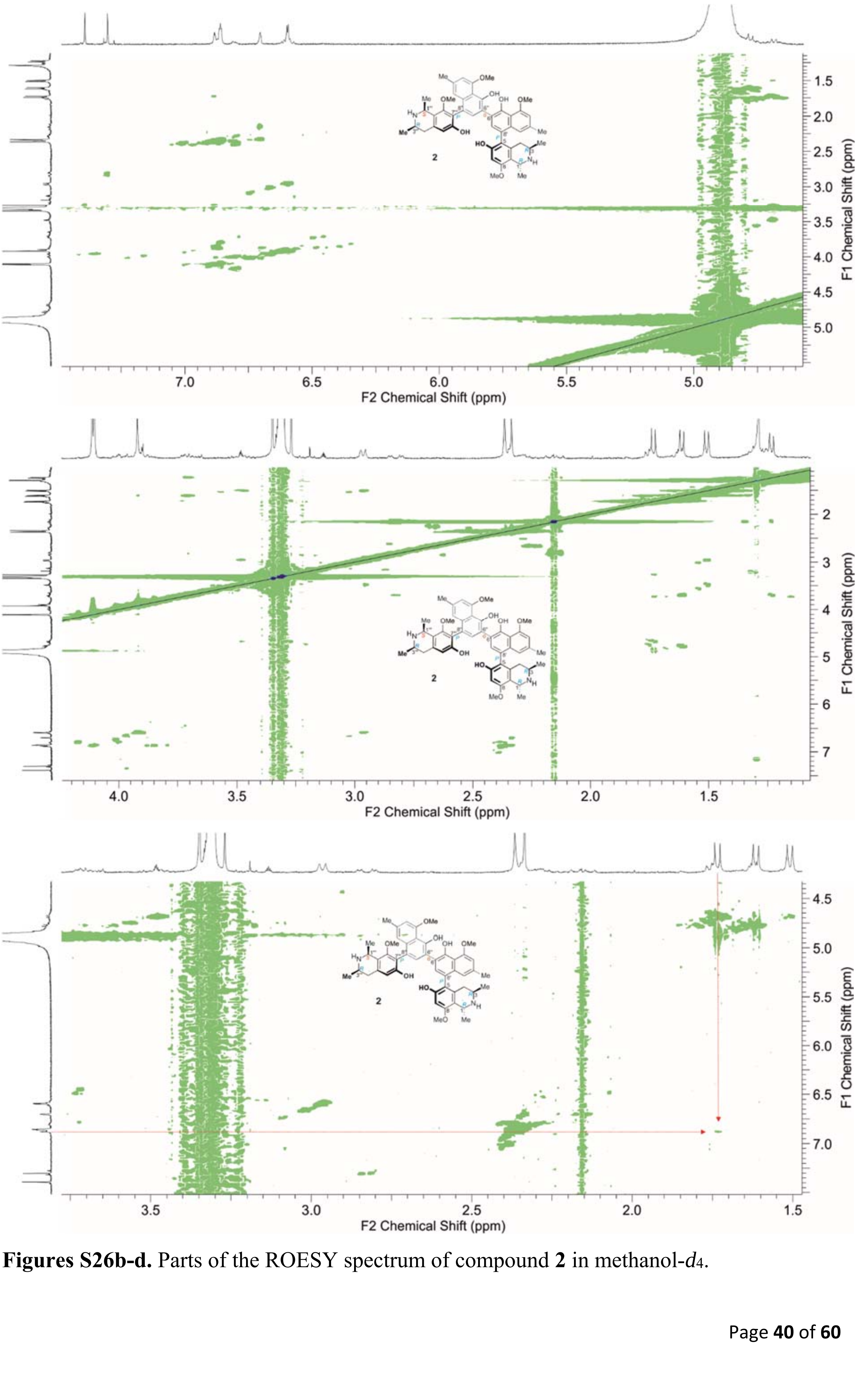

Figures S26b-d. Parts of the ROESY spectrum of compound **2** in methanol-*d*4.

Acquisition Parameter

Source Type	ESI	Ion Polarity	Positive	Set Corrector Fill	43 V
Scan Range	n/a	Capillary Exit	180.0 V	Set Pulsar Pull	804 V
Scan Begin	50 m/z	Hexapole RF	280.0 V	Set Pulsar Push	807 V
Scan End	3000 m/z	Skimmer 1	50.0 V	Set Reflector	1700 V
		Hexapole 1	23.0 V	Set Flight Tube	8600 V
				Set Detector TOF	2160 V

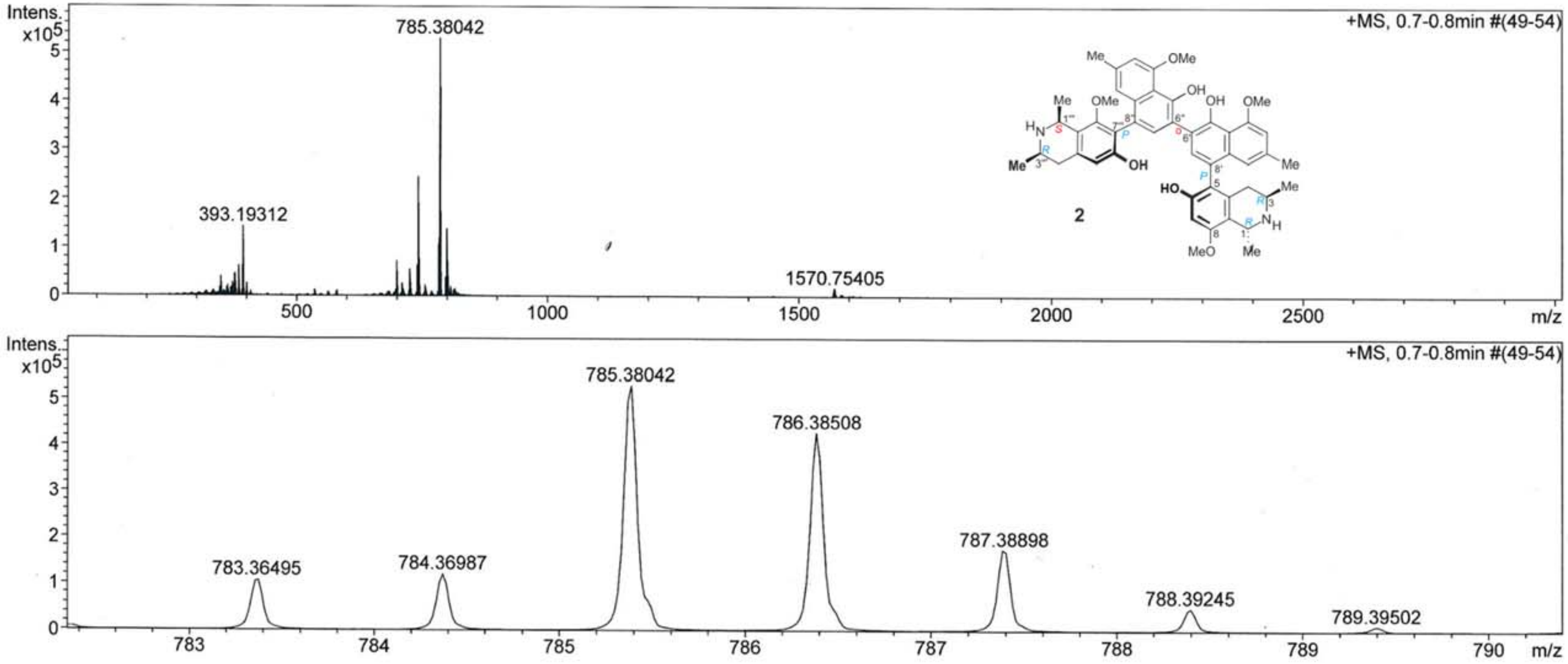

Sum Formula	Sigma	m/z	Err [ppm]	Mean Err [ppm]	rdb	N Rule	e⁻
C 48 H 53 N 2 O 8	0.16	785.37964	-0.98	-1.96	23.50	ok	even

Figure S27. HRESIMS spectrum of compound **2** in methanol.

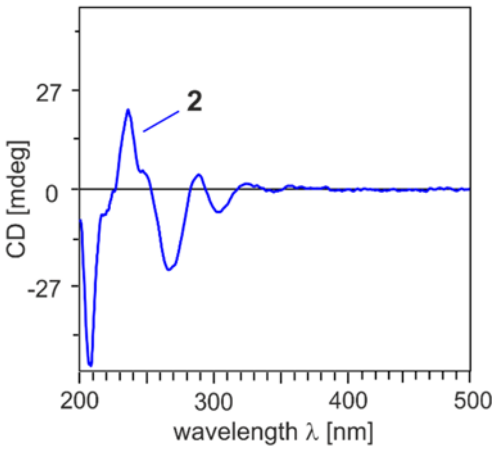

Figure S28. ECD spectrum of compound **2** in methanol.

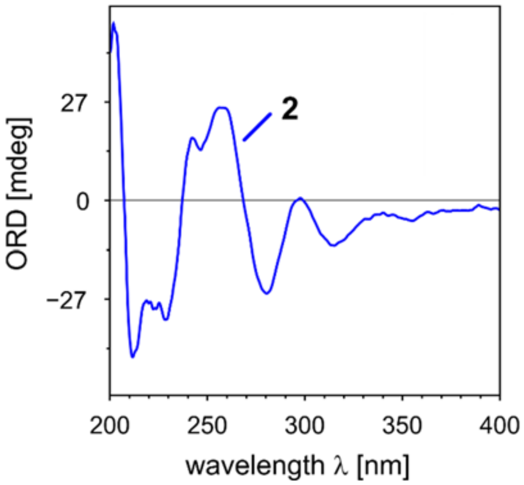

Figure S29. ORD-E spectrum of compound **2** in methanol.

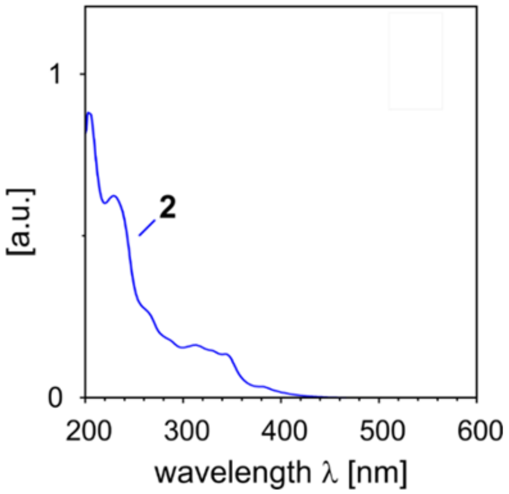

Figure S30. Offline UV spectrum of compound **2** in methanol.

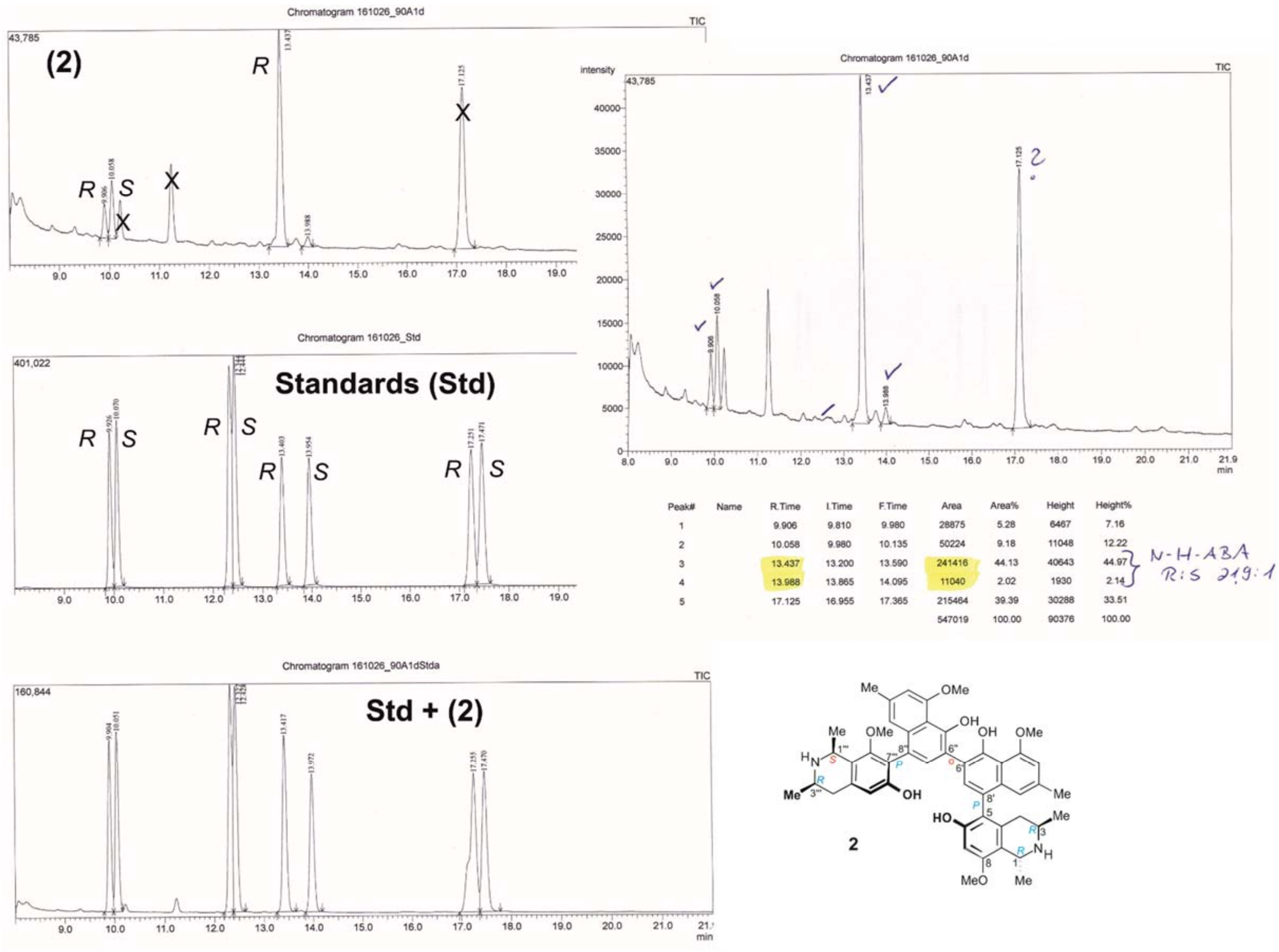

Peak#	Name	R.Time	I.Time	F.Time	Area	Area%	Height	Height%
1		9.906	9.810	9.980	28875	5.28	6467	7.16
2		10.058	9.980	10.135	50224	9.18	11048	12.22
3		13.437	13.200	13.590	241416	44.13	40643	44.97
4		13.988	13.865	14.095	11040	2.02	1930	2.14
5		17.125	16.955	17.365	215464	39.39	30288	33.51
					547019	100.00	90376	100.00

Figure S31. Oxidative degradation results of compound **2**.

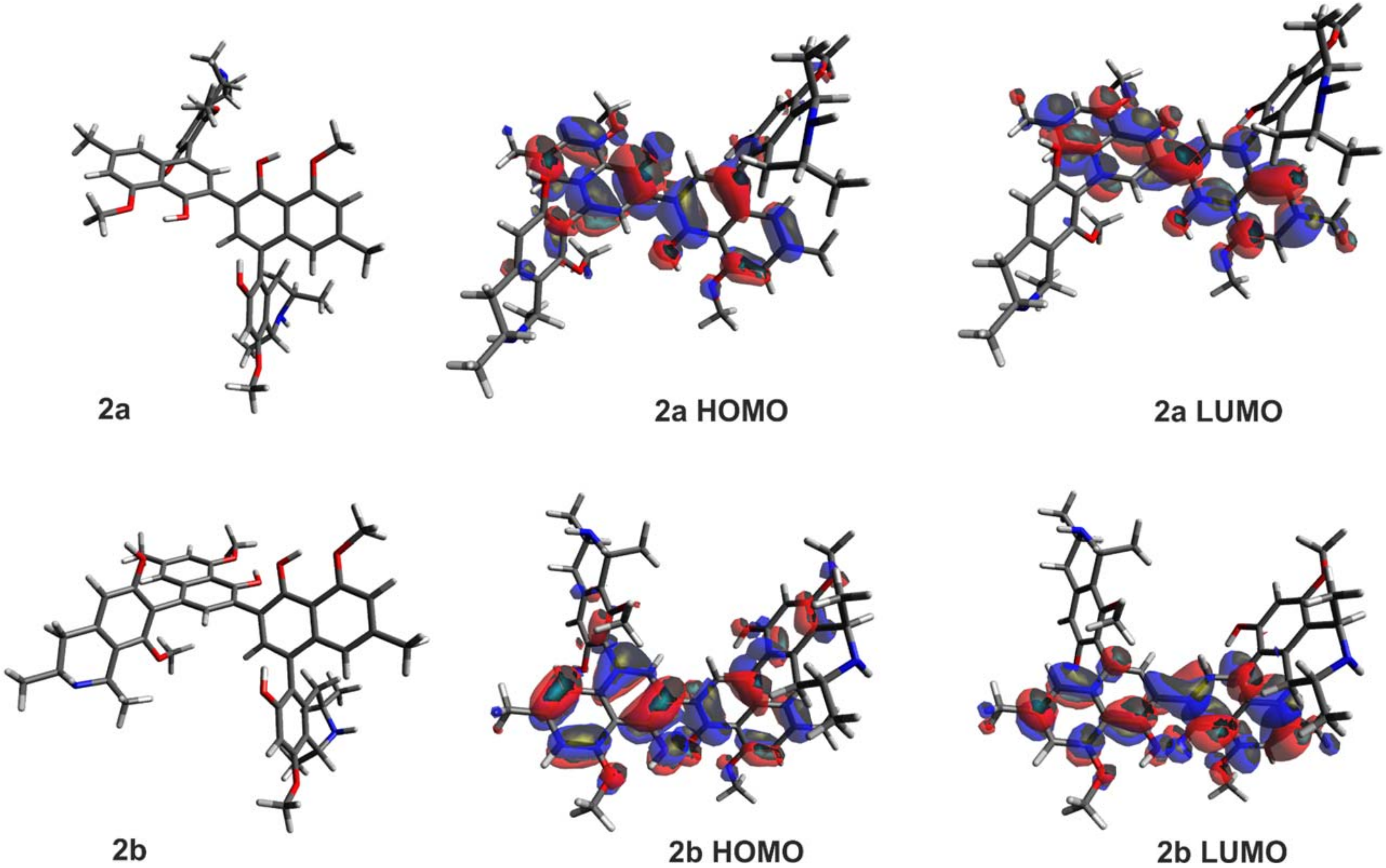

Figure S32. DFT-structural optimization of two conformers of compound **2** (**2a** and **2b**), and their HOMO and LUMO molecular orbitals. The most favorable conformer **2a** was found to have the highest HOMO-LUMO energy gap and the lowest total single point energy by DFT-calculations with B3LYP-D3/def2-TZVP.

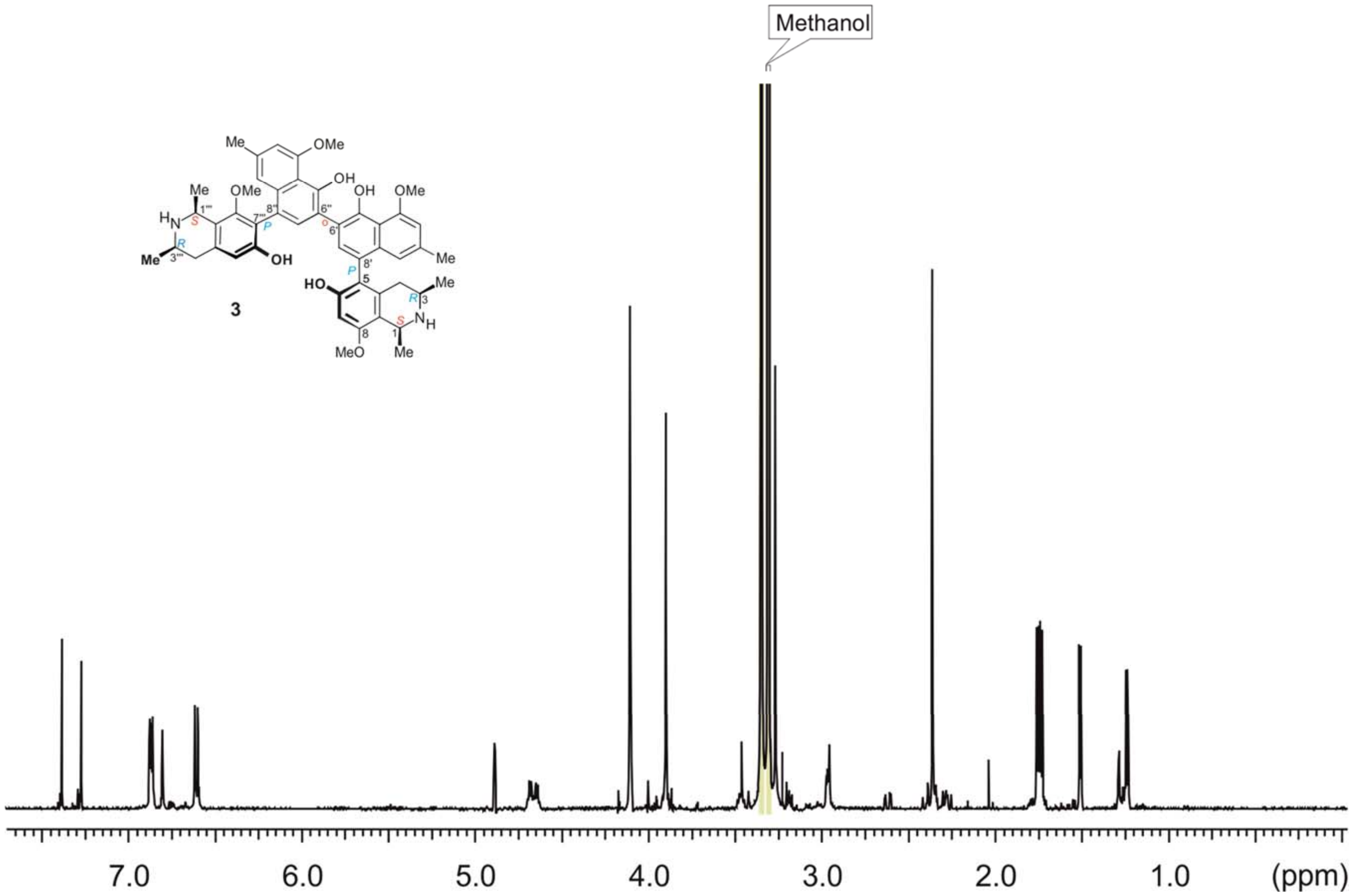

Figure S33a. Overall ^{1}H NMR spectrum of ealapasamine C **(3)** in methanol-d_4.

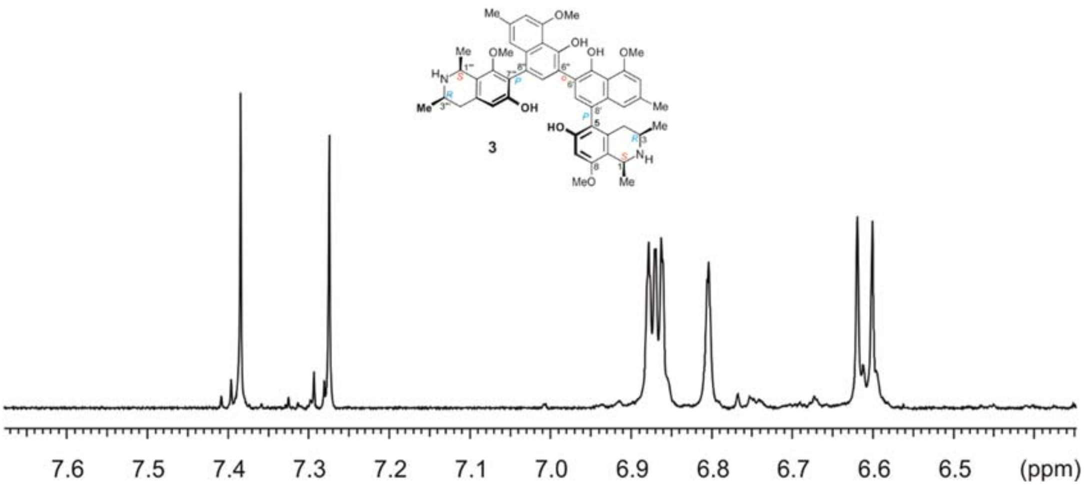

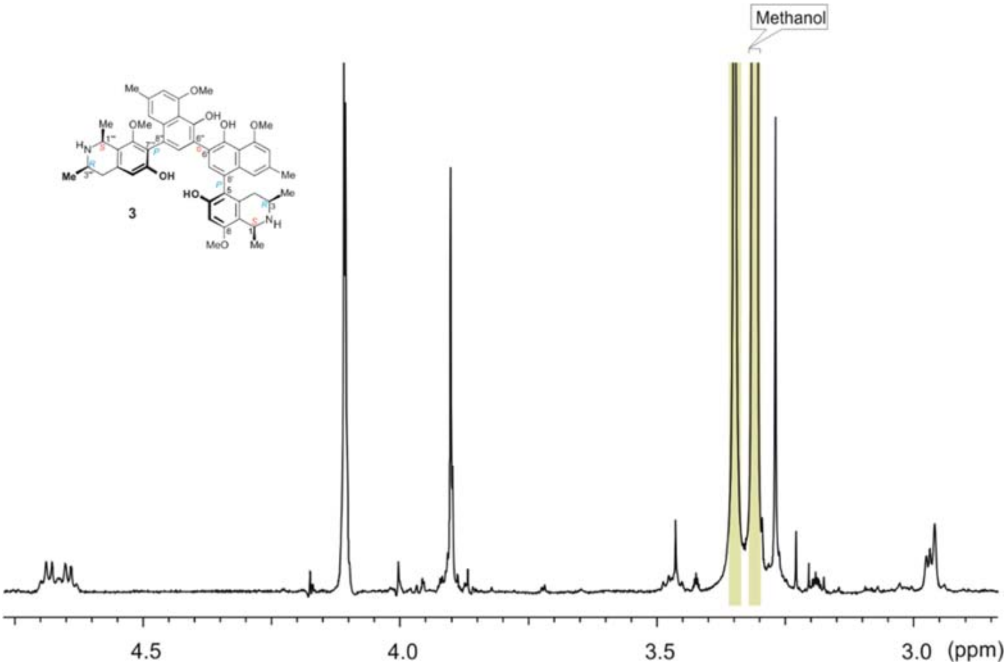

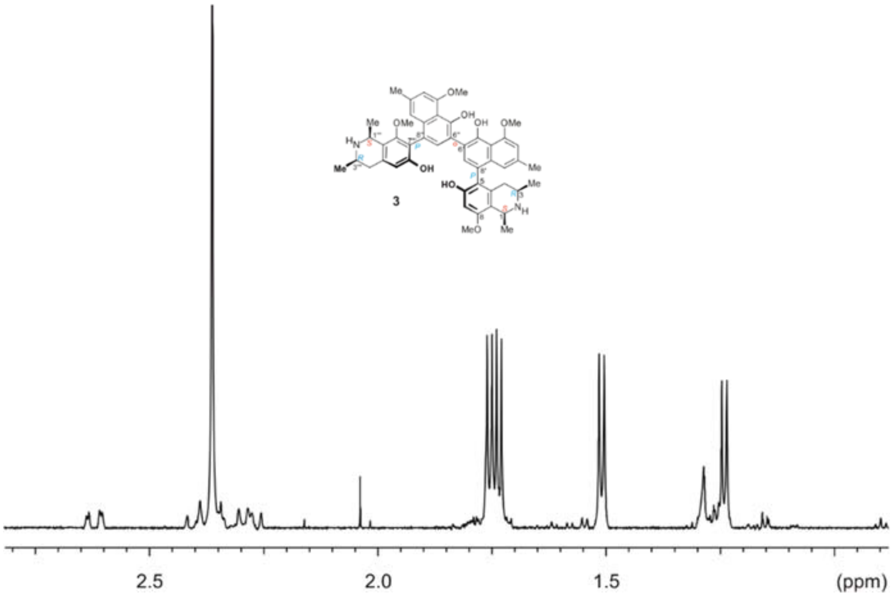

Figures S33b-d. Parts of the ^{1}H NMR spectrum of ealapasamine C **(3)** in methanol-d_4.

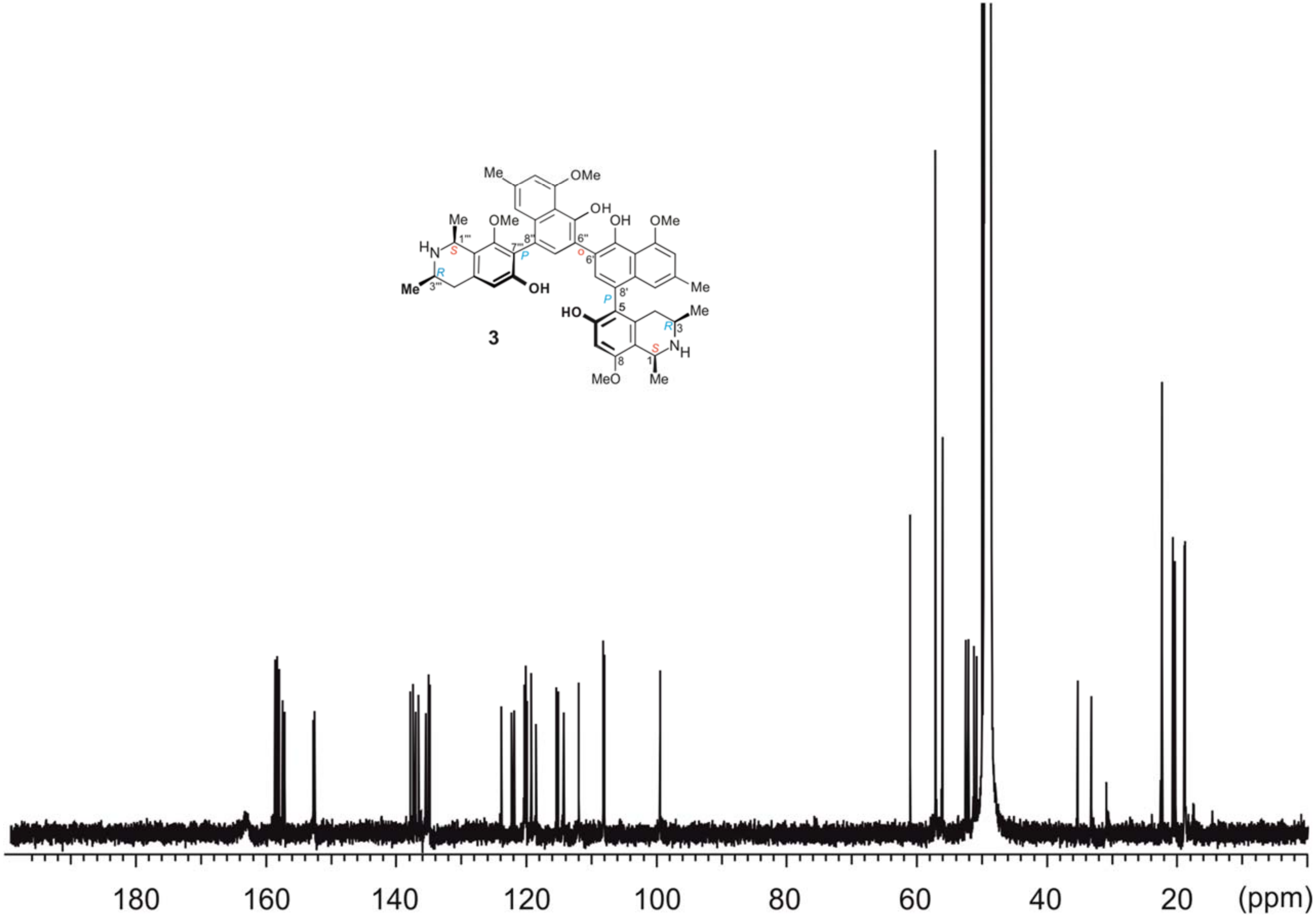

Figure S34a. Overall ^{13}C NMR spectrum of compound **3** in methanol-d_4.

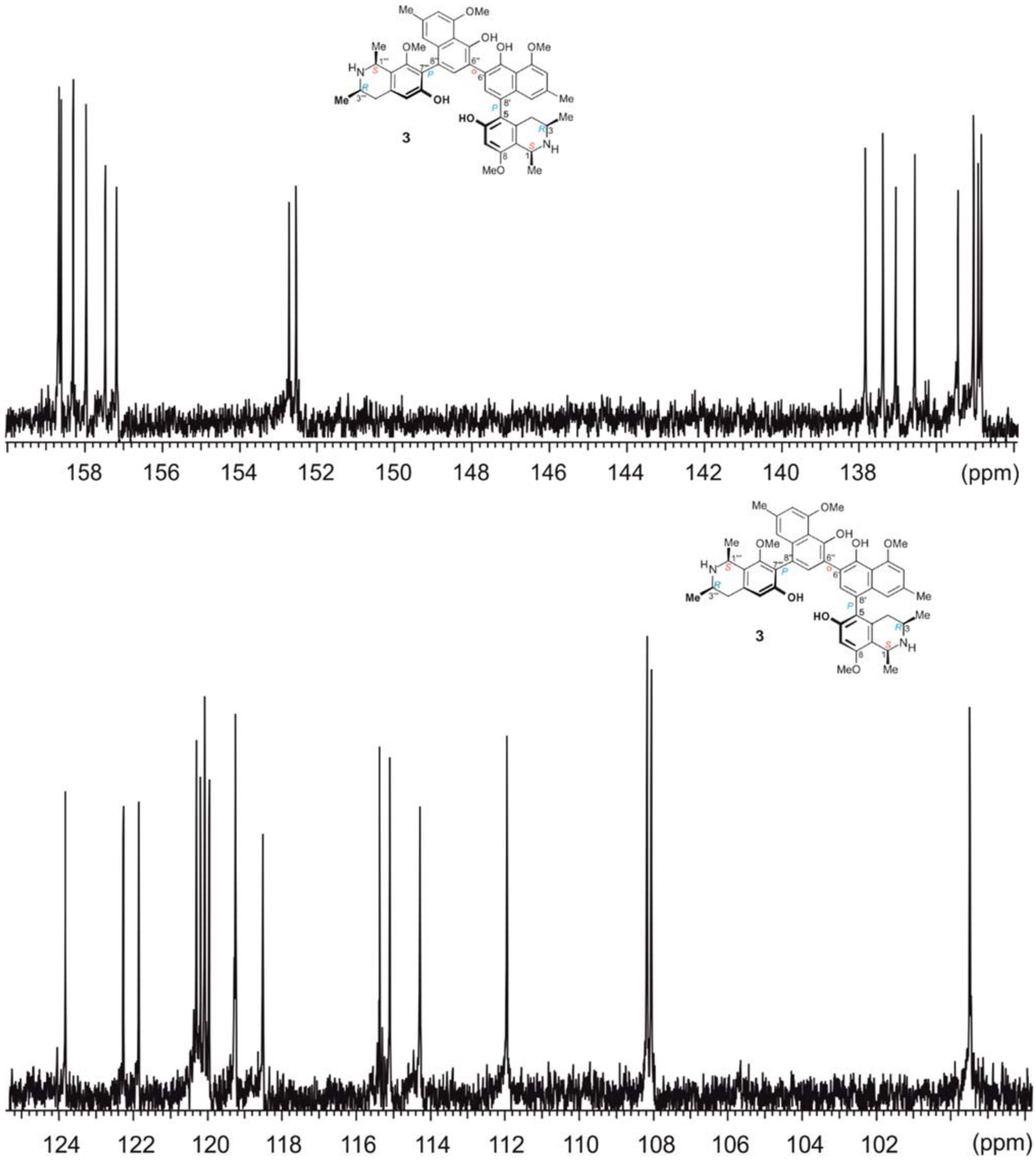

Figures S34b,c. Parts of the ^{13}C NMR spectrum of compound **3** in methanol-d_4.

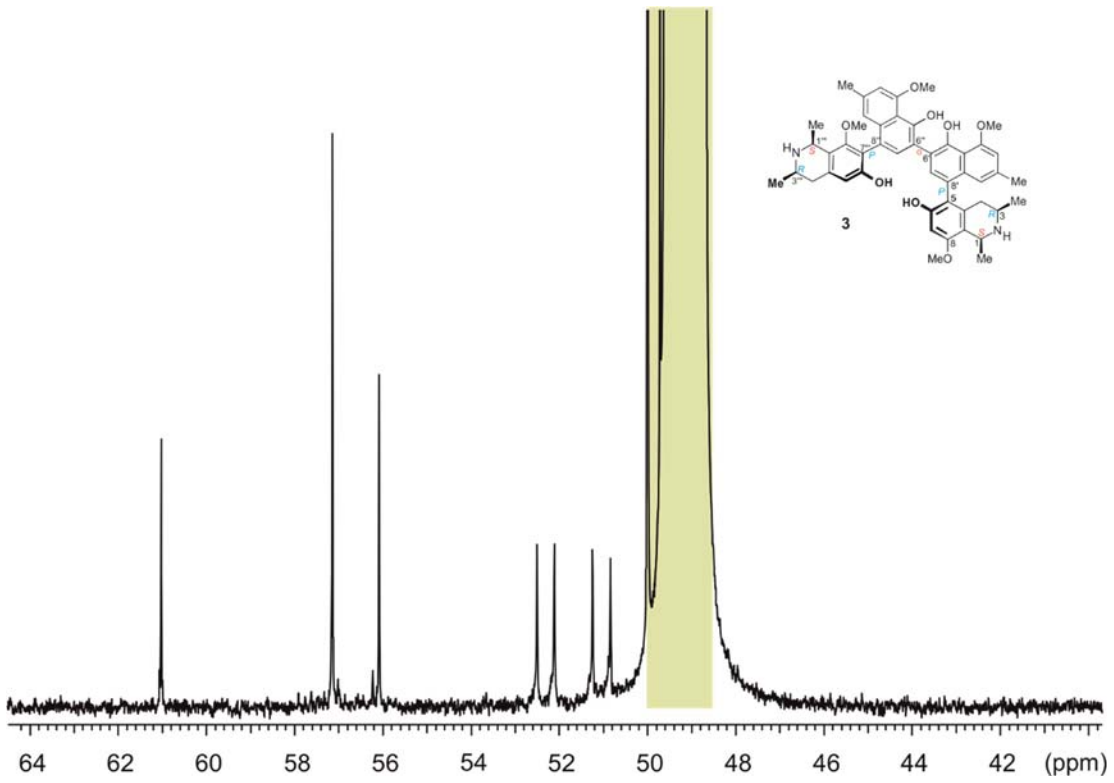

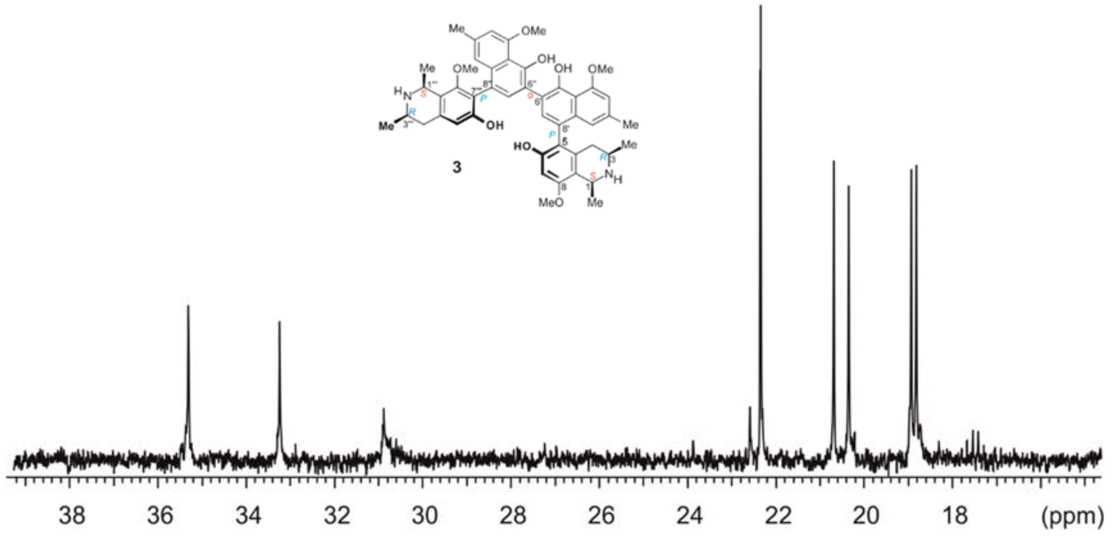

Figures S34d,e. Parts of the ^{13}C NMR spectrum of compound **3** in methanol-*d*$_4$.

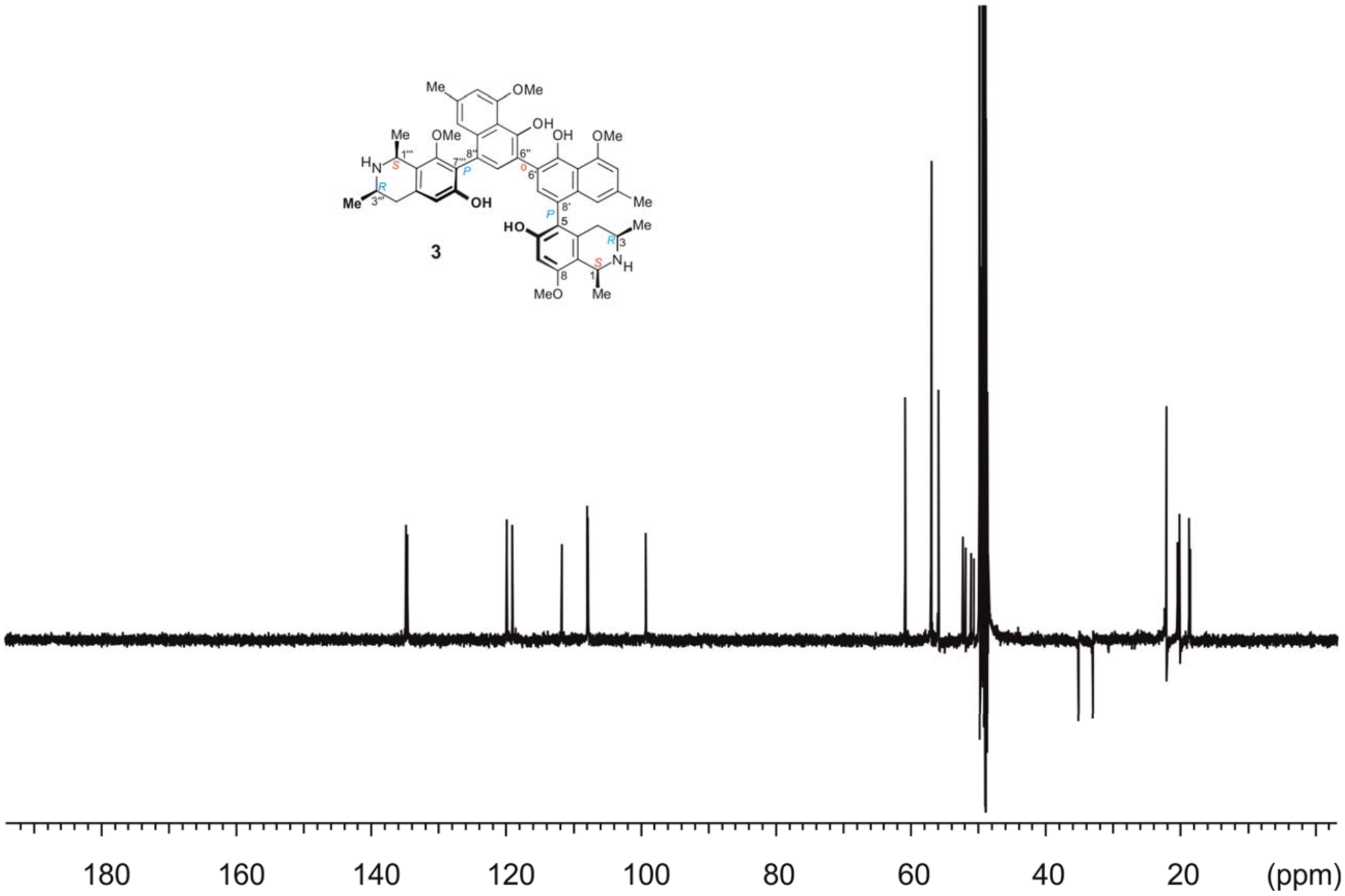

Figure S35. DEPT NMR spectrum of compound **3** in methanol-d_4.

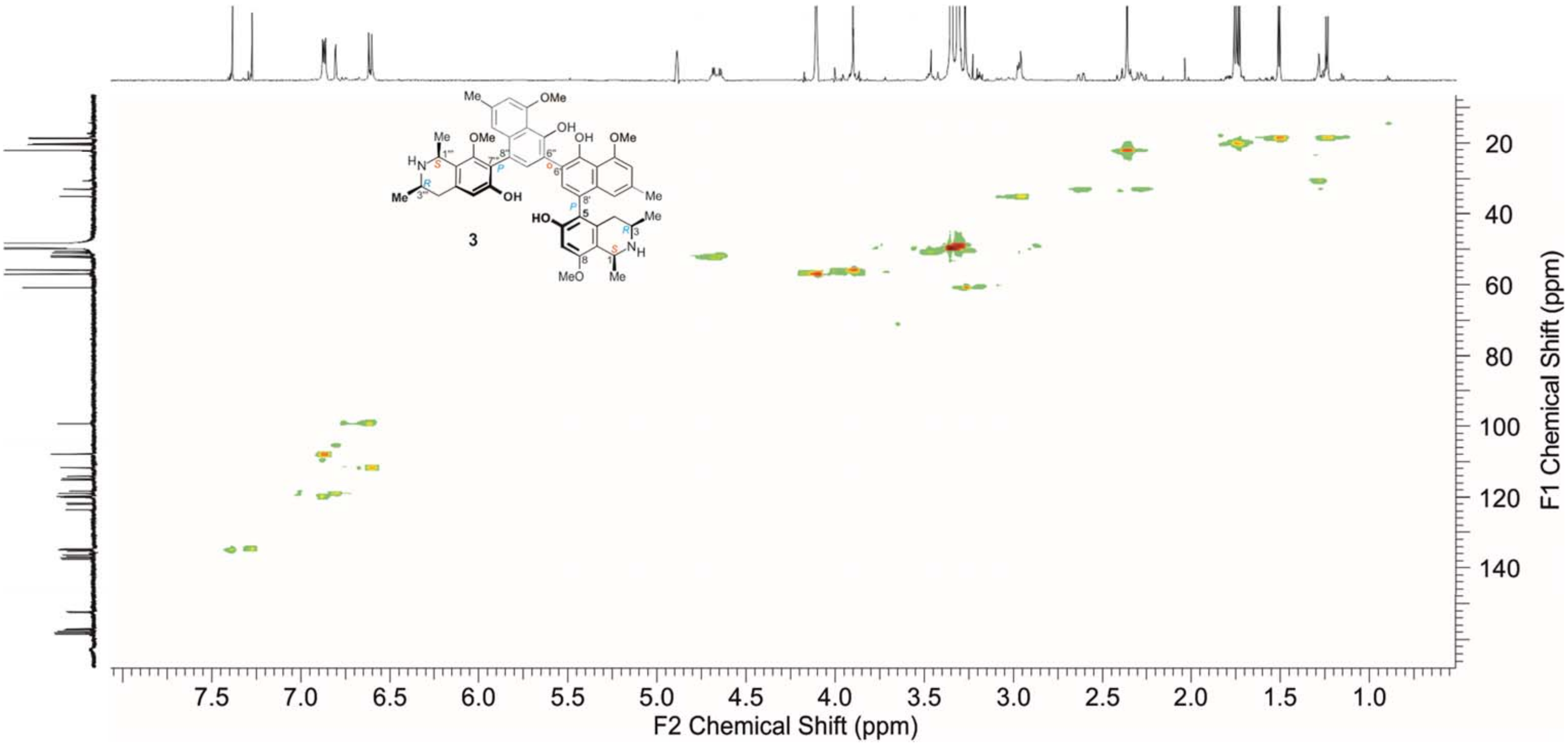

Figure S36a. Overall HSQC spectrum of compound **3** in methanol-d_4.

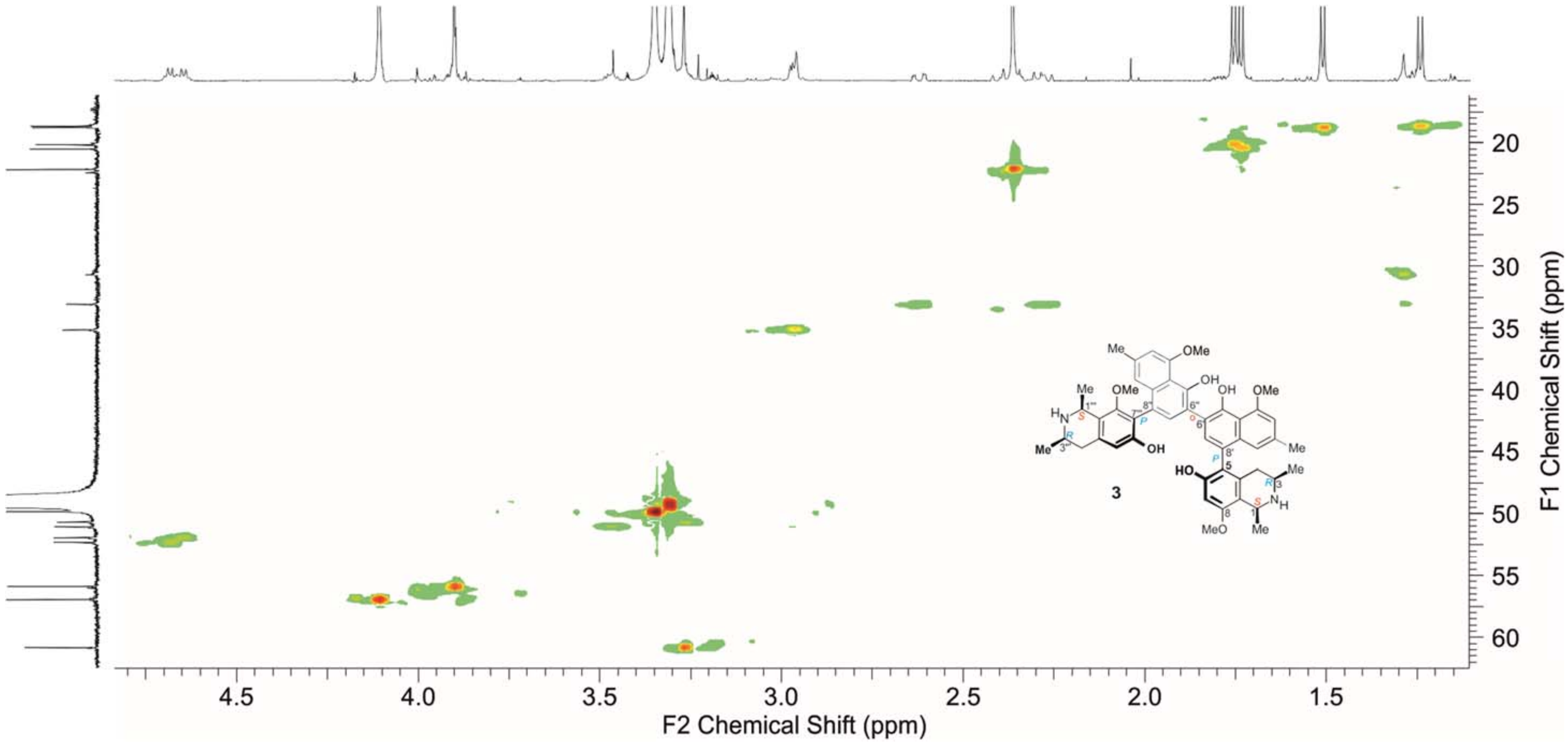

Figure S36b. Part of the HSQC spectrum of compound **3** in methanol-d_4.

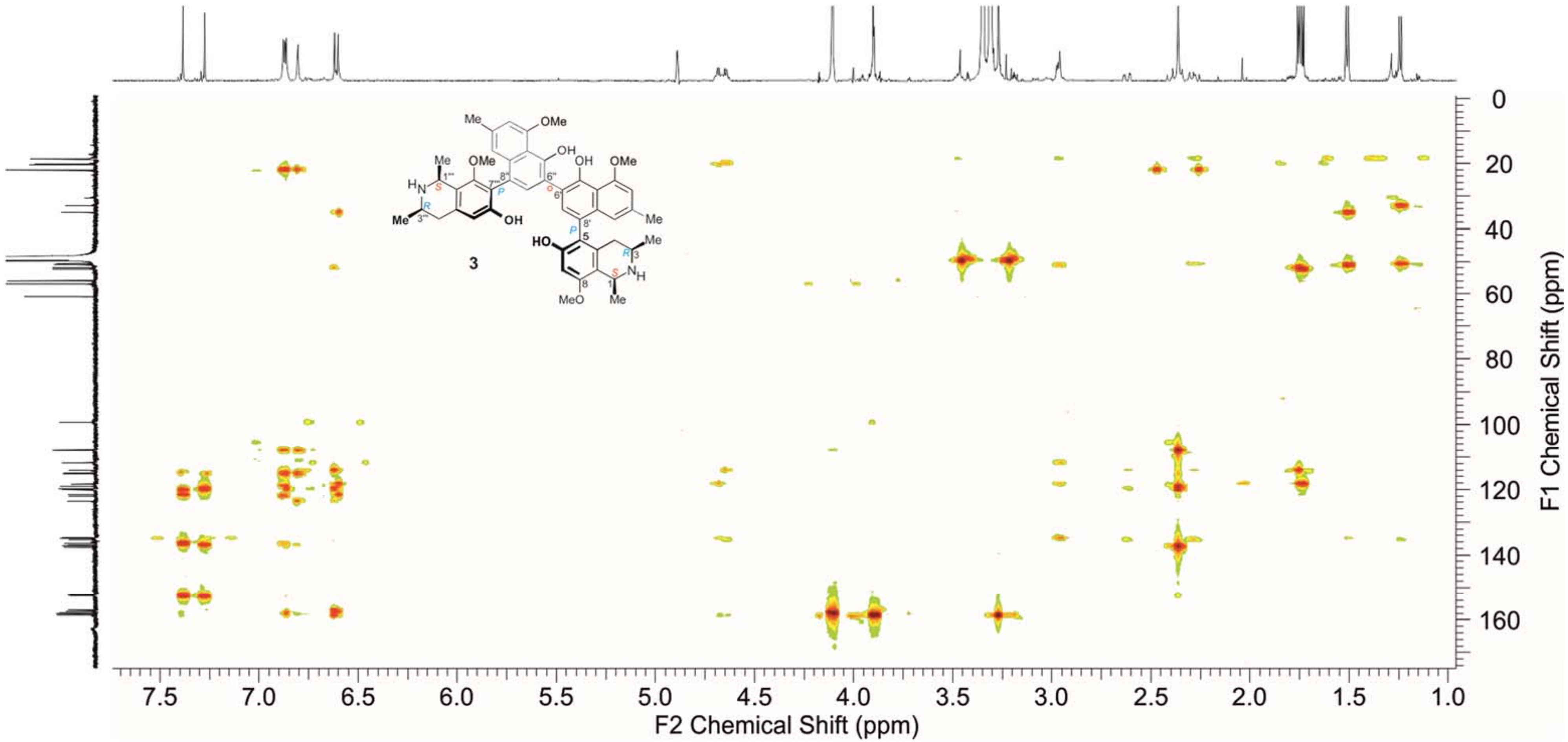

Figure S37. HMBC spectrum of compound **3** in methanol-d_4.

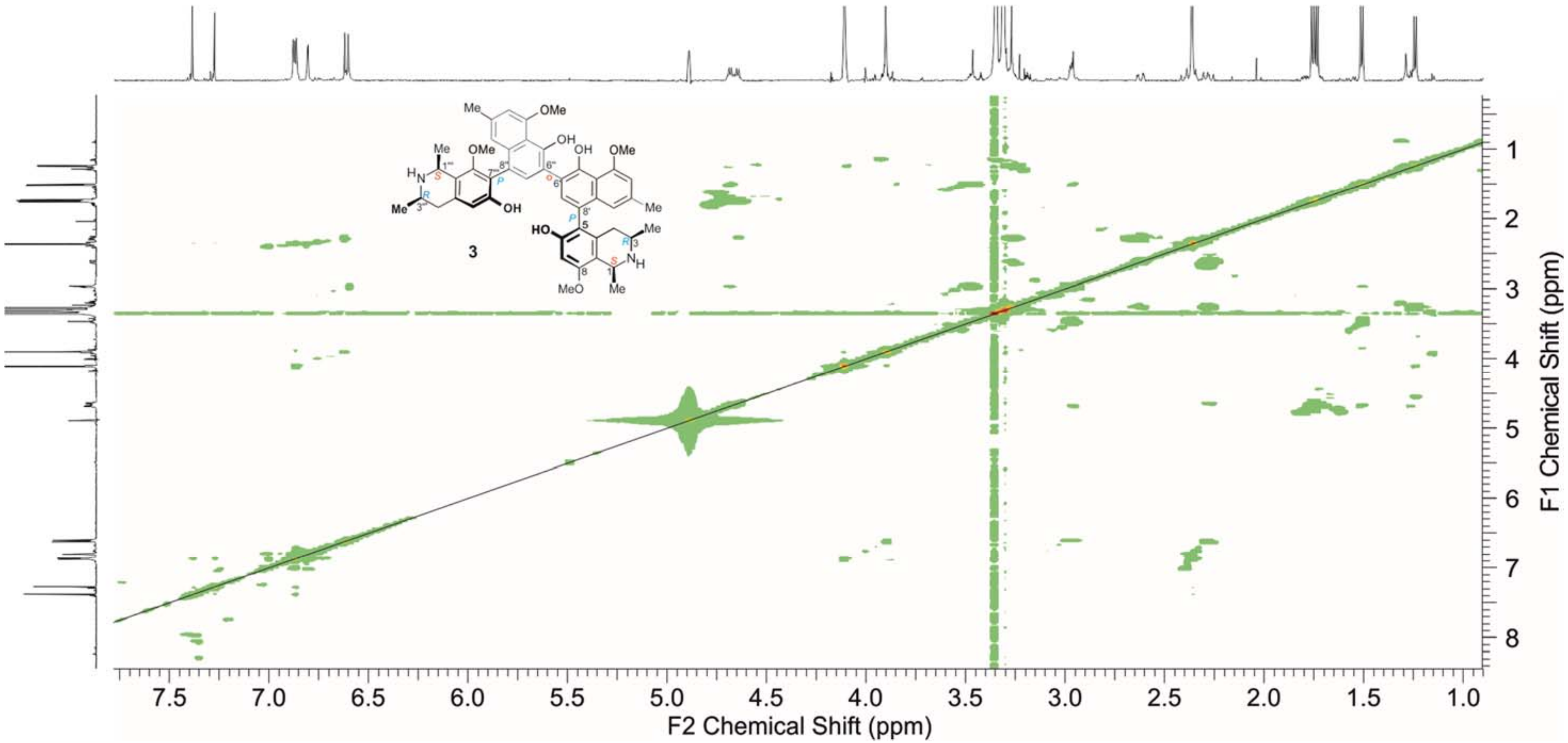

Figure S38. COSY spectrum of compound **3** in methanol-d_4.

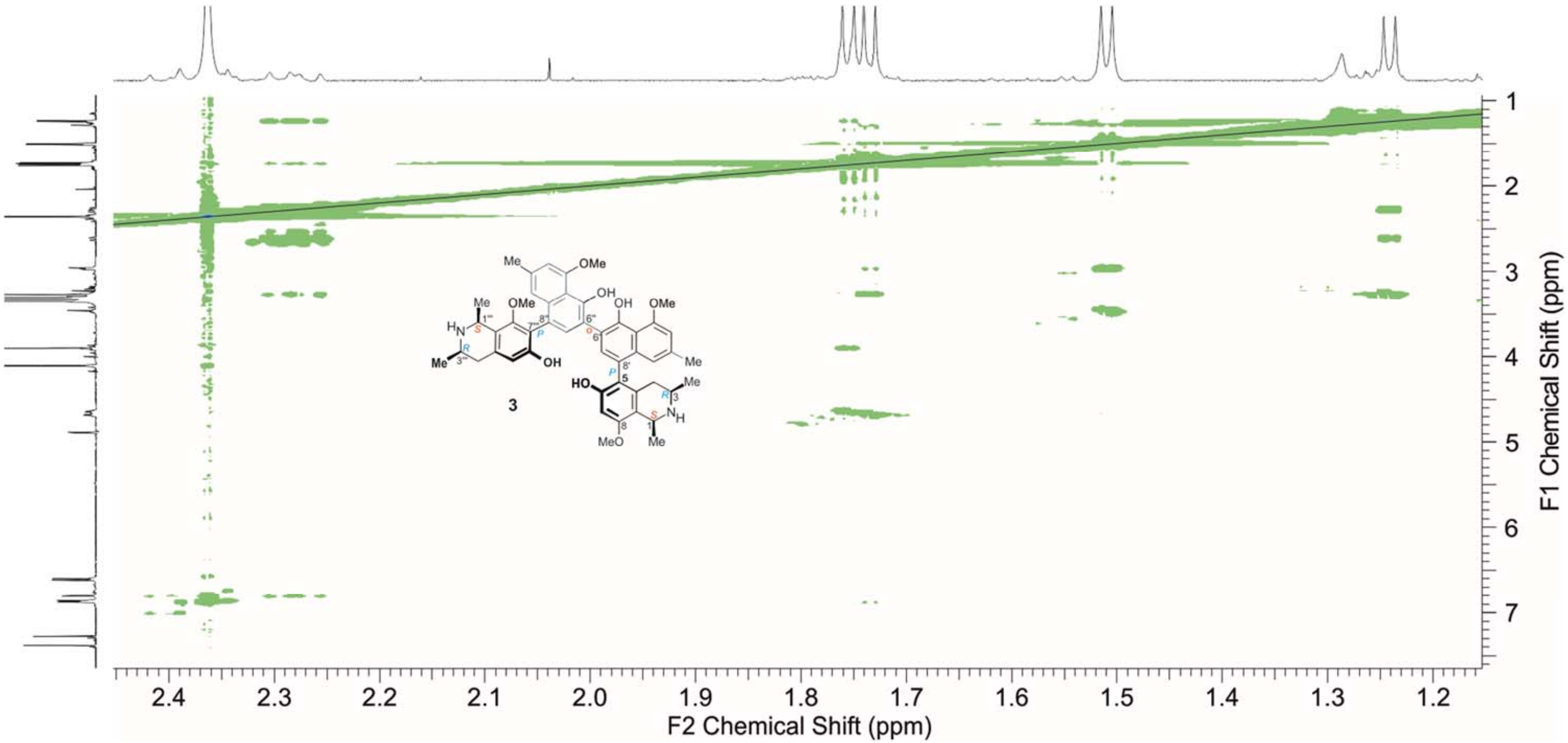

Figure S39a. Overall ROESY spectrum of compound **3** in methanol-d_4.

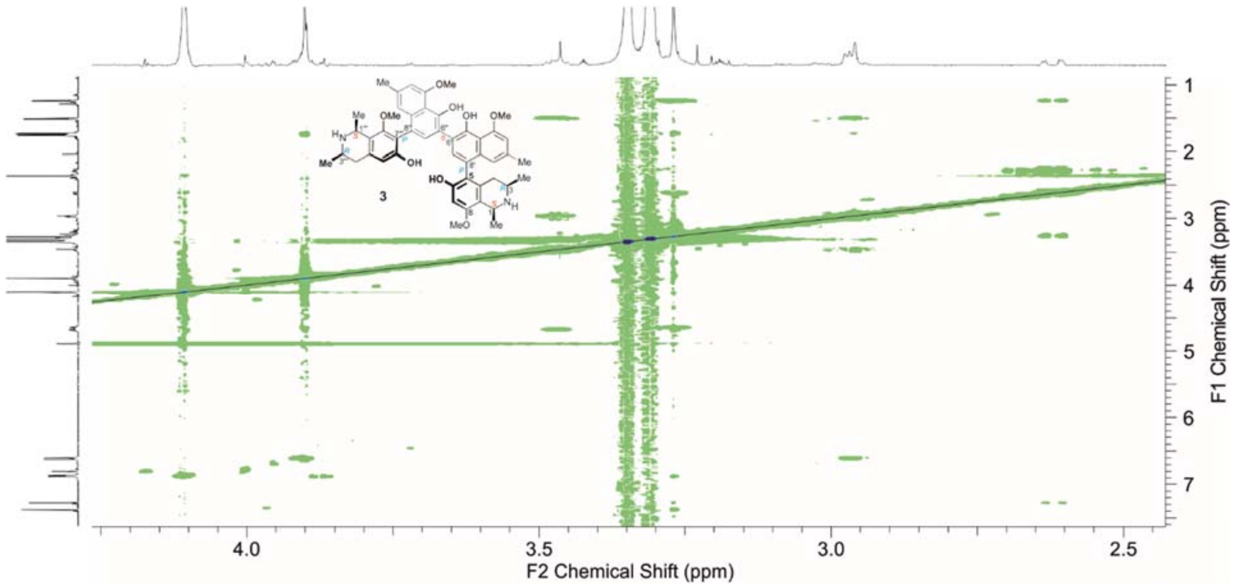

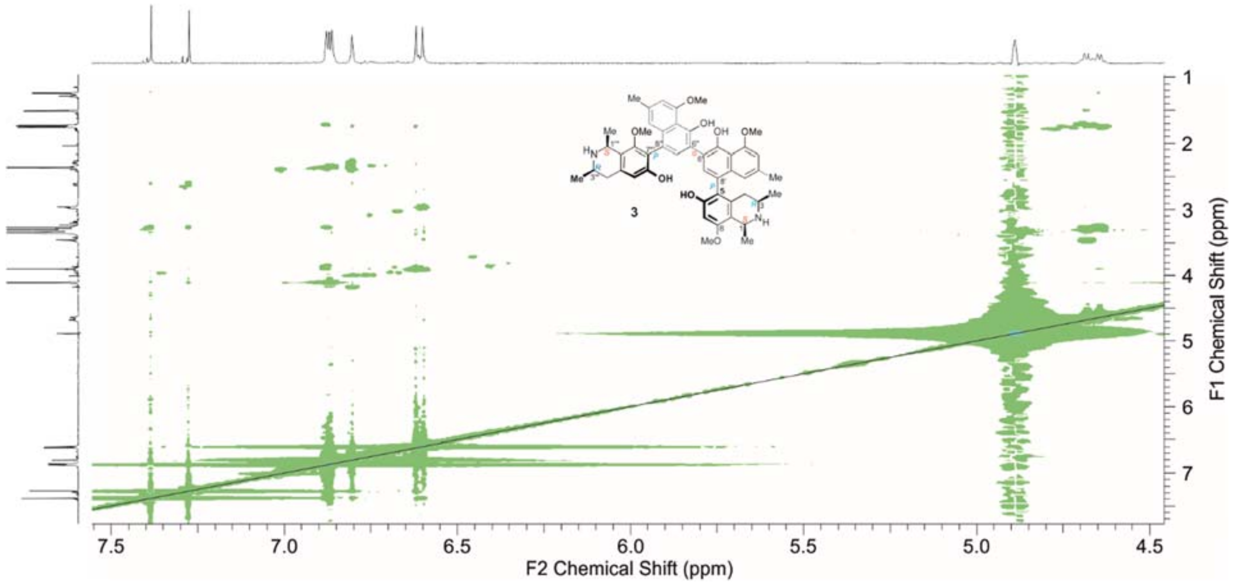

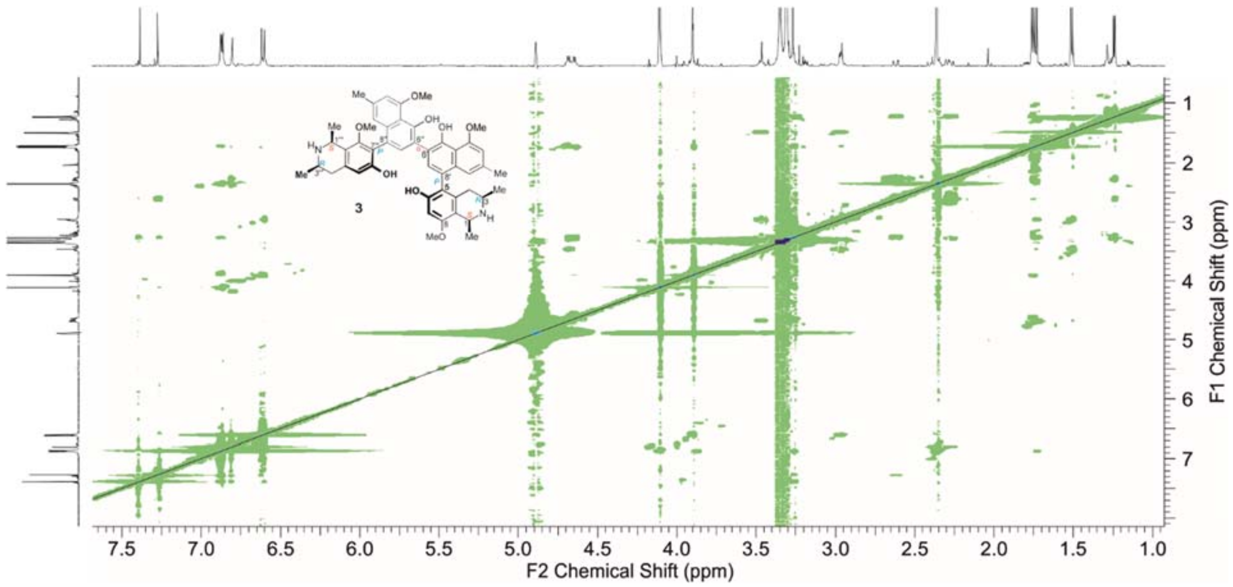

Figures S39b-d. Parts of the ROESY spectrum of compound **3** in methanol-d_4.

Acquisition Parameter

Source Type	ESI	Ion Polarity	Positive	Set Corrector Fill	43 V
Scan Range	n/a	Capillary Exit	180.0 V	Set Pulsar Pull	804 V
Scan Begin	50 m/z	Hexapole RF	280.0 V	Set Pulsar Push	807 V
Scan End	3000 m/z	Skimmer 1	100.0 V	Set Reflector	1700 V
		Hexapole 1	23.0 V	Set Flight Tube	8600 V
				Set Detector TOF	2160 V

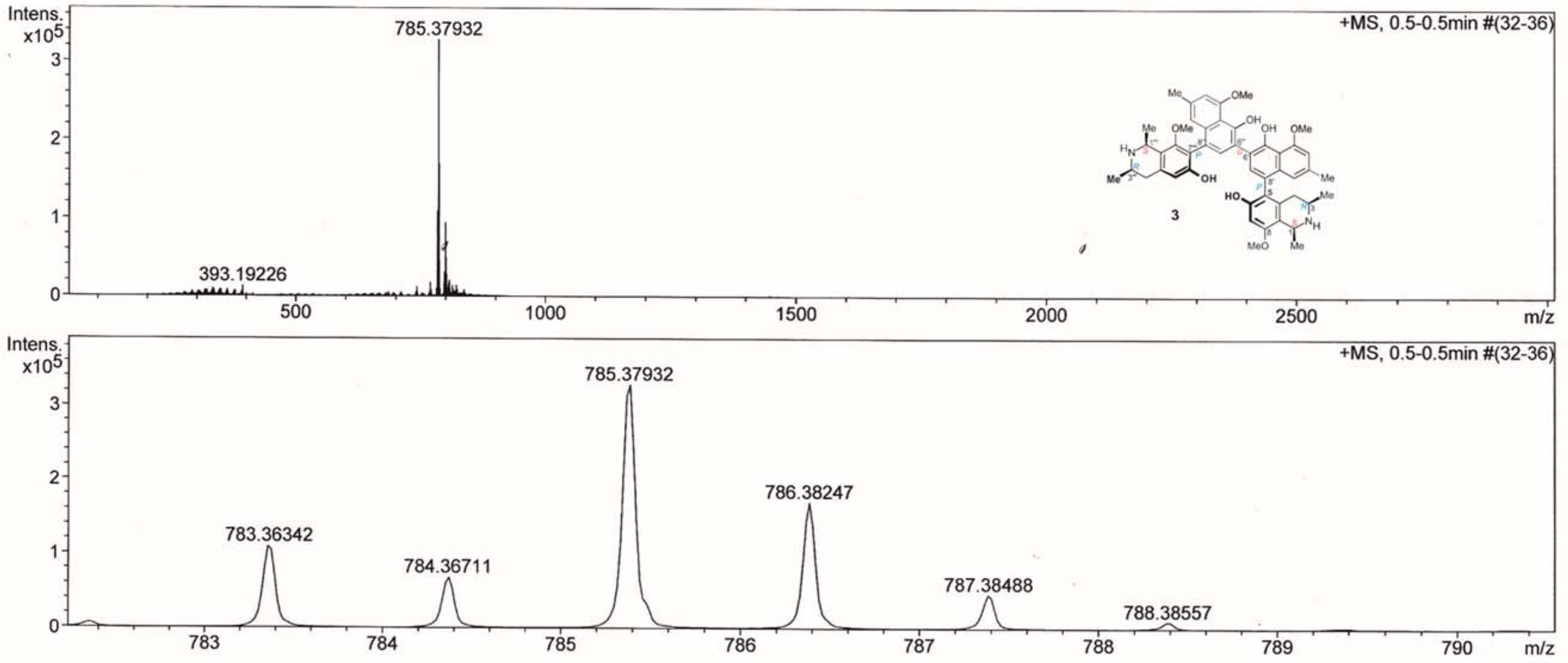

Sum Formula	Sigma	m/z	Err [ppm]	Mean Err [ppm]	rdb	N Rule	e⁻
C 48 H 53 N 2 O 8	0.01	785.37964	0.41	0.94	23.50	ok	even

Figure S40. HRESIMS spectrum of compound **3** in methanol.

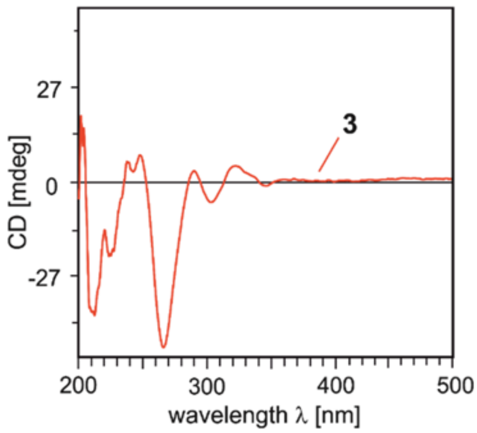

Figure S41. ECD spectrum of compound **3** in methanol.

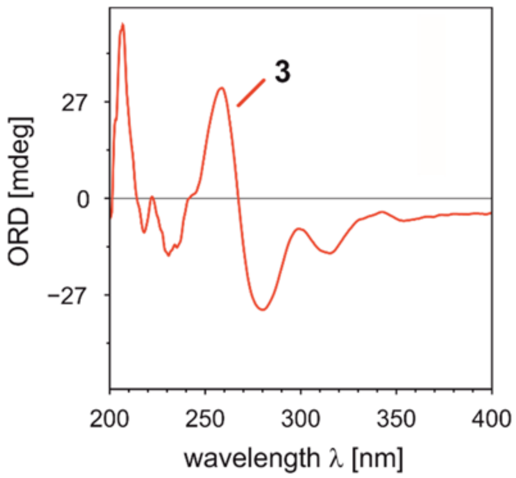

Figure S42. ORD-E spectrum of compound **3** in methanol.

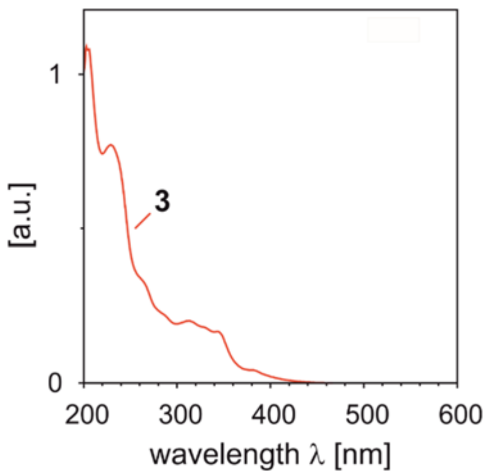

Figure S43. Offline UV spectrum of compound **3** in methanol.

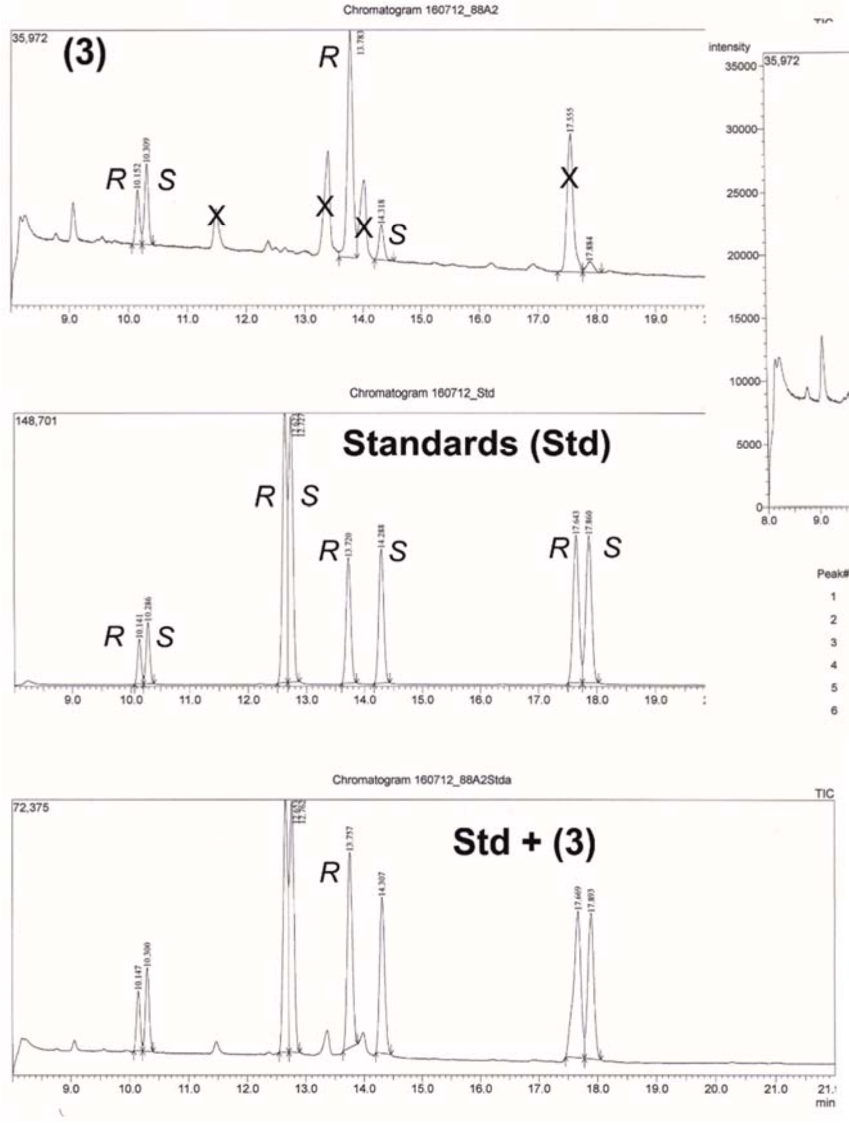

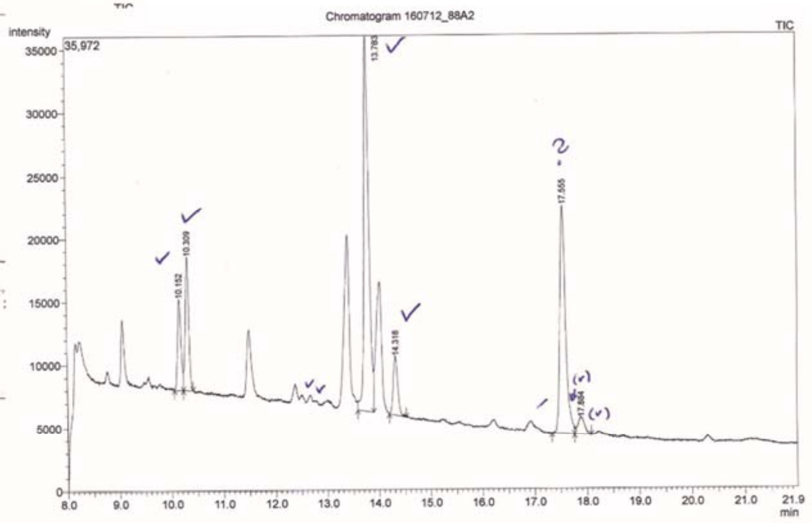

Peak#	Name	R.Time	I.Time	F.Time	Area	Area%	Height	Height%
1		10.152	10.060	10.230	33467	7.39	7234	10.10
2		10.309	10.230	10.415	48671	10.75	10584	14.78
3		13.783	13.585	13.895	193900	42.83	29692	41.45
4		14.318	14.195	14.525	27842	6.15	4636	6.47
5		17.555	17.335	17.765	136389	30.13	18055	25.21
6		17.884	17.765	18.085	12403	2.74	1430	2.00
					452672	100.00	71631	100.00

N-H-ABA R:S 7:1

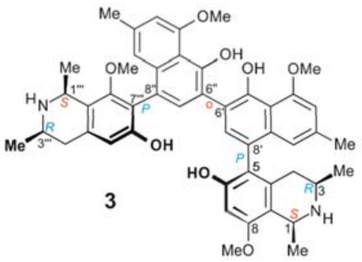

3

Figure S44. Oxidative degradation products of compound **3** (very diluted sample).

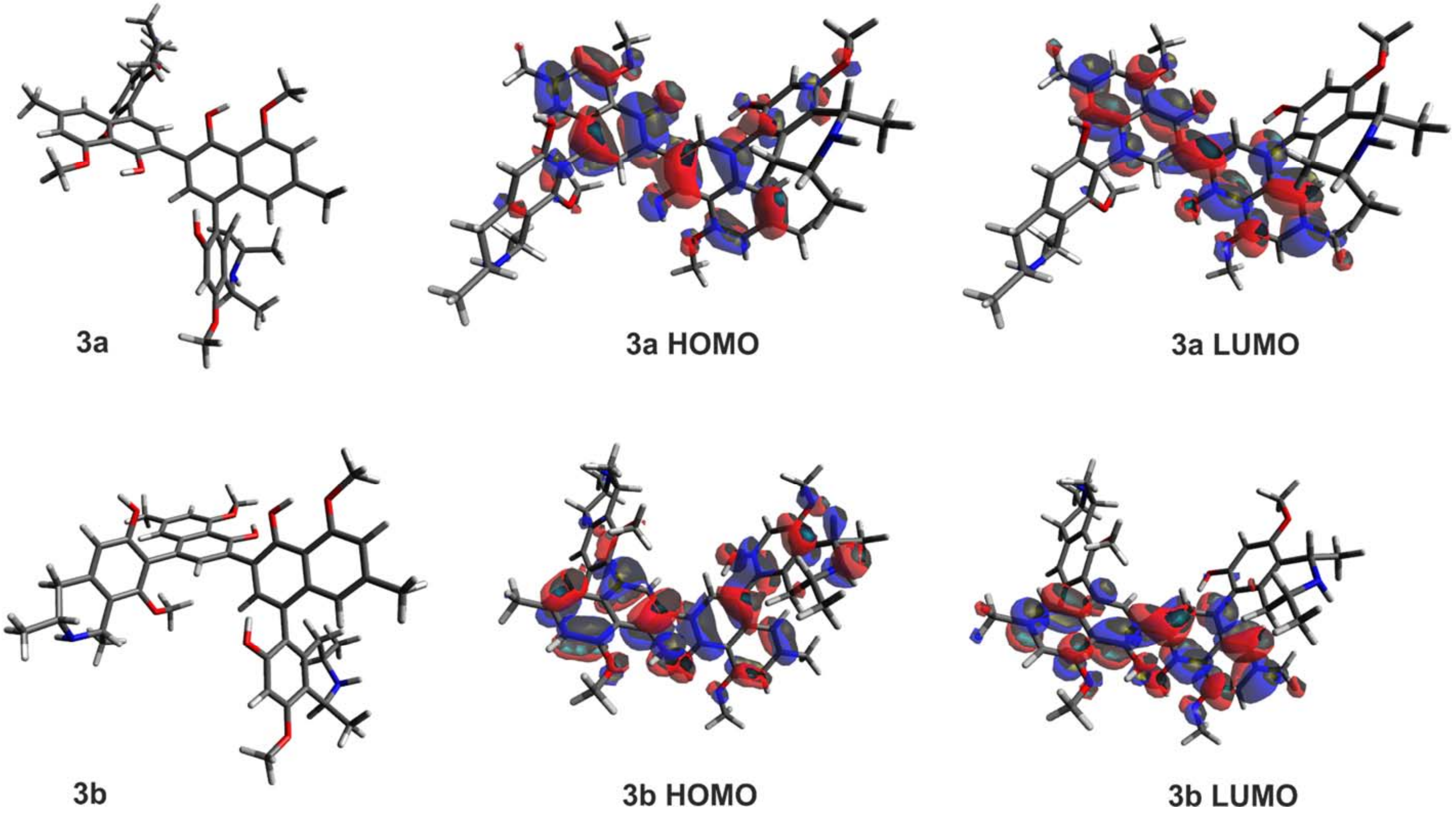

Figure S45. DFT-structural optimization of two conformers of compound **3** (**3a** and **3b**), and their HOMO and LUMO molecular orbitals. The most favorable conformer **3a** was found to have the highest HOMO-LUMO energy gap and the lowest total single point energy by DFT-calculations with B3LYP-D3/def2-TZVP.
